# An open-hardware platform for optogenetics and photobiology

**DOI:** 10.1038/srep35363

**Published:** 2016-11-02

**Authors:** Karl P. Gerhardt, Evan J. Olson, Sebastian M. Castillo-Hair, Lucas A. Hartsough, Brian P. Landry, Felix Ekness, Rayka Yokoo, Eric J. Gomez, Prabha Ramakrishnan, Junghae Suh, David F. Savage, Jeffrey J. Tabor

**Affiliations:** 1 Department of Bioengineering, Rice University, Houston, Texas, USA; 2 Graduate Program in Applied Physics, Rice University, Houston, Texas, USA; 3 Ph.D. Program in Systems, Synthetic, and Physical Biology, Rice University, Houston, Texas, USA; 4 Department of Molecular & Cell Biology, University of California, Berkeley, California, USA; 5 Department of Biosciences, Rice University, Houston, Texas, USA; 6 Department of Chemistry, University of California, California, USA

## Abstract

In optogenetics, researchers use light and genetically encoded photoreceptors to control biological processes with unmatched precision. However, outside of neuroscience, the impact of optogenetics has been limited by a lack of user-friendly, flexible, accessible hardware. Here, we engineer the Light Plate Apparatus (LPA), a device that can deliver two independent 310 to 1550 nm light signals to each well of a 24-well plate with intensity control over three orders of magnitude and millisecond resolution. Signals are programmed using an intuitive web tool named Iris. All components can be purchased for under $400 and the device can be assembled and calibrated by a non-expert in one day. We use the LPA to precisely control gene expression from blue, green, and red light responsive optogenetic tools in bacteria, yeast, and mammalian cells and simplify the entrainment of cyanobacterial circadian rhythm. The LPA dramatically reduces the entry barrier to optogenetics and photobiology experiments.

In 2005, a light activated microbial ion channel (opsin) was expressed in mammalian neurons and used for millisecond timescale control of their activity *in vitro*^1^. However, because no instrument existed for delivering the necessary intensity of light to specific brain regions in live animals without major side effects, optogenetics contributed few neurobiological insights between 2005 and 2009^2^. During this period, the optical neural interface (ONI) – a brain-implantable optical fiber with a laser diode light source – was developed. The ONI was rapidly adopted by the neuroscience community and combined with opsins and other photoreceptors, resulting in a wave of breakthroughs in a short time period^2^.

In 2002, a red/far red light-reversible transcriptional regulatory (promoter) system was developed for optical control of gene expression in *S. cerevisiae*^3^. In the 14 years that have followed, photoreceptors with diverse spectral properties have been used to control a remarkable range of cell biological processes in mechanistically tractable model organisms. For example, light-switchable promoter systems have been engineered in *E. coli*^4^,^5^,^6^,^7^,^8^,^9^, cyanobacteria^10^, yeast^11^,^12^, mammalian cells^13^,^14^,^15^,^16^,^17^,^18^,^19^,^20^,^21^,^22^, fruit flies^23^, zebrafish^19^,^24^ and plants^25^. Translation^26^,^27^, proteolysis^28^, membrane recruitment^11^,^29^, signaling^13^,^29^,^30^,^31^,^32^,^33^,^34^,^35^,^36^, ER-to-cytoplasm^37^ and nuclear^38^,^39^,^40^,^41^ translocation, and genome editing^11^,^42^,^43^ have also been placed under optogenetic control.

However, no optical hardware has been developed to enable the broad research community to properly utilize these non-neural optogenetic tools, limiting their impact. For example, we recently engineered the Light Tube Array (LTA), a light emitting diode (LED)-based device that exposes 64 shaking incubated culture tubes to programmable light signals with an intensity range over three orders of magnitude and millisecond resolution^44^. Though the LTA enables unrivaled control of gene expression dynamics^44^, construction requires custom machined components, specialized assembly tools, and knowledge of electronic system design and programming is done in computer language. Additionally, experiments are not scalable due to the large instrument size (0.02 m^3^) and requirement for connection to an external computer. Furthermore, the tubes are costly (~$0.10/each) and restrict experiments to suspension culture organisms such as bacteria and yeast. Finally, the LEDs are permanently soldered, limiting optical flexibility. In another example, Moglich and coworkers modified the injection port of a Tecan microplate reader with an optical fiber and eight LEDs from 385–850 nm^45^. While this clever design enables programmable sample illumination via the commercial software, the Tecan instrument costs ~$40,000. Other recent designs^13^,^39^,^46^,^47^,^48^,^49^,^50^ suffer various limitations and have not found widespread adoption.

We have designed the LPA for compatibility with a wide range of optogenetics and photobiology experiments and model organisms, low device and consumable costs, high scalability and throughput, and accessibility by laboratories without hardware expertise. We demonstrate its capabilities by recapitulating and extending our LTA results in *E. coli*, performing yeast and mammalian optogenetic gene expression control experiments, and easing the entrainment of cyanobacterial circadian rhythm. Additionally, because the LPA and Iris are open source, they may be freely modified and extended by the community for additional functionalities.

## Results

### LPA design and assembly

**Figure 1:**
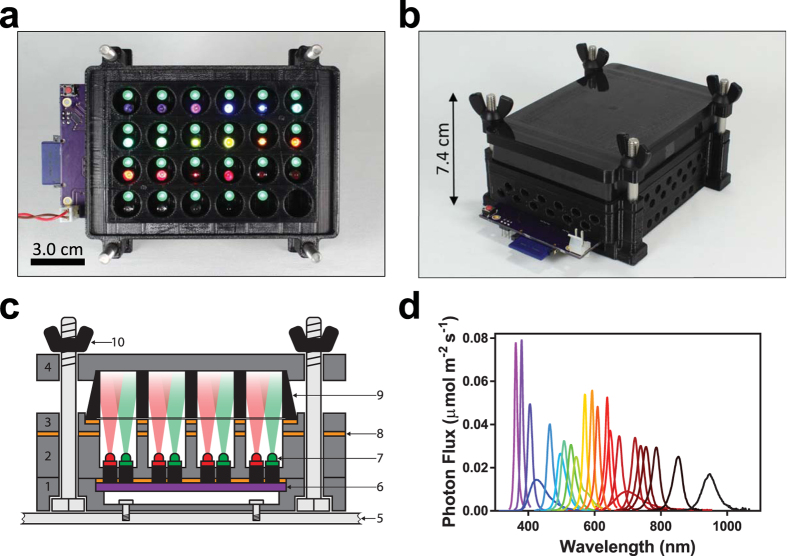
The LPA. **(a)** Top-down view of an LPA lacking the 24-well plate and plate lid. In the configuration shown, the device contains a 535 nm LED in each top position and a variable wavelength LED (364–947 nm) in each bottom position. The SD card (blue), circuit board (purple), reset button (red) and power cord (red/black wires) are visible. **(b)** Fully assembled LPA. **(c)** Schematic cross-section showing the light path from the LEDs to the culture plate wells. (1) mounting plate, (2) LED spacer, (3) plate adapter, (4) plate lid, (5) incubator platform, (6) PCB, (7) LED atop LED socket, (8) gasket, (9) 24 well plate, (10) wing nut assembled with mounting bolt. **(d)** Spectra of 22 LEDs (Supplementary Table S5) used in this study measured and calibrated using a spectrophotometer and probe adapter (Supplementary Figs S7 and S20, and Supplementary Methods).

The core of the LPA is a printed circuit board (PCB) (Supplementary Fig. S1) outfitted with a Secure Digital (SD) card reader (Supplementary Figs S1 and S2), an Atmel ATMega328a microcontroller (Supplementary Figs S1 and S3), 3 LED drivers (Supplementary Figs S1 and S4), 48 solder-free LED sockets (Supplementary Fig. S1), a power regulating circuit (Supplementary Fig. S5), and other standard electronics components (Supplementary Table S1). The only external connection is to a 5V power supply (Fig. 1a and Supplementary Fig. S6). The unpopulated PCB can be ordered from a commercial supplier using the provided fabrication files (Supplementary Methods, Supplementary Files). The PCB can be assembled (populated with electronic components) via do-it-yourself (DIY) or commercial soldering procedures (Supplementary Methods and Supplementary Table S2). We recommend DIY installation of the LED sockets using a 3D printed socket alignment tool (Supplementary Fig. S7 and Supplementary Methods) to ensure consistent geometries and illumination across the wells. We have provided a video tutorial demonstrating PCB assembly (Supplementary Video S1).

The microcontroller is programmed to convert an Iris-generated file stored on the SD card to LED driver control signals. (Supplementary Figs S8–S14, Supplementary Tables S3 and S4, and Supplementary Notes). Each LED driver individually regulates the intensity of 16 LEDs over 4096 levels with a 1 ms refresh rate via 12-bit pulse width modulation of output current up to 20 mA. The output of each driver, and thus LED intensity, can be further controlled with 6-bit resolution by adjusting the device’s dot correction settings, Supplementary Methods).

Each socket secures a 5 mm through-hole LED via a friction fit (Supplementary Fig. S15). These LEDs are commercially available with wavelengths from 310 to 1550 nm (Supplementary Table S5), enabling plug-and-play optical reconfiguration (Supplementary Methods). The 20 mA driver output permits full range control of most 5 mm LEDs.

A 3D printed chassis houses the assembled PCB and 24-well plate (Fig. 1a–c). The chassis comprises i) a mounting plate, ii) LED spacer, iii) plate adapter, and iv) plate lid (Supplementary Fig. S16). We have designed 4 mounting plates, ensuring compatibility with most shaker platforms. The mounting plates contain recessed holes (Fig. 1c) for upward-facing bolts that align and stack the remaining 3 modules. The LED spacer creates a fixed distance between each LED socket pair and overhead well, and optical isolation between wells. The spacer is mated with the PCB and the resulting assembly stacked atop the mounting plate. The plate adapter is placed atop the spacer. A black-walled, transparent plastic bottomed 24 well plate (Supplementary Fig. S17 and Supplementary Table S6) is aligned and held in place by the plate adapter. An adhesive foil plate cover (Supplementary Table S6) provides a sealed environment for each well. Laser cut nitrile gaskets (Supplementary Fig. S18 and Supplementary Methods) are placed at each of the 3 interfaces above the PCB to reduce optical contamination. Finally, the lid is placed atop the plate and the complete assembly is secured with wing nuts (Fig. 1b and Supplementary Table S6). All chassis modules can be 3D printed using supplied files (Supplementary Files, Supplementary Methods). We have provided a written procedure and instructional video demonstrating LPA assembly (Supplementary Methods and Supplementary Video S2).

### LPA programming with Iris

**Figure 2:**
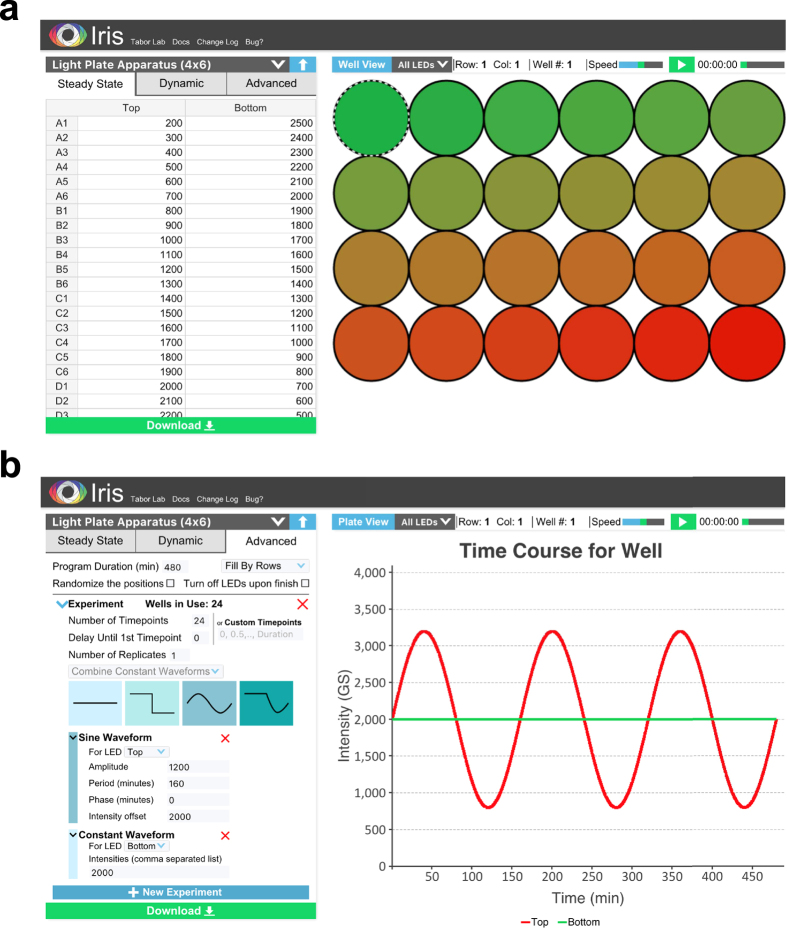
Iris. **(a)** Steady State Mode. Constant light functions are specified by entering the desired greyscale intensity (0–4095) of up to all 48 LEDs in the Input Panel spreadsheet (left). The download button is shown at the bottom of the Input Panel. In Plate View mode (shown), the Simulation Panel (right) displays a schematic visualization of the output intensity of all 48 LEDs. The top and bottom LEDs are visualized as red and green, respectively, regardless of the actual LEDs used. (**b**) Advanced Mode. Constant and dynamic light functions are specified in the Input Panel. In the latter case, Iris automatically runs a staggered-start algorithm (Supplementary Fig. 19). In Well View mode (shown), the Simulation Panel (right) visualizes the output of the top and bottom LEDs in a given well over the duration of the experiment. The Simulation Panel also plays movies of specified light functions.

Iris can be freely accessed at http://iris.taborlab.rice.edu or run locally using the provided source code (Supplementary Notes). Light programs are specified in Steady State, Dynamic, or Advanced Modes (SSM, DM, AM). In SSM, time-invariant intensities for each LED are entered within an Input Panel (Fig. 2a, left). In DM, constant, step, sinusoidal, or piecewise light signals are specified using parameters such as initial and final intensity, time of step, or wave period, amplitude, and offset (Supplementary Notes). The same signal is run by all 24 top LEDs, and the same, or a second signal can be run by all 24 bottom LEDs. AM (Fig. 2b, left) implements DM signals via our recent staggered-start protocol, which allows kinetic experiments to be performed with far less sample handling^44^ (Supplementary Fig. S19, Supplementary Table S7). The light signal run in each well can be randomized (Supplementary Notes) to mitigate any well-to-well variability. We have provided videos demonstrating Iris light signal programming in all 3 modes (Supplementary Videos S3–S5).

Light programs can be viewed and debugged at the plate (Fig. 2a, right) or individual well (Fig. 2b, right) levels using the Simulation Panel. When the program is satisfactory, the download button (Fig. 2a,b) is pressed. Iris then generates a zip file containing i) a device-readable binary file (.lpf, Supplementary Table S8) used to run the LPA, ii) a session file for reloading a program into Iris at a later time, and iii) a CSV file containing user-readable well randomization information. The .lpf is then transferred to an SD card, which is inserted into the LPA, and the Reset Button (Supplementary Fig. S1) is pressed to run the light program. Included Python scripts can be used to automate .lpf design (Supplementary Files, Supplementary Notes), increasing throughput.

### LPA calibration

We have developed a simple method utilizing a gel imager and MATLAB script to calibrate the outputs of multiple LPA LEDs of the same spectrum to one another (Supplementary Files, Supplementary Methods). To calibrate absolute outputs, or the outputs of LEDs with different spectra, we have developed a method combining a probe spectrophotometer and 3D printed adapter that fits the LPA wells (Supplementary Fig. S20 and Supplementary Methods). Using either method, outputs are adjusted by loading two manually generated text files specifying the grayscale and dot correction values for each LED (Supplementary Methods, examples in Supplementary Files) on to the SD card before running an experiment. Calibration typically reduces LED output variability to <1% (Supplementary Fig. S21).

### Benchmarking the LPA with *E. coli* CcaS-CcaR

**Figure 3:**
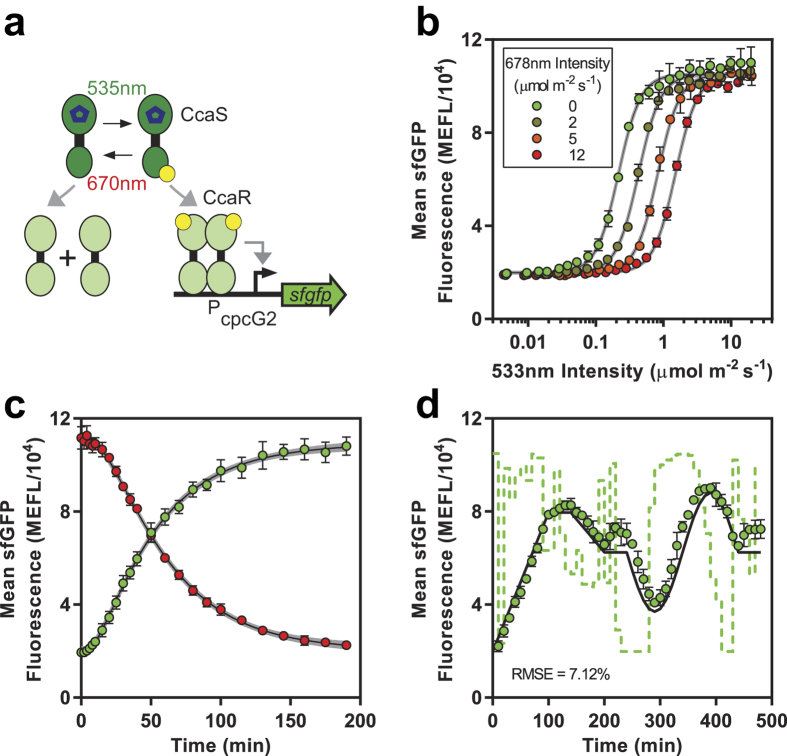
Benchmarking the LPA against *E. coli* CcaS-CcaR. **(a)** CcaS-CcaR system. **(b)** CcaS-CcaR 533 nm light intensity versus sfGFP transfer functions in the presence of increasing 678 nm light. **(c)** Kinetic response to an increase in 533 nm light from 0.00 to 17.88 μmol m^−2^ s^−1^ and simultaneous decrease in 678 nm light from 12.79 μmol m^−2^ s^−1^ to 0.00 μmol m^−2^ s^−1^ (green dots), or decrease in 533 nm light from 17.88 to 0.00 μmol m^−2^ s^−1^ and simultaneous increase in 678 nm from 0.00 μmol m^−2^ s^−1^ to 12.79 μmol m^−2^ s^−1^ (red dots). Black lines represent best fits (Supplementary Table S9 and S10) of our previous CcaS-CcaR mathematical model^45^ to these data. Gray envelopes represent 95% confidence intervals. **(d)** Biological Function Generation. Reference waveform (black line), pre-computed 533 nm light intensity time course (green dashed lines), experimental sfGFP levels (green dots). Constant 12.79 μmol m^−2^ s^−1^ 678 nm was applied. 533 nm intensity values are mapped to sfGFP units through the transfer function in panel b. RMSE between experimental data and reference over three days is shown. Error bars represent the SEM of three experiments over three days.

CcaS-CcaR is a green light activated, red de-activated two component system (TCS) that we previously engineered to control gene expression in *E. coli* (Fig. 3a)^6^. Using the LTA and a superfolder GFP (sfGFP) output, we characterized the relationship between green and red light inputs and transcriptional output dynamics (i.e. I/O)^44^. We developed a predictive mathematical model of the I/O, which we used to pre-compute green light time courses to drive CcaS-CcaR output to follow tailor-made (reference) gene expression signals with high predictability^44^.

We aimed to benchmark the LPA by repeating these CcaS-CcaR experiments. First, we measured the steady state response to green light intensity by outfitting the bottom positions of an LPA with 533 nm (green) LEDs and programming them to emit between 0.00 and 20.10 μmol m^−2^ s^−1^ photons. We grew our previous strain in a shaking LPA under these conditions and measured sfGFP output using flow cytometry (Methods). We observed that sfGFP increases sigmoidally with green intensity with a 5.3-fold dynamic range and Hill parameter (*n*_*H*_) = 2.6 (Fig. 3b), tightly consistent with LTA measurements. The intensity resulting in half maximal output (*k*_*1/2*_) is 0.22 μmol m^−2^ s^−1^, 4.8-fold greater than the LTA value. This discrepancy is likely due to the shorter length between LED and sample in the LPA and small spectrophotometer probe diameter. Because LED emission is conical (Fig. 1c), this length difference concentrates photons on the probe, artificially elevating measured intensity values. This discrepancy could be eliminated using improved probe designs.

Next, we benchmarked the effect of red light by outfitting the bottom and top positions with 533 and 678 nm (red) LEDs and re-measured the green light response in the presence of 2.00–12.00 μmol m^−2^ s^−1^ red photons. We observed that dynamic range and *n*_*H*_ remain unchanged while *k*_1/2_ increases by 0.11 μmol m^−2^ s^−1^ green light per 1.00 μmol m^−2^ s^−1^ red light (Fig. 3b and Supplementary Table S9), consistent with our previous data.

Finally, we used the LPA to characterize the kinetic response of CcaS-CcaR to step changes in green intensity. We observed a 4.5 min delay, an 11 min transcription rate switching time, and sfGFP switching dynamics set by the cell division time (Fig. 3c), equivalent to LTA results. We used these data to re-calibrate the parameters of our model to the LPA conditions (Methods and Supplementary Table S10). Finally, we successfully used the model to program sfGFP expression to follow a challenging waveform comprising linear ramps, a hold, and a sine wave with high predictability (Fig. 3d). We conclude that the LPA meets the same performance standards at the LTA.

### Characterizing CcaS-CcaR forward and reverse action spectra

The action spectrum is the relationship between input wavelength and output activity. While all optogenetic tools have forward (ground state) action spectra (FAS), photoreversible tools also have reverse (activated state) action spectra (RAS).

**Figure 4:**
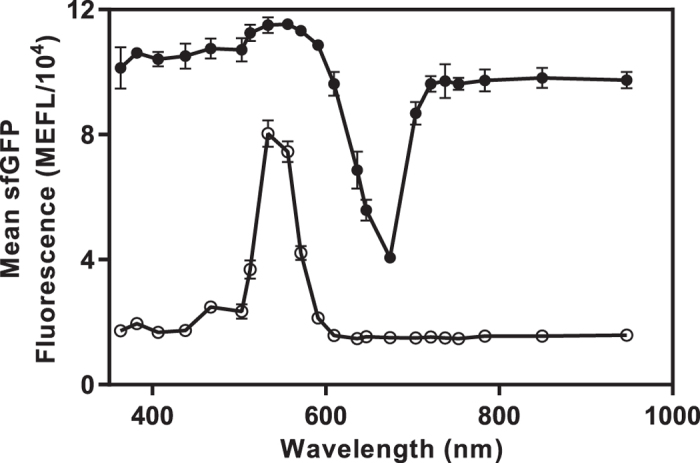
Using the LPA to characterize CcaS-CcaR forward and reverse action spectra. For the FAS (hollow circles), bacteria were exposed to 0.40 μmol m^−2^ s^−1^ photons from a variable wavelength bottom LED (Fig. 1a,c). For the RAS (black circles), bacteria were exposed to 0.40 μmol m^−2^ s^−1^ from the maximally activating 533 nm LED in the top position and 3.21 μmol m^−2^ s^−1^ photons from the variable wavelength bottom LED. Error bars represent the SEM of three experiments over a single day.

We next aimed to demonstrate that the LPA can be used to characterize the FAS and RAS of optogenetic tools using CcaS-CcaR as a model. To this end, we outfitted the bottom position of a device with LEDs of output wavelength between 364 and 947 nm (Fig. 1d and Supplementary Table S5) and programmed each to emit 0.40 μmol m^−2^ s^−1^, or 2*k_1/2_ for the 533 nm LED (Fig. 3b). We then measured the steady state response of CcaS-CcaR to each input as before. This experiment reveals a maximal activating wavelength of 533 nm and >50% maximal activation between 533 and 571 nm (Fig. 4). We previously generated a course-grained map of the CcaS-CcaR FAS using six wavelengths with a rudimentary instrument in an experiment that required approximately one week^6^. By contrast, the LPA enables us to measure a 24 wavelength FAS in a single day.

To characterize the RAS, we outfitted the top positions of a device with the 533 nm LED and the bottom positions with the same LEDs used in the FAS experiment. We set the 533 nm LED to 0.40 μmol m^−2^ s^−1^ and each bottom LED to 3.21 μmol m^−2^ s^−1^ because CcaS-CcaR has approximately 10-fold greater sensitivity to green than red light (Fig. 3b). Consistent with *in vitro* absorbance measurements of activated CcaS^51^, exposure of our CcaS-CcaR expressing strain to these inputs reveals maximal deactivation by the 678 nm LED (Fig. 4). To our knowledge, this is the first measurement of the RAS of an optogenetic tool.

### Characterizing the *S. cerevisiae* CRY2-CIB1 yeast two hybrid system

**Figure 5:**
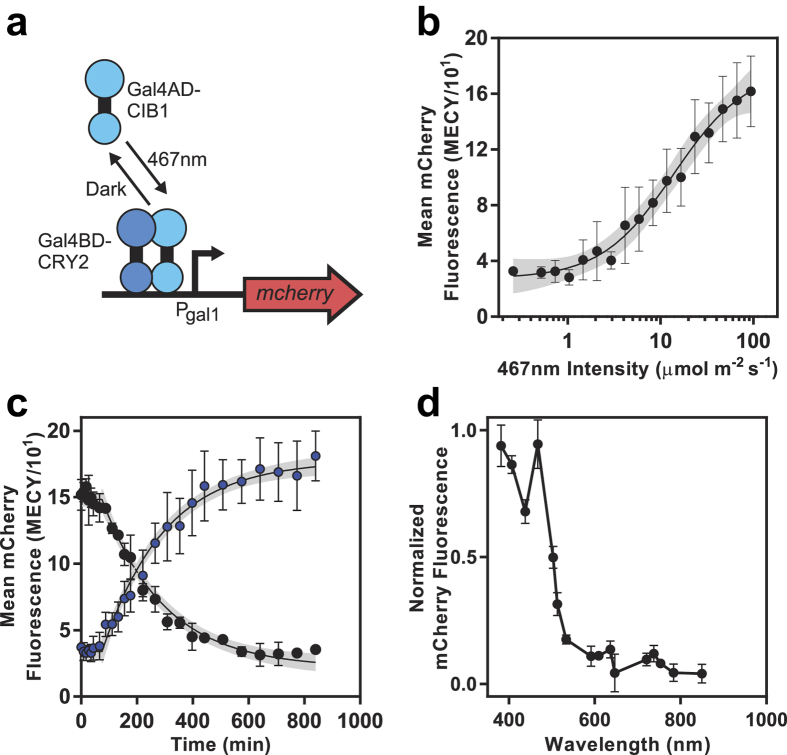
Validating the LPA with yeast by characterizing *S. cerevisiae* CRY2-CIB1 Y2H. **(a)** CRY2-CIB1 Y2H system. **(b)** 467 nm intensity transfer function. The black line represents the best fit to a Hill function. **(c)** Step activation and de-activation kinetics. Cells were either preconditioned for 4 h in the dark and switched to 88.8 μmol m^−2^ s^−1^ 467 nm (blue dots) or preconditioned in 88.8 μmol m^−2^ s^−1^ 467 nm for 10 h and switched to dark (black dots) at time zero. Black lines represent best fits (Supplementary Tables S9 and S11) to a kinetic model (Methods). **(d)** FAS. Experiments were performed as in Fig. 4, but with 88.8 μmol m^−2^ s^−1^ photon flux. Grey envelopes represent 95% confidence interval. Error bars represent the SEM of mCherry levels from three experiments over three days (**b**–**c**) or a single day (**d**).

Tucker and coworkers previously engineered a blue light activated *S. cerevisiae* promoter system by fusing Cryptochrome 2 (CRY2) to the Gal4 DNA binding domain (DBD), and the interaction domain of its blue-light dependent heterodimerizing partner CIB1 to the Gal4 activation domain in a yeast two-hybrid (Y2H) system^11^ (Fig. 5a).

To demonstrate compatibility of the LPA with yeast, we characterized the CRY2-CIB1 Y2H I/O. We outfitted the top and bottom positions of an LPA with 467 nm (blue) LEDs, and programmed them to emit intensities between 0.26 and 1057.00 μmol m^−2^ s^−1^. We grew yeast expressing CRY2-CIB1 Y2H with an mCherry output in a shaking LPA under these conditions for 18 or 24 h and measured output using flow cytometry (Methods). Between 0.26 and 93.50 μmol m^−2^ s^−1^, mCherry increases in a Hill-like manner with 5.6-fold dynamic range, *n*_*H*_ of 1.3 and *k*_*1/2*_ of 10.74 μmol m^−2^ s^−1^ (Fig. 5b), consistent with previous reports^11^,^24^,^52^. Though mCherry increases slightly at higher intensities, we observe growth defects, likely due to heating or phototoxicity (Supplementary Fig. S22).

To characterize CRY2-CIB1 Y2H dynamics, we performed a step increase and decrease in blue light and measured the mCherry response over time (Methods). We observe a delay followed by an exponential increase (step-on) or decrease (step-off) in mCherry to a final steady state (Fig. 5c). By fitting the data to a first order ODE model with a delay (Methods), we quantify the delay to be 75.13 min, and the subsequent time to reach 50% of the final mCherry level to be 222 min (Supplementary Table S11). The slower dynamics compared to CcaS-CcaR are likely due to slower rates of transcriptional activation and cell division, as well as the additional steps of mRNA processing and nuclear/cytoplasmic transport.

Next, we measured the CRY2/CIB1 Y2H FAS by outfitting the bottom position of an LPA with LEDs between 382 and 849 nm and programming their outputs to 88.80 μmol m^−2^ s^−1^, the highest intensity of the dimmest (533 nm) LED. This experiment reveals peak activation in response to the 382 nm and 467 nm LEDs and broad wavelength responsivity in the blue (>50% response from 382 to 503 nm) which matches the broad, multipeaked *in-vitro* absorbance spectrum of CRY2^53^ (Fig. 5d). We conclude that the LPA is compatible with yeast optogenetic tools.

### Spatial control of mammalian cell gene expression with PHYB/VNP-PIF6

**Figure 6:**
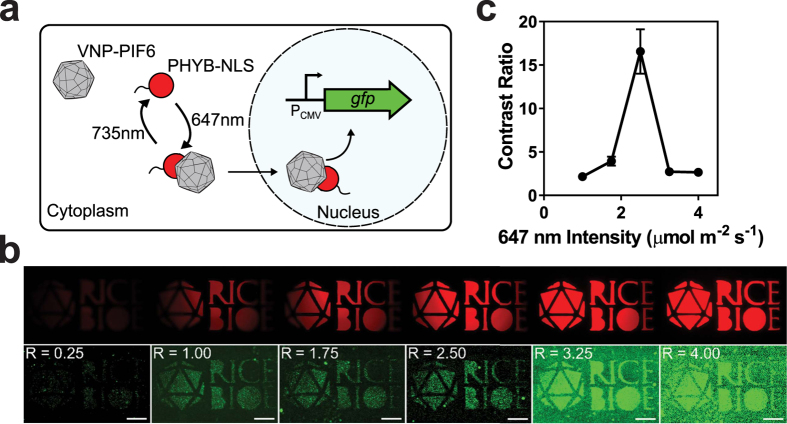
Validating the LPA with mammalian cells by spatial patterning of transgene delivery with PHYB/VNP-PIF6. **(a)** PHYB/VNP-PIF6 system. **(b)** HeLa cells expressing PhyB(908)-NLS were treated with 15μM phycocyanobilin and VNP-PIF6 (1,000 viruses per cell) and exposed through a photomask (top row and Supplementary Fig. S18) to 2.0 μmol m^−2^ s^−1^ 733 nm light for 30 min followed by 1.0 μmol m^−2^ s^−1^ 733 nm light and variable 647 nm light intensities (shown in μmol m^−2^ s^−1^ in upper left of cell fluorescence images) for 60 min. Image corresponding to 647 nm intensity of 1.75 μmol m^−2^ s^−1^ was acquired with photomultiplier setting of 80 while all others were acquired with photomultiplier setting of 60. **(c)** Contrast ratio of cell fluorescence patterns from images in panel b (bottom row). Symbols represent the average ratio of pixel intensity in three light-exposed regions to an unexposed region, while error bars represent the SEM.

We recently modified the surface of viral nanoparticles (VNPs) with Phytochrome Interacting Factor 6 (VNP-PIF6) to bind a heterologously expressed nuclear localization sequence tagged Phytochrome B (PHYB-NLS) and deliver a transgene to the mammalian cell nucleus in a red light activated, far-red deactivated manner^54^ (Fig. 6a). Using an early LPA prototype, we tuned delivery of *gfp* to HeLa cells from 35 to 600% that of wild-type virus. Using custom photomasks, we also patterned GFP expression within different regions of a confluent culture well. Delivery of red light alone resulted in low contrast between cells inside and outside the pattern. Co-delivering far red reduced expression in unwanted areas and increased contrast. However, this prototype had numerous shortcomings including a requirement for a glass bottom plate that often fractured during experiments.

To validate compatibility with mammalian cells and extend our previous results, we outfitted the top and bottom LED positions of an LPA with 647 (red) and 733 nm (far red) LEDs and replaced the plate adapter gasket (Supplementary Fig. S17) with a laser-cut black nitrile photomask (Supplementary Fig. S17). We preconditioned HeLa cells in 6 wells of a single plate with 2.00 μmol m^−2^ s^−1^ far red light for 30 min and then reduced it to 1.00 μmol m^−2^ s^−1^ while introducing variable red light between 0.25 and 4 μmol m^−2^ s^−1^ for 1 h. We allowed GFP to accumulate during a 48 h dark incubation and then imaged the resulting expression patterns by fluorescence microscopy (Methods). As expected, GFP expression increases with red intensity (Fig. 6b). Additionally, the contrast ratio (GFP intensity in exposed divided by that in non-exposed regions) reaches an optimal value of 16.6, near the maximum dynamic range of the system, at an intermediate red:far red ratio of 2.5 (Fig. 6c).

### Using the LPA to entrain the cyanobacterial circadian clock

The cyanobacterium *Synechococcus elongatus* PCC7942 is a model for studying circadian rhythm^55^. Exposing cells to 12 h dark/12 h light cycles entrains the circadian clock. After entrainment, a roughly 24 h transcriptional oscillation^56^,^57^ is maintained for roughly 30–64% of the genome, even under constant light^58^.

**Figure 7:**
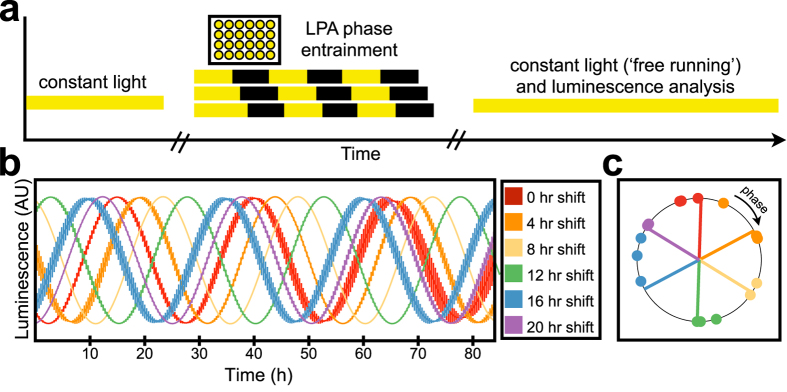
Validating the LPA with cyanobacteria by entraining circadian rhythm in *S. elongatus*. (**a**) Schematic representation of entrainment protocol. Cells grown under constant light were placed in the LPA and entrained with three light-dark cycles, with dark periods starting at different times in order to shift the phases of each well. The cells were then transferred to a plate reader for measurement of luminescence under constant light. (**b**) Luminescence oscillation for cultures entrained starting at different times. Cultures were entrained starting at zero (red), four (orange), eight (yellow), 12 (green), 16 (blue) and 20 (purple) hours. Fits of raw data were normalized to equalize peak height, and the normalized fits were averaged across three replicates except for the cultures in yellow for which only two replicates could be obtained. Bars indicate SEM. (**c**) Phase of cultures entrained starting at different times. Colors are the same as in (**b)**. Dots indicate cosine acrophase (phase of peak) of the replicates. Lines indicate the best fit for the expected locations of acrophase for perfect entrainment.

The time-intensive nature of the entrainment protocol makes it difficult to conduct multiple parallel experiments in order to study clock properties and one group has recently sought to simplify the procedure by using a computer-controlled LED array^48^. To examine whether the LPA can be used to automate circadian entrainment, we outfitted the top and bottom positions of three devices with 647 and 467 nm LEDs to provide a balanced photosynthetic active radiation spectrum typical of grow lights. We programmed 6 different signals across the devices wherein cells were exposed to 3 cycles starting at different times (Fig. 7a). Luminescence from a commonly used reporter system^58^,^59^ wherein luciferase substrate is produced from the “dusk-peaking” promoter *psbAI* and luciferase from the “dawn-peaking” promoter *kaiBC* was then measured. As expected, the oscillations of cultures that were entrained starting at different times showed different phases (Fig. 7b). The order of peaks in luminescence was also as expected, with the cultures entrained starting at 0 h (red) peaking before cultures entrained starting at 4 h (orange), 8 h (yellow), 12 h (green), 16 h (blue) and 20 h (purple) (Fig. 7b,c). However, the position of the peaks exhibits some dispersion, potentially due to heating of the LPA or the transition of cultures from the LPA to the plate reader.

## Discussion

The LPA establishes new standards of flexibility, user-friendliness, and affordability in non-neural optogenetics and photobiology hardware – features that should facilitate widespread adoption. In terms of flexibility, the LPA is compatible with bacteria, yeast, and mammalian cells and other common model organisms. The ~1 mL well volume and the shaker adapters make the device compatible with most model organism growth protocols. The diversity of available LEDs theoretically makes the LPA compatible with all known genetically encoded photoreceptors and most photobiological processes. The second LED position allows improved spatial and dynamical control of photoreversible optogenetic tools and characterization of reverse action spectra. Different spatial patterns can easily be applied by using laser cut gaskets. In addition to these features, the LPA retains the high standards of programmability and experimental precision of the LTA^44^.

The ease of assembly, intuitive Iris interface, simplified liquid handling, and automated calibration script make the LPA user friendly. The LPA can be assembled easily and quickly by a non-expert using fully documented assembly instructions and tutorial videos. Iris converts high-level light function specifications into low level hardware operations, eliminating programming requirements. LPA samples can be loaded and harvested using a multichannel pipettor, and the plate can be sealed and unsealed with an adhesive lid in a single step, facilitating high throughput experimentation. The plate is also compatible with plate readers and plate-based flow cytometers, enabling direct measurements of absorbance, fluorescence, or luminescence without additional liquid handling. Finally, our MATLAB script simplifies calibration for experiments where the same LED is used in multiple wells.

The LPA is more economical than comparable devices (Supplementary Table 12). The 24-well plate costs $7.70 and can be reused dozens of times, lowering per sample cost to <$0.01. Furthermore, the $400 component cost drops to $150 with access to a 3D printer. These low costs make the LPA practical for large scale experiments (Supplementary Fig. S23).

The action spectra of optogenetic tools are seldom measured due to the lack of convenient hardware. Using the LPA, both the FAS and RAS can be measured with 24 wavelength resolution in one experiment. This feature should permit improved characterization of light responsive signaling pathways and enable cross-talk to be minimized when using multiple optogenetic photoreceptors.

Despite early efforts^60^,^61^, there is little data directly comparing the performance features of optogenetic tools. Accordingly, researchers may have difficulty selecting the best tool for a given experiment. On the other hand, the LPA can be used to directly compare optogenetic tools, including those in different organisms. For example, in this study we found that CRY2/CIB1 Y2H is 50 times less sensitive to light than CcaS-CcaR (compare *k*_*1/2*_ in Figs 3b and 5b). Using the basic protocols established here, the LPA could be used to quantitatively compare the spectral, intensity dependent, and dynamical properties of all published optogenetic tools with gene expression outputs in bacteria, yeast, mammalian cells and other organisms, providing much needed information to the community that should accelerate the adoption of optogenetics.

Many circadian assays, such as those measuring the sensitivity of the rhythm to optical perturbations as a function of time of day, are onerous and present a significant barrier to the researcher. The LPA provides a compact and economical solution to this problem. Additionally, the ability to program two wavelengths may ease studies of organisms and processes that respond differently to different wavelengths such as phase resetting in the green algae *C. reinhardtii* in which shifts in the circadian rhythm are wavelength dependent as a function of cellular status. The ability to program 24 light signals simultaneously may also ease experiments such as testing growth of mutants with longer or shorter circadian periods with different lengths of light and dark^62^,^63^,^64^.

By releasing the LPA and Iris with an open-source license, we aim to incorporate other researchers as developers to extend and improve the platform. Similar to a recent example^65^, we have made all design files, a bill of materials, and protocols needed to fabricate, assemble, and operate the device, as well as standard format source code on a public version control system freely available. From this repository, the community can download up-to-date file versions, suggest or make improvements and modifications, design new accessories, and upload all updates for others to use and build upon further. Future designs could incorporate more LEDs per well, lids compatible with gas exchange membranes, sample injection and aspiration, and on-board sensors for measurement of absorbance, fluorescence, or luminescence, enabling optogenetic feedback control^66^. Better heat dissipation for high intensity experiments could be achieved through addition of heat sinks, fans, chassis parts with better ventilation, or even active temperature feedback control. 96-well or greater plate format designs should also be possible. With 3D printing and microcontroller technologies becoming increasingly inexpensive and accessible, such advances can be made by researchers without formal electrical and mechanical engineering backgrounds. We hope that this work not only enables new researchers to perform optogenetics and photobiology experiments, but also motivates them join the growing open-hardware community.

## Methods

### LPA

CAD files, firmware, and detailed documentation on LPA design, fabrication, assembly, calibration, and operation are included in the Supplementary Information and Supplementary Files. The most up-to-date documentation and file versions can be found at http://taborlab.rice.edu/hardware.

### Iris

Source code and detailed descriptions of the staggered start algorithm, waveform handling, file specifications, randomization and de-randomization procedure, and LPF creation using Python are included in the Supplementary Information and Supplementary Files. The most up-to-date documentation and source code can be accessed at http://taborlab.rice.edu/software.

### CcaS-CcaR

All CcaS-CcaR experiments were performed in *E. coli* strain JT2 (RU1012 *Δ*P_*ompC*_-*lacZ*) with previously constructed plasmids pJT119b and pPLPCB(S)^6^. To make frozen starter aliquots, a 3 mL LB + 50 μg/mL kanamycin, 100 μg/mL spectinomycin and 34 μg/mL chloramphenicol culture was inoculated from a −80 °C stock and grown (37 °C, 250 rpm) to OD_600_ <0.3. 30% glycerol was added, absorbance measured, and 50 μL aliquots made and stored at −80 °C. For experiments, M9 + antibiotics was inoculated with a starter aliquot, to OD_600 _= 0.00015. 500 μL of experimental culture was added to each well of the 24-well plate and the plate was sealed with adhesive foil. The plate was placed into the LPA and the assembly mounted on a shaker/incubator at 37 °C, 250 rpm for 8 h. The plate was then removed and chilled in an ice-water bath. Cells were chilled for ≥15 min and the foil removed. 200 μL from each well was transferred to flow cytometry tubes containing 1 mL PBS, and cells were treated with rifampicin as before^44^.

### CRY2-CIB1 Y2H

All CRY2-CIB1 Y2H experiments were performed with the previously described *S. cerevisiae* strain yMM1081 (MAT α, trpΔ63, leu2Δ1, ura3Δ52, gal1ΔmCherry-caURA3) which carries plasmids pGal4AD-CIB1 and pGal4BD-CRY2^11^,^52^. A 3 mL SD (-Ura, -Trp, -Leu) medium starter culture was inoculated from a −80 °C stock and grown (30 °C, 250 r.p.m) overnight to a final density of approximately OD_600_ = 2. The starter culture was diluted into fresh SD to OD_600_ = 0.001. The culture plate was prepared and loaded/unloaded as done with *E. coli*. Cells were grown in the LPA (30 °C, 250 rpm) for 24 h (step-off dynamics experiments) or 18 h (all other experiments). Samples were harvested as described for *E. coli* without rifampicin treatment.

### Flow cytometry

Cytometry was performed with a BD FACScan flow cytometer as previously described^45^. Settings used for each experiment are listed in Supplementary Table S13. Typical count rates of 1,000–2,000 events/s were used and approximately 20,000 events were captured for each sample. Calibration beads (RCP-30-5A, Spherotech) were measured at each session. Data was processed and sfGFP and mCherry fluorescence were calibrated to Molecules of Equivalent Fluorescein (MEFL) and Molecules of Equivalent Cy5 (MECY) with FlowCal^67^.

### CcaS-CcaR model

Data was modeled as previously^44^. Parameter fitting was performed in GraphPad. All parameters were constrained to >0, and *τ*_*delay*_ and *k*_*g*_ are shared between step-on and step-off data sets.

### CRY2-CIB1 Y2H model

We apply a first order linear ODE model


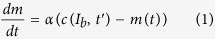



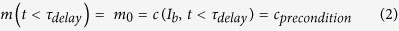


where the rate of change of mCherry, *m*(*t*), is a function of the difference between the mCherry “set-point”, 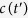 and the current mCherry concentration. 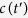 is blue light intensity, *I*_*b*_, mapped to mCherry units through the steady-state transfer function at time 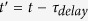. At 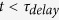, mCherry is equal to its initial value, which is determined by the preconditioning set-point, 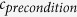. The solution to this ODE has the following piece-wise form:


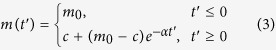


where mCherry remains at its initial value, *m*_0_, for *t* ≤ *τ*_*delay*_, and exponentially increases or decreases with rate constant *α* for *t* ≥ *τ*_*delay*_.

Parameter fitting was performed by nonlinear least squares in GraphPad. All parameters were constrained to >0, and *τ*_*delay*_ and *α* were shared between step-on and step-off data sets.

### PHYB/VNP-PIF6

Experimental protocols were similar to those described previously^54^. Briefly, VNP-PIF6 was isolated by iodixanol gradient centrifugation from HEK293T cells 48 h after a quadruple transfection with pAAV-GFP, pXX6-80, pVP2A-PIF6, and pVP1/3. Patterned gene expression experiments were performed by coating each culture plate well with poly-L-lysine (PLL) and seeding HeLa cells at a density of 1 × 10^5^ per well in DMEM supplemented with 10% fetal bovine serum and 1% penicillin-streptomycin. After 24 h, cells were transfected with PEI-DNA (pKM017)^18^ complexes encoding PhyB908 with a C-terminal NLS fusion. 24 h later, cell media was exchanged for DMEM supplemented with 10% serum, 15 μM phycocyanobilin, and VNP-PIF6 at a multiplicity of infection of 1,000. The culture plate was then loaded into the LPA, placed inside an incubator, and exposed to light time courses described in the results. After 24 h, the culture plate was removed from the LPA and wells were given fresh media without phycocyanobilin or VNP-PIF6. Cells were incubated for an additional 24 h in the dark to allow accumulation of GFP, and were then treated with 4% paraformaldehyde in PBS and imaged. All incubation steps were performed at 37 °C, 5% CO_2_, and without shaking. All manipulations after addition of PCB were performed under a green safelight. Three diffuser sheets (#3008, Rosco) were used in place of the LED spacer gasket and adhesive foil was omitted to avoid light reflections interfering with the pattern. Though we observed no changes in cell or media appearance, the LPA plate lid likely reduced or prevented gas exchange during the 24 h cells spent inside the device.

### Microscopy

Imaging was performed with a Nikon A1 microscope with 2X objective and 488 nm sample illumination. 4 × 4 arrays of images for each pattern condition were stitched together and given pseudocolor using NIS Elements Software (Nikon). Images shown are cropped to the border of the pattern area.

### Cyanobacterial Circadian Rhythm

All circadian rhythm experiments were performed with *S. elongatus* PCC 7942 strain AMC462^68^. 1.5 ml of exponential phase culture diluted to OD_750_ 0.05 was pipetted into each well of the 24-well culture plate. Extra distilled water was pipetted between wells to reduce evaporation, and the plate was covered with a transparent lid (Visiplate 24-TC Part: 1450-6045) with adhesive foil (F96VWR100). The cells were then grown in the LPA at 30 °C with shaking at 255 rpm for 4 days. Photon flux of 647 and 467 nm LEDs were 68.86 and 244.76 μmol m^−2^ s^−1^, respectively.

### Luminescence data acquisition and analysis

Luminescence of 150 μl of culture in a 96-well black with clear bottom plate with transparent lid was measured using a Tecan M200 with a luminescence module and an integration time of 1000 ms. Between readings, the plate was moved out of the machine for 20 min and kept under a fluorescent lamp at 4500 lux. Readings were taken for a total of 160 h.

Luminesce data was fitted with BRASS (Biological Rhythms Analysis Software System; A. J. Millar laboratory, University of Edinburgh, Scotland, United Kingdom) and FFT-NLLS (Fast Fourier Transform-Nonlinear Least Squares)^69^,^70^. At least 72 h of each culture was included in the analysis.

The best fits for the expected phases were calculated by minimizing the root mean square distance from the location of the expected phase to the location of experimental phase using the GRG Nonlinear solving method in Excel.

### Iris session files

Iris session (savefile.irs) files for each experiment are available in the Supplementary Files.

### Strain and Plasmid Accession Information

Accession information for strains and plasmids used in this study are available in Supplementary Tables S14 and S15.

## Additional Information

**How to cite this article**: Gerhardt, K. P. *et al.* An open-hardware platform for optogenetics and photobiology. *Sci. Rep.* **6**, 35363; doi: 10.1038/srep35363 (2016).

## Supplementary Material

Supplementary Information

Supplementary Files

Supplementary Video 1

Supplementary Video 2

Supplementary Video 3

Supplementary Video 4

Supplementary Video 5

## References

[b1] Boyden E. S., Zhang F., Bamberg E., Nagel G. & Deisseroth K. Millisecond-timescale, genetically targeted optical control of neural activity. Nat. Neurosci. 8, 1263–1268 (2005). 16116447 10.1038/nn1525

[b2] Deisseroth K. Optogenetics: 10 years of microbial opsins in neuroscience. Nat. Neurosci. 18, 1213–1225 (2015). 26308982 10.1038/nn.4091 PMC4790845

[b3] Shimizu-Sato S., Huq E., Tepperman J. M. & Quail P. H. A light-switchable gene promoter system. Nat. Biotechnol. 20, 1041–1044 (2002). 12219076 10.1038/nbt734

[b4] Levskaya A. *et al.* Synthetic biology: engineering Escherichia coli to see light. Nature 438, 441–442 (2005). 16306980 10.1038/nature04405

[b5] Möglich A., Ayers R. A. & Moffat K. Design and signaling mechanism of light-regulated histidine kinases. J. Mol. Biol. 385, 1433–1444 (2009). 19109976 10.1016/j.jmb.2008.12.017 PMC3527124

[b6] Tabor J. J., Levskaya A. & Voigt C. A. Multichromatic control of gene expression in Escherichia coli. J. Mol. Biol. 405, 315–324 (2011). 21035461 10.1016/j.jmb.2010.10.038 PMC3053042

[b7] Ohlendorf R., Vidavski R. R., Eldar A., Moffat K. & Möglich A. From dusk till dawn: one-plasmid systems for light-regulated gene expression. J. Mol. Biol. 416, 534–542 (2012). 22245580 10.1016/j.jmb.2012.01.001

[b8] Schmidl S. R., Sheth R. U., Wu A. & Tabor J. J. Refactoring and optimization of light-switchable Escherichia coli two-component systems. ACS Synth. Biol. 3, 820–831 (2014). 25250630 10.1021/sb500273n

[b9] Ryu M.-H. & Gomelsky M. Near-infrared light responsive synthetic c-di-GMP module for optogenetic applications. ACS Synth. Biol. 3, 802–810 (2014). 24926804 10.1021/sb400182x PMC4277780

[b10] Abe K. *et al.* Engineering of a green-light inducible gene expression system in Synechocystis sp. PCC6803. Microb. Biotechnol. 7, 177–183 (2014). 24330635 10.1111/1751-7915.12098 PMC3937721

[b11] Kennedy M. J. *et al.* Rapid blue-light-mediated induction of protein interactions in living cells. Nat. Methods 7, 973–975 (2010). 21037589 10.1038/nmeth.1524 PMC3059133

[b12] Rizzini L. *et al.* Perception of UV-B by the Arabidopsis UVR8 protein. Science 332, 103–106 (2011). 10.1126/science.1200660 21454788

[b13] Ye H., Daoud-El Baba M., Peng R.-W. & Fussenegger M. A synthetic optogenetic transcription device enhances blood-glucose homeostasis in mice. Science 332, 1565–1568 (2011). 21700876 10.1126/science.1203535

[b14] Polstein L. R. & Gersbach C. A. Light-inducible spatiotemporal control of gene activation by customizable zinc finger transcription factors. J. Am. Chem. Soc. 134, 16480–16483 (2012). 22963237 10.1021/ja3065667 PMC3468123

[b15] Wang X., Chen X. & Yang Y. Spatiotemporal control of gene expression by a light-switchable transgene system. Nat. Methods 9, 266–269 (2012). 22327833 10.1038/nmeth.1892

[b16] Crefcoeur R. P., Yin R., Ulm R. & Halazonetis T. D. Ultraviolet-B-mediated induction of protein-protein interactions in mammalian cells. Nat. Commun. 4, 1779 (2013). 23653191 10.1038/ncomms2800

[b17] Konermann S. *et al.* Optical control of mammalian endogenous transcription and epigenetic states. Nature 500, 472–476 (2013). 23877069 10.1038/nature12466 PMC3856241

[b18] Müller K. *et al.* A red/far-red light-responsive bi-stable toggle switch to control gene expression in mammalian cells. Nucleic Acids Res. 41, e77 (2013). 23355611 10.1093/nar/gkt002 PMC3627562

[b19] Motta-Mena L. B. *et al.* An optogenetic gene expression system with rapid activation and deactivation kinetics. Nat. Chem. Biol. 10, 196–202 (2014). 24413462 10.1038/nchembio.1430 PMC3944926

[b20] Müller K., Engesser R., Timmer J., Zurbriggen M. D. & Weber W. Orthogonal optogenetic triple-gene control in Mammalian cells. ACS Synth. Biol. 3, 796–801 (2014). 25343333 10.1021/sb500305v

[b21] Nihongaki Y., Yamamoto S., Kawano F., Suzuki H. & Sato M. CRISPR-Cas9-based photoactivatable transcription system. Chem. Biol. 22, 169–174 (2015). 25619936 10.1016/j.chembiol.2014.12.011

[b22] Polstein L. R. & Gersbach C. A. A light-inducible CRISPR-Cas9 system for control of endogenous gene activation. Nat. Chem. Biol. 11, 198–200 (2015). 25664691 10.1038/nchembio.1753 PMC4412021

[b23] Chan Y.-B., Alekseyenko O. V. & Kravitz E. A. Optogenetic Control of Gene Expression in Drosophila. PLoS One 10, e0138181 (2015). 26383635 10.1371/journal.pone.0138181 PMC4575133

[b24] Liu H., Gomez G., Lin S., Lin S. & Lin C. Optogenetic control of transcription in zebrafish. PLoS One 7, e50738 (2012). 23226369 10.1371/journal.pone.0050738 PMC3511356

[b25] Müller K. *et al.* A red light-controlled synthetic gene expression switch for plant systems. Mol. Biosyst. 10, 1679–1688 (2014). 24469598 10.1039/c3mb70579j

[b26] Cao J. *et al.* Light-inducible activation of target mRNA translation in mammalian cells. Chem. Commun. (Camb). 49, 8338–8340 (2013). 23925486 10.1039/c3cc44866e

[b27] Walsh S., Gardner L., Deiters A. & Williams G. J. Intracellular light-activation of riboswitch activity. Chembiochem 15, 1346–1351 (2014). 24861567 10.1002/cbic.201400024 PMC4095863

[b28] Renicke C., Schuster D., Usherenko S., Essen L.-O. & Taxis C. A LOV2 domain-based optogenetic tool to control protein degradation and cellular function. Chem. Biol. 20, 619–626 (2013). 23601651 10.1016/j.chembiol.2013.03.005

[b29] Levskaya A., Weiner O. D., Lim W. A. & Voigt C. A. Spatiotemporal control of cell signalling using a light-switchable protein interaction. Nature 461, 997–1001 (2009). 19749742 10.1038/nature08446 PMC2989900

[b30] Dueber J. E. *et al.* Synthetic protein scaffolds provide modular control over metabolic flux. Nat. Biotechnol. 27, 753–759 (2009). 19648908 10.1038/nbt.1557

[b31] Gautier A., Deiters A. & Chin J. W. Light-activated kinases enable temporal dissection of signaling networks in living cells. J. Am. Chem. Soc. 133, 2124–7 (2011). 21271704 10.1021/ja1109979 PMC3048767

[b32] Toettcher J. E., Gong D., Lim W. A. & Weiner O. D. Light-based feedback for controlling intracellular signaling dynamics. Nat. Methods 8, 837–839 (2011). 21909100 10.1038/nmeth.1700 PMC3184382

[b33] Bugaj L. J., Choksi A. T., Mesuda C. K., Kane R. S. & Schaffer D. V. Optogenetic protein clustering and signaling activation in mammalian cells. Nat. Methods 10, 249–252 (2013). 23377377 10.1038/nmeth.2360

[b34] Toettcher J. E., Weiner O. D. & Lim W. A. Using optogenetics to interrogate the dynamic control of signal transmission by the Ras/Erk module. Cell 155, 1422–1434 (2013). 24315106 10.1016/j.cell.2013.11.004 PMC3925772

[b35] Wend S. *et al.* Optogenetic control of protein kinase activity in mammalian cells. ACS Synth. Biol. 3, 280–5 (2014). 24090449 10.1021/sb400090s

[b36] Bugaj L. J. *et al.* Regulation of endogenous transmembrane receptors through optogenetic Cry2 clustering. Nat. Commun. 6, 6898 (2015). 25902152 10.1038/ncomms7898 PMC4408875

[b37] Chen D., Gibson E. S. & Kennedy M. J. A light-triggered protein secretion system. J. Cell Biol. 201, 631–640 (2013). 23671313 10.1083/jcb.201210119 PMC3653365

[b38] Niopek D. *et al.* Engineering light-inducible nuclear localization signals for precise spatiotemporal control of protein dynamics in living cells. Nat. Commun. 5, 4404 (2014). 25019686 10.1038/ncomms5404 PMC4104460

[b39] Beyer H. M. *et al.* Red Light-Regulated Reversible Nuclear Localization of Proteins in Mammalian Cells and Zebrafish. ACS Synth. Biol. 4, 951–958 (2015). 25803699 10.1021/acssynbio.5b00004

[b40] Yumerefendi H. *et al.* Control of Protein Activity and Cell Fate Specification via Light-Mediated Nuclear Translocation. PLoS One 10, e0128443 (2015). 26083500 10.1371/journal.pone.0128443 PMC4471001

[b41] Niopek D., Wehler P., Roensch J., Eils R. & Di Ventura B. Optogenetic control of nuclear protein export. Nat. Commun. 7, 10624 (2016). 26853913 10.1038/ncomms10624 PMC4748110

[b42] Nihongaki Y., Kawano F., Nakajima T. & Sato M. Photoactivatable CRISPR-Cas9 for optogenetic genome editing. Nat. Biotechnol. 33, 755–760 (2015). 26076431 10.1038/nbt.3245

[b43] Hemphill J., Borchardt E. K., Brown K., Asokan A. & Deiters A. Optical Control of CRISPR/Cas9 Gene Editing. J. Am. Chem. Soc. 137, 5642–5645 (2015). 25905628 10.1021/ja512664v PMC4919123

[b44] Olson E. J., Hartsough L. A., Landry B. P., Shroff R. & Tabor J. J. Characterizing bacterial gene circuit dynamics with optically programmed gene expression signals. Nat. Methods 11, 449–455 (2014). 24608181 10.1038/nmeth.2884

[b45] Richter F. *et al.* Upgrading a microplate reader for photobiology and all-optical experiments. Photochem. Photobiol. Sci. 14, 270–279 (2015). 25373866 10.1039/c4pp00361f

[b46] Davidson E. A., Basu A. S. & Bayer T. S. Programming microbes using pulse width modulation of optical signals. J. Mol. Biol. 425, 4161–4166 (2013). 23928560 10.1016/j.jmb.2013.07.036

[b47] Lee J. M., Lee J., Kim T. & Lee S. K. Switchable gene expression in Escherichia coli using a miniaturized photobioreactor. PLoS One 8, e52382 (2013). 23349683 10.1371/journal.pone.0052382 PMC3547951

[b48] Pattanayak G. K., Phong C. & Rust M. J. Rhythms in energy storage control the ability of the cyanobacterial circadian clock to reset. Curr. Biol. 24, 1934–1938 (2014). 25127221 10.1016/j.cub.2014.07.022 PMC4477845

[b49] Müller K., Zurbriggen M. D. & Weber W. Control of gene expression using a red- and far-red light-responsive bi-stable toggle switch. Nat. Protoc. 9, 622–632 (2014). 24556785 10.1038/nprot.2014.038

[b50] Hannanta-anan P. *et al.* Optogenetic Control of Calcium Oscillation Waveform Defines NFAT as an Integrator of Calcium Load. Cell Syst. 2, 283–288 (2016). 27135540 10.1016/j.cels.2016.03.010 PMC4909256

[b51] Hirose Y., Shimada T., Narikawa R., Katayama M. & Ikeuchi M. Cyanobacteriochrome CcaS is the green light receptor that induces the expression of phycobilisome linker protein. Proc. Natl. Acad. Sci. USA 105, 9528–9533 (2008). 18621684 10.1073/pnas.0801826105 PMC2474522

[b52] Melendez J. *et al.* Real-time optogenetic control of intracellular protein concentration in microbial cell cultures. Integr. Biol. (Camb). 6, 366–372 (2014). 24477515 10.1039/c3ib40102b

[b53] Li X. *et al.* Arabidopsis cryptochrome 2 (CRY2) functions by the photoactivation mechanism distinct from the tryptophan (trp) triad-dependent photoreduction. Proc. Natl. Acad. Sci. USA 108, 20844–20849 (2011). 22139370 10.1073/pnas.1114579108 PMC3251054

[b54] Gomez E. J., Gerhardt K., Judd J., Tabor J. J. & Suh J. Light-Activated Nuclear Translocation of Adeno-Associated Virus Nanoparticles Using Phytochrome B for Enhanced, Tunable, and Spatially Programmable Gene Delivery. ACS Nano acsnano.5b05558 doi: 10.1021/acsnano.5b05558 (2015). 26618393

[b55] Golden S. S., Ishiura M., Johnson C. H. & Kondo T. Cyanobacterial Circadian Rhythms. Annu. Rev. Plant Physiol. Plant Mol. Biol. 48, 327–354 (1997). 15012266 10.1146/annurev.arplant.48.1.327

[b56] Ito H. *et al.* Cyanobacterial daily life with Kai-based circadian and diurnal genome-wide transcriptional control in Synechococcus elongatus. Proc. Natl. Acad. Sci. USA 106, 14168–14173 (2009). 19666549 10.1073/pnas.0902587106 PMC2729038

[b57] Vijayan V., Zuzow R. & O’Shea E. K. Oscillations in supercoiling drive circadian gene expression in cyanobacteria. Proc. Natl. Acad. Sci. USA 106, 22564–22568 (2009). 20018699 10.1073/pnas.0912673106 PMC2799730

[b58] Kondo T. *et al.* Circadian rhythms in prokaryotes: luciferase as a reporter of circadian gene expression in cyanobacteria. Proc. Natl. Acad. Sci. USA 90, 5672–5676 (1993). 8516317 10.1073/pnas.90.12.5672 PMC46783

[b59] Katayama M., Tsinoremas N. F., Kondo T. & Golden S. S. cpmA, a gene involved in an output pathway of the cyanobacterial circadian system. J. Bacteriol. 181, 3516–3524 (1999). 10348865 10.1128/jb.181.11.3516-3524.1999 PMC93820

[b60] Gautier A. *et al.* How to control proteins with light in living systems. Nat. Chem. Biol. 10, 533–541 (2014). 24937071 10.1038/nchembio.1534

[b61] Pathak G. P., Strickland D., Vrana J. D. & Tucker C. L. Benchmarking of optical dimerizer systems. ACS Synth. Biol. 3, 832–838 (2014). 25350266 10.1021/sb500291r PMC4277767

[b62] Johnson C. H., Kondo T. & Hastings J. W. Action Spectrum for Resetting the Circadian Phototaxis Rhythm in the CW15 Strain of Chlamydomonas: II. Illuminated Cells. Plant Physiol. 97, 1122–1129 (1991). 16668498 10.1104/pp.97.3.1122 PMC1081131

[b63] Kondo T., Johnson C. H. & Hastings J. W. Action Spectrum for Resetting the Circadian Phototaxis Rhythm in the CW15 Strain of Chlamydomonas: I. Cells in Darkness. Plant Physiol. 95, 197–205 (1991). 16667951 10.1104/pp.95.1.197 PMC1077506

[b64] Ma P., Woelfle M. A. & Johnson C. H. An Evolutionary Fitness Enhancement Conferred by the Circadian System in Cyanobacteria. Chaos. Solitons. Fractals 50, 65–74 (2013). 23626410 10.1016/j.chaos.2012.11.006 PMC3633149

[b65] Takahashi C. N., Miller A. W., Ekness F., Dunham M. J. & Klavins E. A low cost, customizable turbidostat for use in synthetic circuit characterization. ACS Synth. Biol. 4, 32–38 (2015). 25036317 10.1021/sb500165g PMC4304434

[b66] Milias-Argeitis A. *et al.* In silico feedback for *in vivo* regulation of a gene expression circuit. Nat. Biotechnol. 29, 1114–1116 (2011). 22057053 10.1038/nbt.2018 PMC4565053

[b67] Castillo-Hair S. M. *et al.* FlowCal: A user-friendly, open source software tool for automatically converting flow cytometry data from arbitrary to calibrated units. ACS Synth. Biol, doi: 10.1021/acssynbio.5b00284 (2016). PMC5556937 27110723

[b68] Mackey S. R., Ditty J. L., Clerico E. M. & Golden S. S. Detection of rhythmic bioluminescence from luciferase reporters in cyanobacteria. Methods Mol. Biol. 362, 115–129 (2007). 17417005 10.1007/978-1-59745-257-1_8

[b69] Plautz J. D. *et al.* Quantitative analysis of Drosophila period gene transcription in living animals. J. Biol. Rhythms 12, 204–217 (1997). 9181432 10.1177/074873049701200302

[b70] Straume M., Frasier-Cadoret S. G. & Johnson M. L. Topics in Fluorescence Spectroscopy 2, (Kluwer Academic Publishers, 2002).

