# Supplementary material for: An open-hardware platform for optogenetics and photobiology
Source: Sci Rep. 2016 Nov 2;6:35363. doi: 10.1038/srep35363 (PMC5096413; doi:10.1038/srep35363)
Supplement: Supplementary Information [file srep35363-s1.pdf]

## Supplementary Information for

# An open-hardware platform for optogenetics and photobiology

Karl P. Gerhardt, Evan J. Olson, Sebastian M. Castillo-Hair, Lucas A. Hartsough, Brian P. Landry, Felix Ekness, Rayka Yokoo, Eric J. Gomez, Prabha Ramakrishnan, Junghae Suh, David F. Savage, Jeffrey J. Tabor.

## Table of Contents

### Supplementary Figures

**Supplementary Figure S1.** Detailed circuit board photographs.................................... 4

**Supplementary Figure S2.** Circuit board SD-card reader stage schematic.................. 5

**Supplementary Figure S3.** Circuit board microcontroller stage schematic.................... 6

**Supplementary Figure S4.** Circuit board LED driver stage schematic .......................... 7

**Supplementary Figure S5.** Circuit board power stage schematic ................................. 8

**Supplementary Figure S6.** LPA power connections.................................................... 9

**Supplementary Figure S7.** Photographs of 3D printed auxiliary parts ........................ 10

**Supplementary Figure S8.** Atmel Studio, linker libraries path.................................... 12

**Supplementary Figure S9.** Atmel Studio, compiler miscellaneous options ................. 13

**Supplementary Figure S10.** Atmel Studio, compiler’s included directories ................. 14

**Supplementary Figure S11.** Atmel Studio, missing referenced file ............................ 15

**Supplementary Figure S12.** The firmware project with the config.h file open ............. 16

**Supplementary Figure S13.** The Device Programming dialog, after connecting to the programmer................................................................................................................ 17

**Supplementary Figure S14.** State transition diagram of the LPA firmware ................. 18

**Supplementary Figure S15.** Close-up photograph of LED and LED socket friction fit 19

**Supplementary Figure S16.** Detailed photographs of 3D printed LPA parts ............... 20

**Supplementary Figure S17.** Photograph and schematic of culture plate used in the LPA ............................................................................................................................ 22

**Supplementary Figure S18.** Photographs of LPA gaskets......................................... 23

**Supplementary Figure S19.** Visualization of staggered-start waveform segmentation for each dynamic waveform .......... 24

**Supplementary Figure S20.** Photograph of light intensity probe measurement setup 26

**Supplementary Figure S21.** LED outputs before and after image analysis calibration 27

**Supplementary Figure S22.** Growth rate dependence of S. cerevisiae yMM1081 on 467nm light intensity.......... 28

**Supplementary Figure S23.** Scalability of LPAs .......... 29

**Supplementary Tables**

**Supplementary Table S1.** Circuit board component parts list .......... 30

**Supplementary Table S2.** Equipment required for circuit board soldering procedure . 31

**Supplementary Table S3.** Error conditions, as indicated by status LEDs.......... 32

**Supplementary Table S4.** States of the LPA firmware .......... 33

**Supplementary Table S5.** LEDs used in this study and other LED examples .......... 34

**Supplementary Table S6.** Non-electrical LPA components.......... 36

**Supplementary Table S7.** Iris preconditioning for each light input function .......... 37

**Supplementary Table S8.** LPF file specifications .......... 38

**Supplementary Table S9** Best fit parameters and standard errors for CcaS-CcaR steady-state transfer function .......... 39

**Supplementary Table S10.** Best fit parameters and standard errors for CcaS-CcaR kinetic model .......... 40

**Supplementary Table S11.** Best fit parameters and standard errors for CRY2-CIB1 Y2H steady-state intensity transfer function and kinetic model .......... 41

**Supplementary Table S12.** Comparison of select non-neural optogenetic hardware from the literature .......... 42

**Supplementary Table S13.** Flow cytometry settings .......... 44

**Supplementary Table S14.** Strains used in this study and their contact laboratories.. 45

**Supplementary Table S15.** Plasmids used in this study and their accession information .......... 46

**Supplementary Methods**

Circuit board fabrication and assembly .......... 47
Firmware Programming .......... 50
Firmware compiling .......... 52
LED Installation and reconfiguration .......... 57
Laser cutting gaskets .......... 58
3D printing LPA parts .......... 59
LPA final assembly .......... 60
Calibrating LEDs .......... 62

**Supplementary Notes**

LPA firmware description .......... 64
Running Iris offline .......... 67
Iris waveform handling .......... 68
Iris randomization and de-randomization procedure .......... 71
Writing an LPF using Python .......... 72

**Supplementary Video Legends**

**Supplementary Video Legend S1.** LED socket alignment and soldering .......... 73
**Supplementary Video Legend S2.** Assembling and powering the LPA .......... 74
**Supplementary Video Legend S3.** Iris steady-state mode .......... 75
**Supplementary Video Legend S4.** Iris dynamics mode .......... 76
**Supplementary Video Legend S5.** Iris advanced mode .......... 77

**Supplementary References** .......... 78

## Supplementary Figures

**Supplementary Figure S1.** Detailed circuit board photographs.

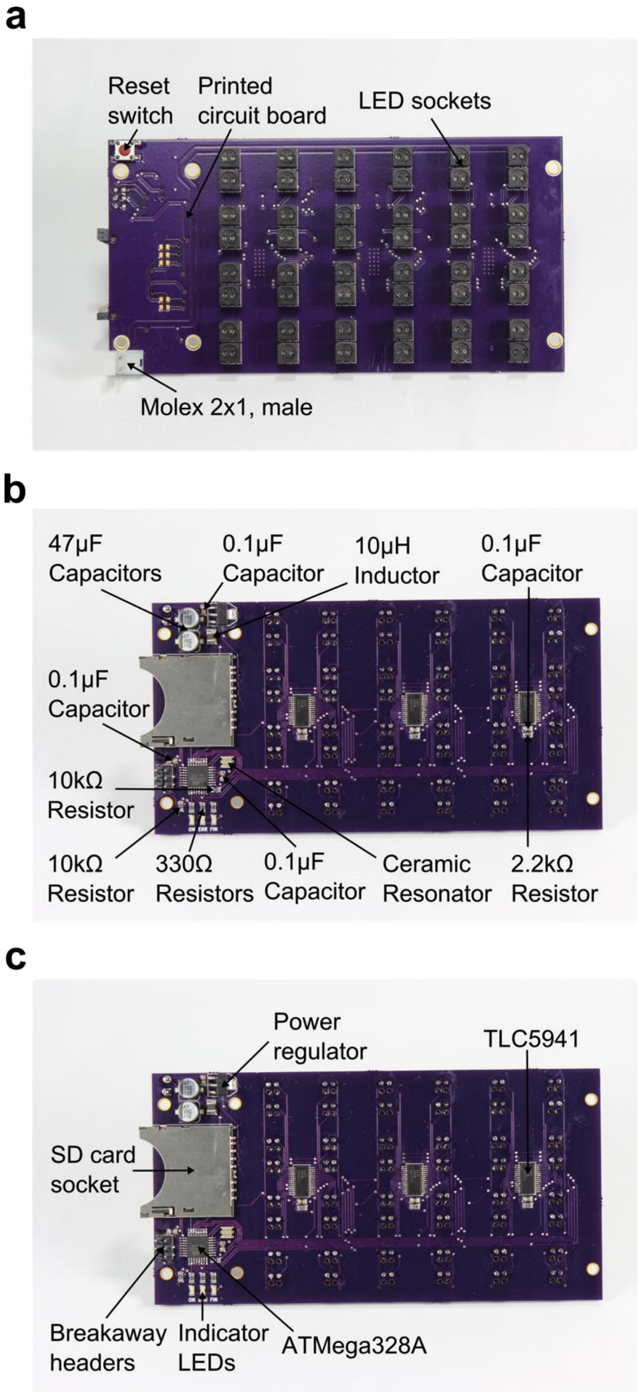


Detailed photographs of the LPA circuit board populated with electrical components from **Supplementary Table S1**. Panels show (**a**) top-down and (**b**-**c**) bottom-up perspectives. The method for soldering circuit board components can be found in the **Supplementary Method** on circuit board fabrication and assembly. The significance of each circuit board component is described in **Supplementary Figs. S2-S5**.

**Supplementary Figure S2.** Circuit board SD-card reader stage schematic.

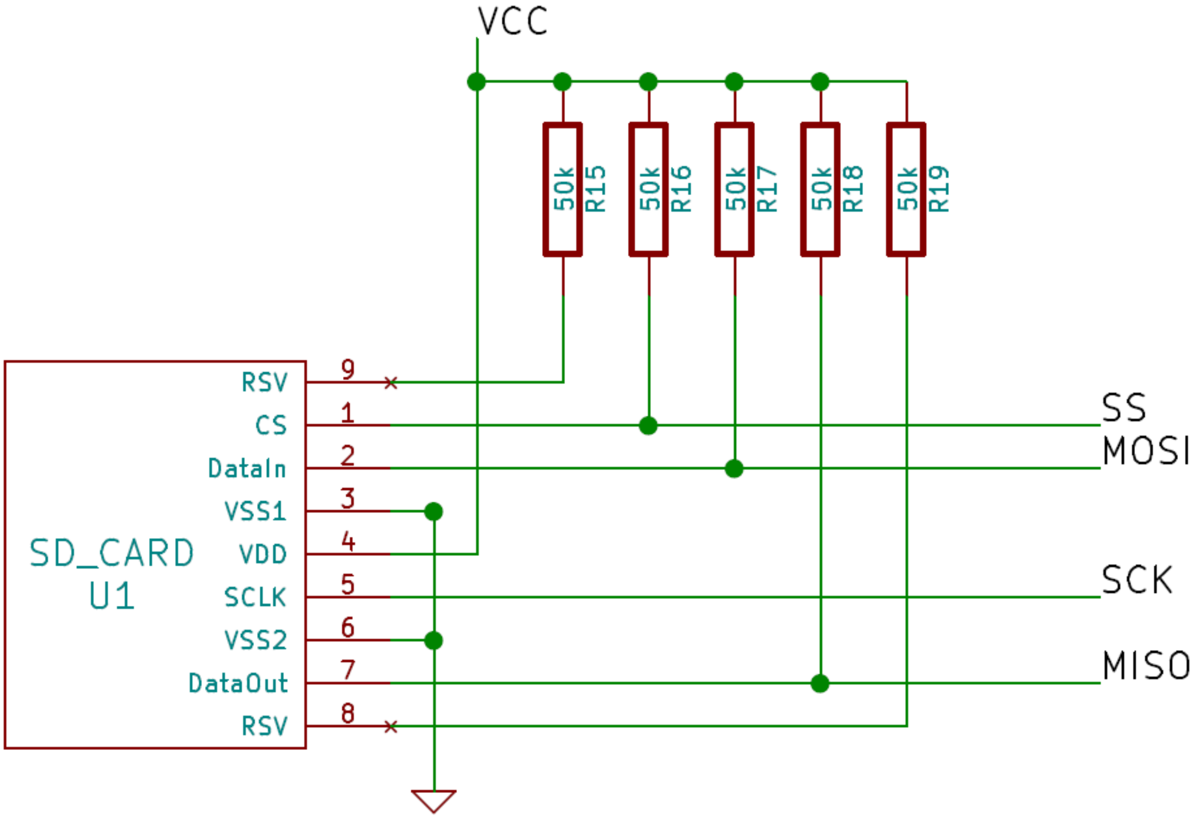


The SD card reader stage contains the hardware needed for the microcontroller to properly read an SD card. This stage includes the SD card socket (U1), a series of 50kΩ pull-up resistors (R15-R19), and the connections to the microcontroller (SS, MOSI, SCK, MISO), regulated power line (VCC) and ground. The SD card is operated in Serial Peripheral Interface (SPI) Mode, and has been wired to the microcontroller accordingly[1,2], with lines SS, MOSI, SCK, and MISO going to pins PB2 (14), PB3 (15), PB5 (16) and PB4 (17), respectively. If the internal pull-ups of the microcontroller are used, resistors R15 through R19 are not needed.

**Supplementary Figure S3.** Circuit board microcontroller stage schematic.

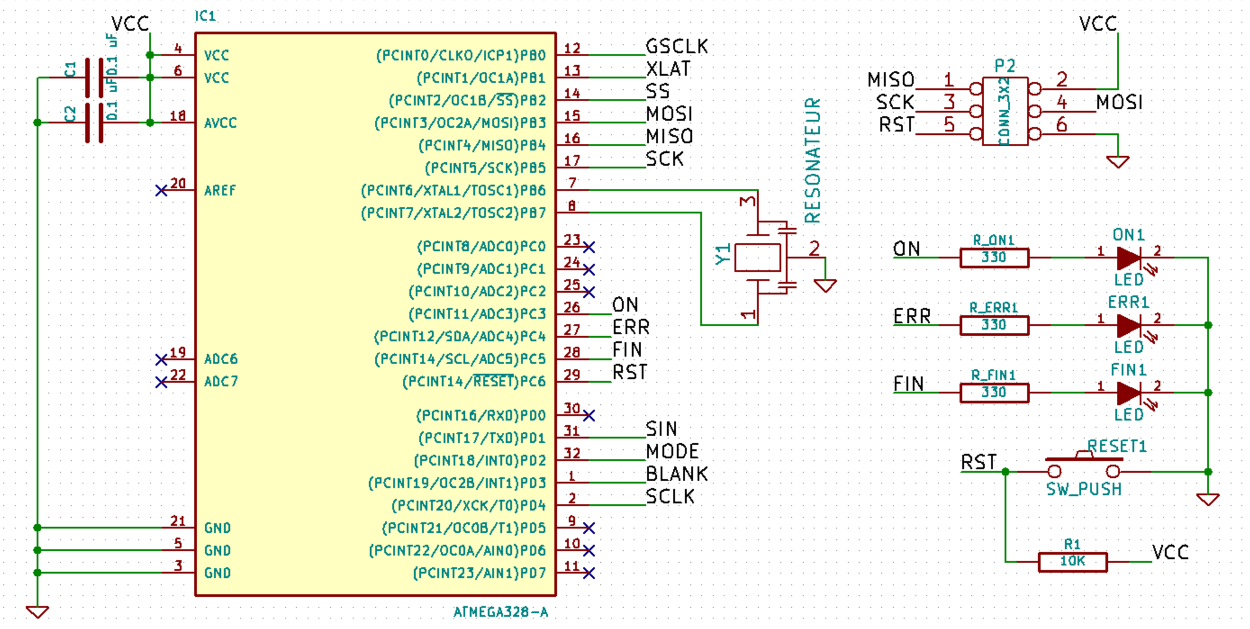


The microcontroller stage contains the microcontroller, programming pins, reset button, and status LEDs. The microcontroller is an Atmel ATMEGA328[1], which receives its power from the regulated power line (VCC). A pair of decoupling capacitors have been included in the design (C1, C2). A 16Mhz ceramic resonator with built-in capacitors (Y1) is needed for the microcontroller to function at the appropriate frequency. The programming pins (P2) have been wired according to the ISP specifications[3], and they are compatible with popular Atmel programmers such as the AVRISP mkII and the AVR Dragon. The reset circuit is comprised of button RESET1 and resistor R1. A set of status LEDs (ON, ERR, FIN) with their respective 330Ω current-limiting resistors (R_ON1, R_ERR1, R_FIN1) have been included to read the status of the device while running.

**Supplementary Figure S4.** Circuit board LED driver stage schematic.

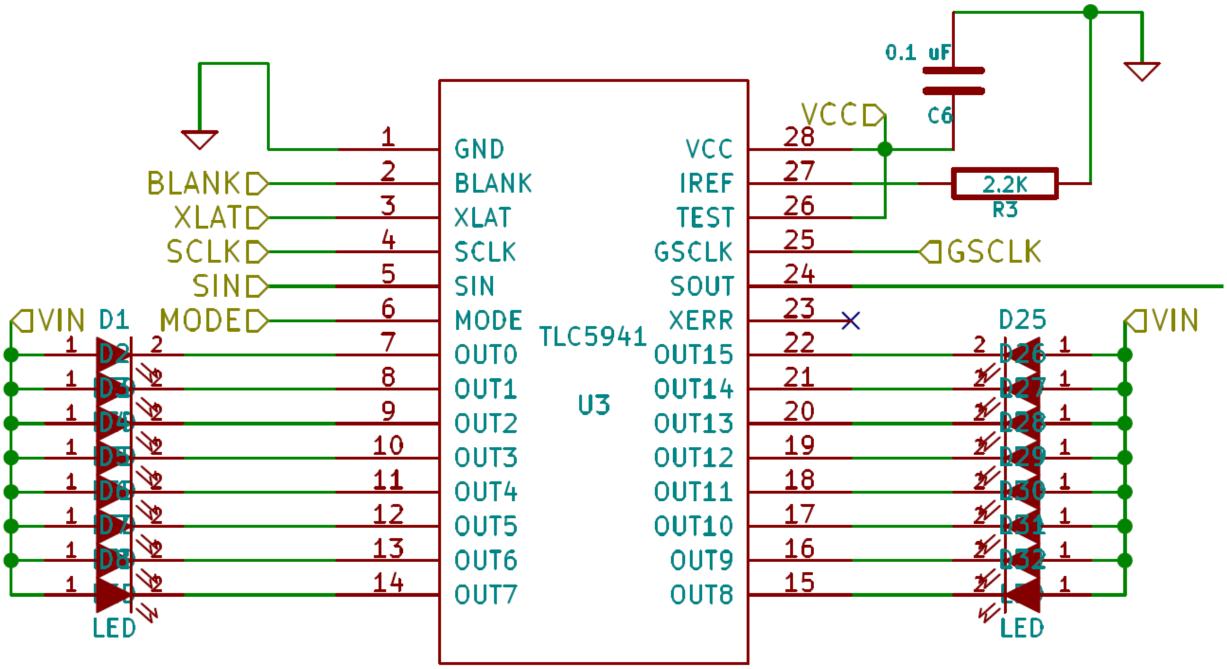


The LED driver stage contains the hardware needed for controlling the intensity of the LEDs in response to instructions from the microcontroller, and the LEDs themselves. The LED Driver Stage is comprised of LED Drivers TLC5941[4] connected in series. Each TLC5941 is able to control 16 LEDs. Therefore, 3 are used in the 24-well LPA which contains 2 LEDs per well. Only one of these drivers (U3) is shown.

The driver receives power from the regulated power line VCC. A decoupling capacitor has been included for each driver chip. The anodes of the LEDs (D1-D8 and D25-D32) are connected to the unregulated power line (VIN), and the cathodes to one of the output pins of the driver. The TLC5941 implements a constant-current sink driver for each LED, which is controlled via a serial interface comprised of pins BLANK (2), XLAT (3), SCLK (4), SIN (5), MODE (6), and GSCLK (25). Pins BLANK, XLAT, SCLK, MODE and GSCLK of all the drivers are connected to pins PD3 (1), PB1 (13), PD4 (2), PD2 (32), and PB0 (12) of the microcontroller (**Supplementary Fig. S3**). Only for the first LED driver, pin SIN (5) is connected to pin PD1 (31) of the microcontroller. Serial connection of several LED drivers is achieved by connecting pin SOUT (24) of one driver to pin SIN (5) of the next driver. The SOUT pin of the last driver is left unconnected. Pin IREF (27) is connected to ground through a 2.2kΩ resistor. This sets the maximum current in each output of the driver to 17.8 mA[4].

Pins SIN and SCK are seen from the microcontroller as an SPI interface. However, the microcontroller is already using its only SPI module to communicate with the SD card. To solve this, the microcontroller has been configured to use its Universal Synchronous and Asynchronous serial Receiver and Transmitter (USART) Module in SPI mode[1]. For more details, consult the firmware documentation.

**Supplementary Figure S5.** Circuit board power stage schematic.

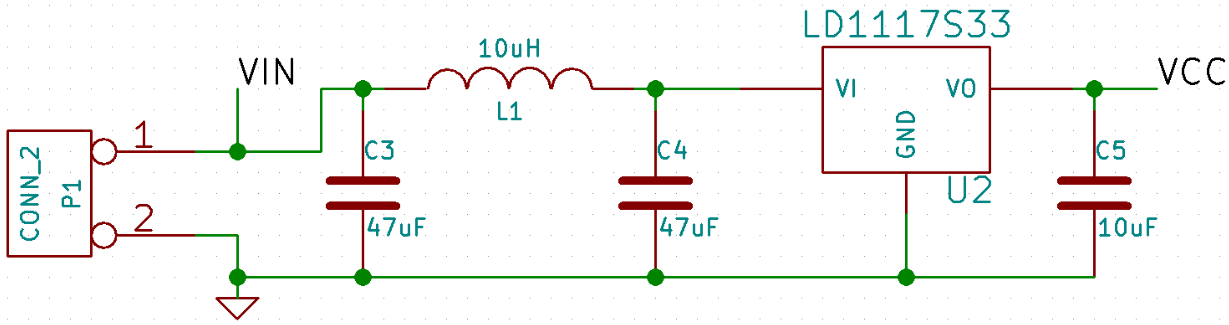


The power stage supplies electric power to the other stages. The power stage produces two power lines: regulated low power (VCC), which feeds the microcontroller and other integrated circuits, and unregulated high power (VIN), which is taken directly from the input power connector, and feeds the LEDs. Power through the LEDs is switched by the TLC5941 LED drivers; therefore the input power can be up to 17V unregulated, in accordance with the TLC5941 specifications[4]. Regulated power at 3.3V is generated by a standard voltage regulator integrated circuit LD1117S33[5], which is preceded by an LC filter (C3, C4 and L1) and followed by a stabilization capacitor (C5). Separating LED power from regulated power allows for a more stable regulated power line that is not affected by switching of large currents, while allowing for an arbitrary number of LEDs to be placed without changes in the power stage. Ground is taken directly from the input power line.

**Supplementary Figure S6.** LPA power connections.

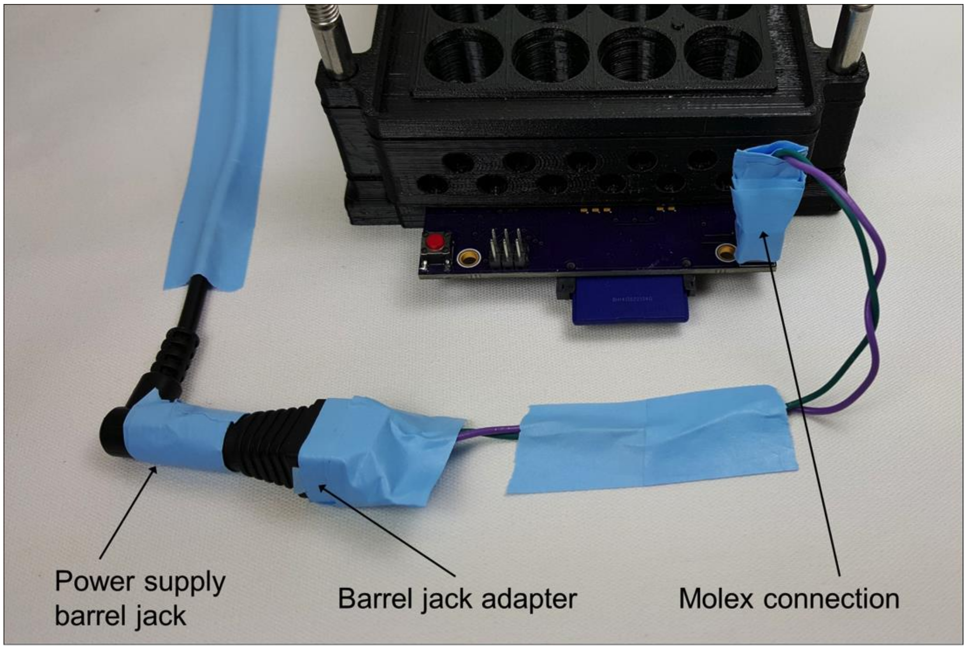


The LPA requires a continuous 5V DC power supply and power connections must be reinforced during experiments to prevent movement and mechanical stress, particularly for shaking culture. Standard laboratory tape can be used as shown to secure power connections and free wires.

**Supplementary Figure S7.** Photographs of 3D printed auxiliary parts.

**a**

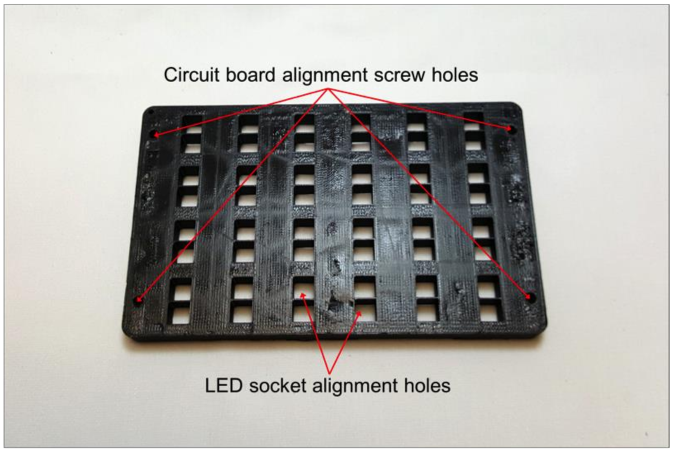


**b**

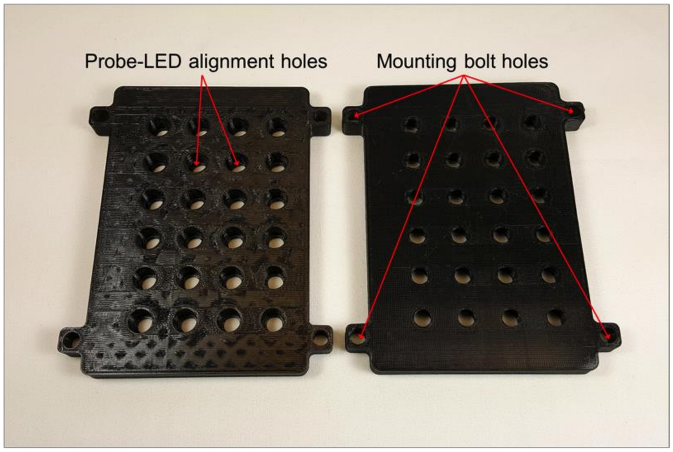


**c**

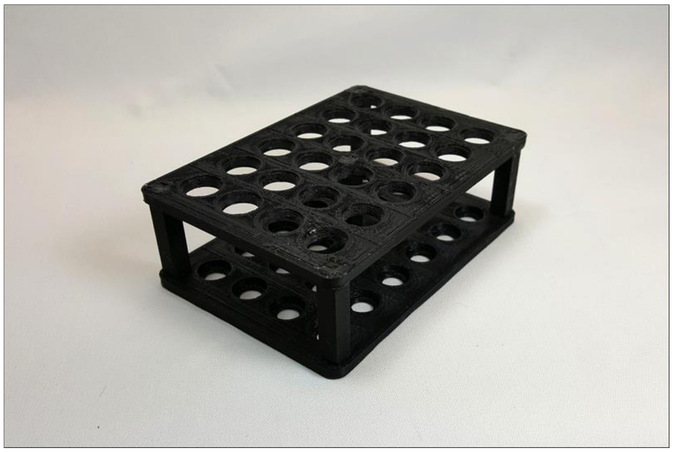

The LED socket aligner is used to align and secure LED sockets to the circuit board during soldering (see **Supplementary Method** on circuit board fabrication and assembly). (**b**) The probe adapter is used to position and align the spectrometer probe while making LED intensity measurements (see **Supplementary Method** on LED

calibration). Top and Bottom versions of the part are used for alignment with Top and Bottom LEDs, respectively. (c) The flow tube holder holds and spaces flow cytometry tubes with the same geometry as wells from the cell culture plate. The holder allows easy transfer of samples from the culture plate to flow tubes using a multichannel pipettor (LT12-1200, Ranin). The part is snapped together and epoxied from three separately printed parts: top rack, bottom rack, and pegs.

**Supplementary Figure S8.** Atmel Studio, linker libraries path.

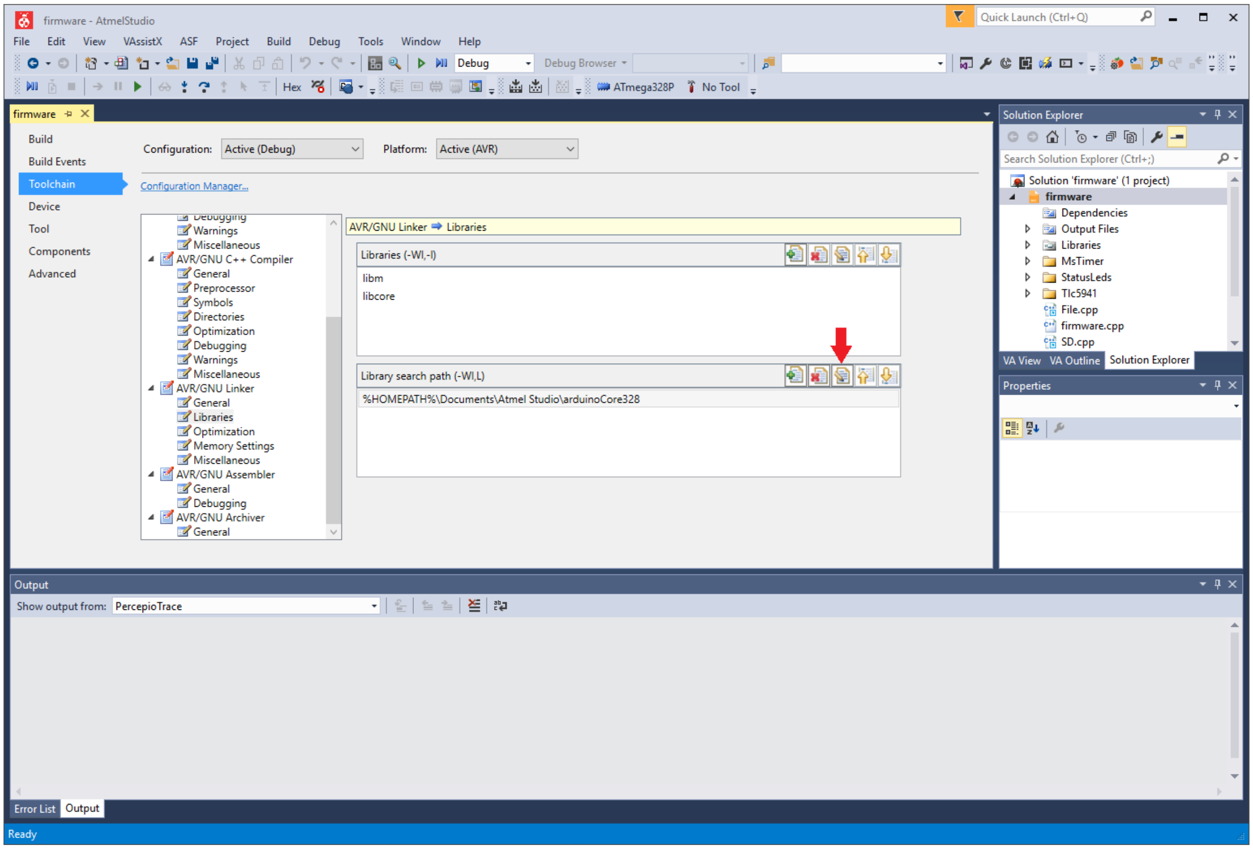


On the firmware project properties panel, the “Toolchain” option (left) has been selected, followed by “AVR/GNU Linker -> Libraries” (middle). The only item in the “Library search path” list, “%HOMEPATH%\Documents\Atmel Studio\arduinoCore328”, has been selected. The “Edit item” button is shown with a red arrow.

**Supplementary Figure S9.** Atmel Studio, compiler miscellaneous options.

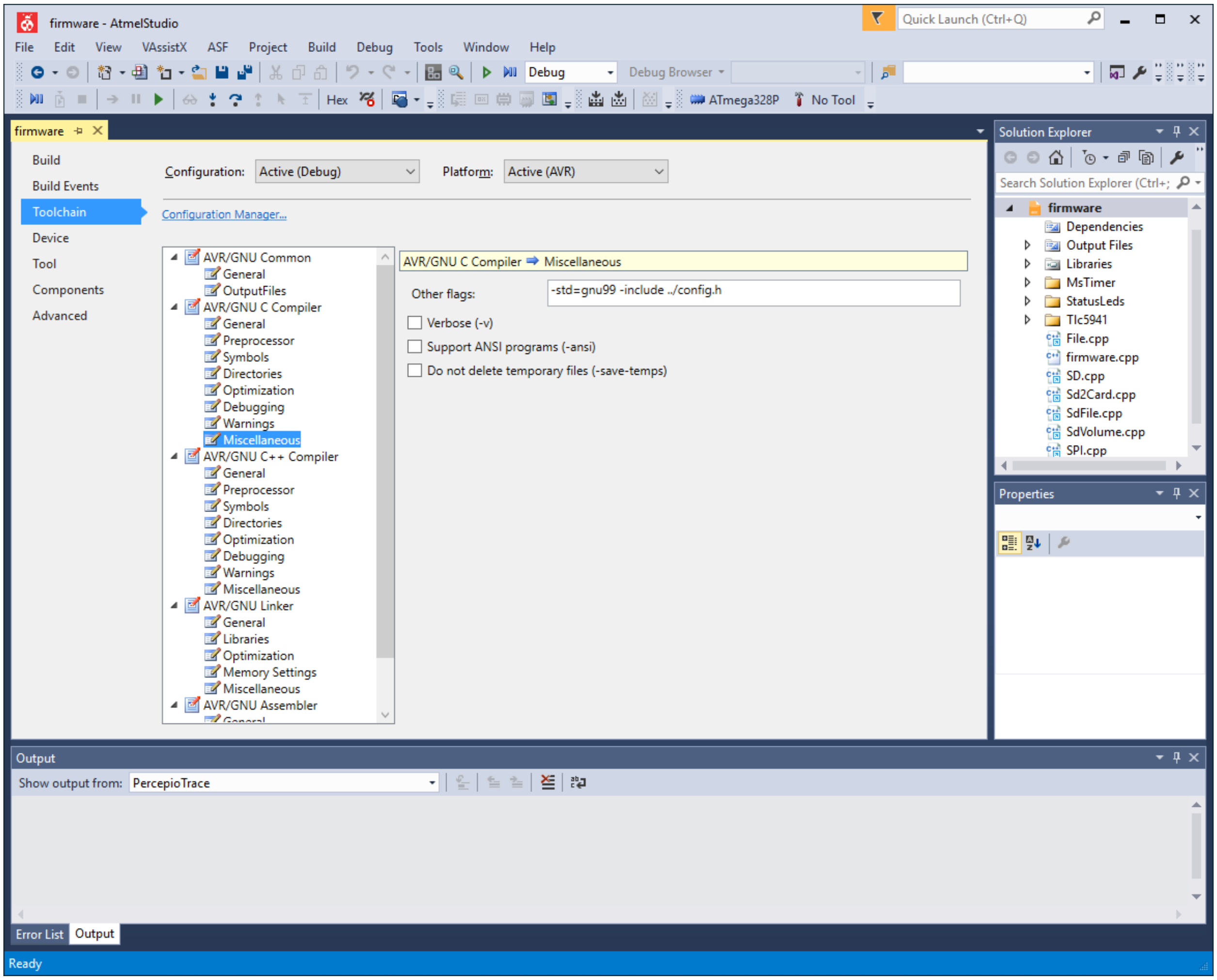

On the firmware project properties panel, the "Toolchain" option (left) has been selected, followed by "AVR/GNU C Compiler -> Miscellaneous" (middle). The figure shows the correct placement of the text "-include ../config.h" inside the "Other flags" text box.

**Supplementary Figure S10.** Atmel Studio, compiler's included directories.

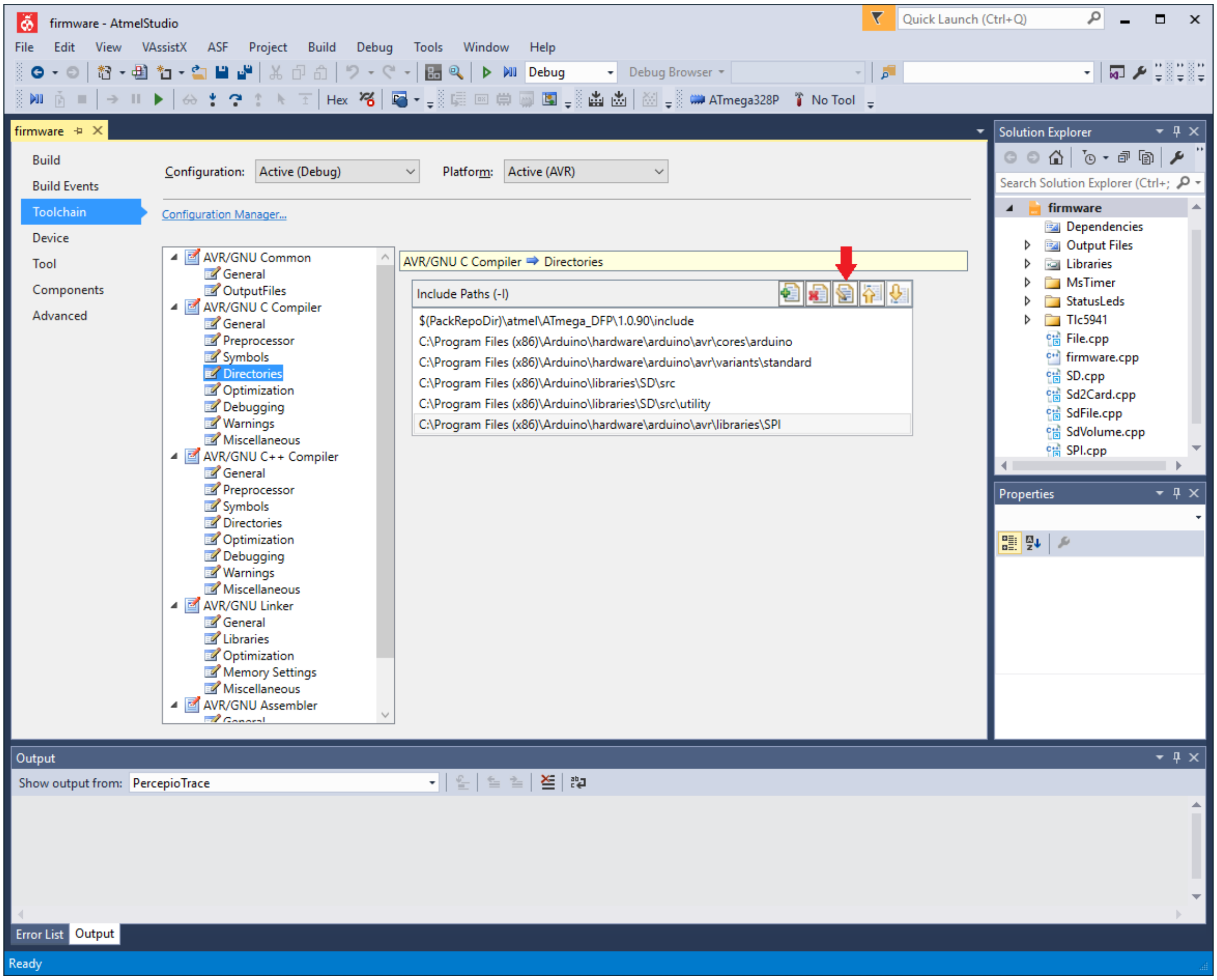


On the firmware project properties panel, the "Toolchain" option (left) has been selected, followed by "AVR/GNU C Compiler -> Directories" (middle). The "Include Paths" list is shown, with the last five entries pointing to Arduino directories. The arrow points to the "Edit item" button.

**Supplementary Figure S11.** Atmel Studio, missing referenced file.

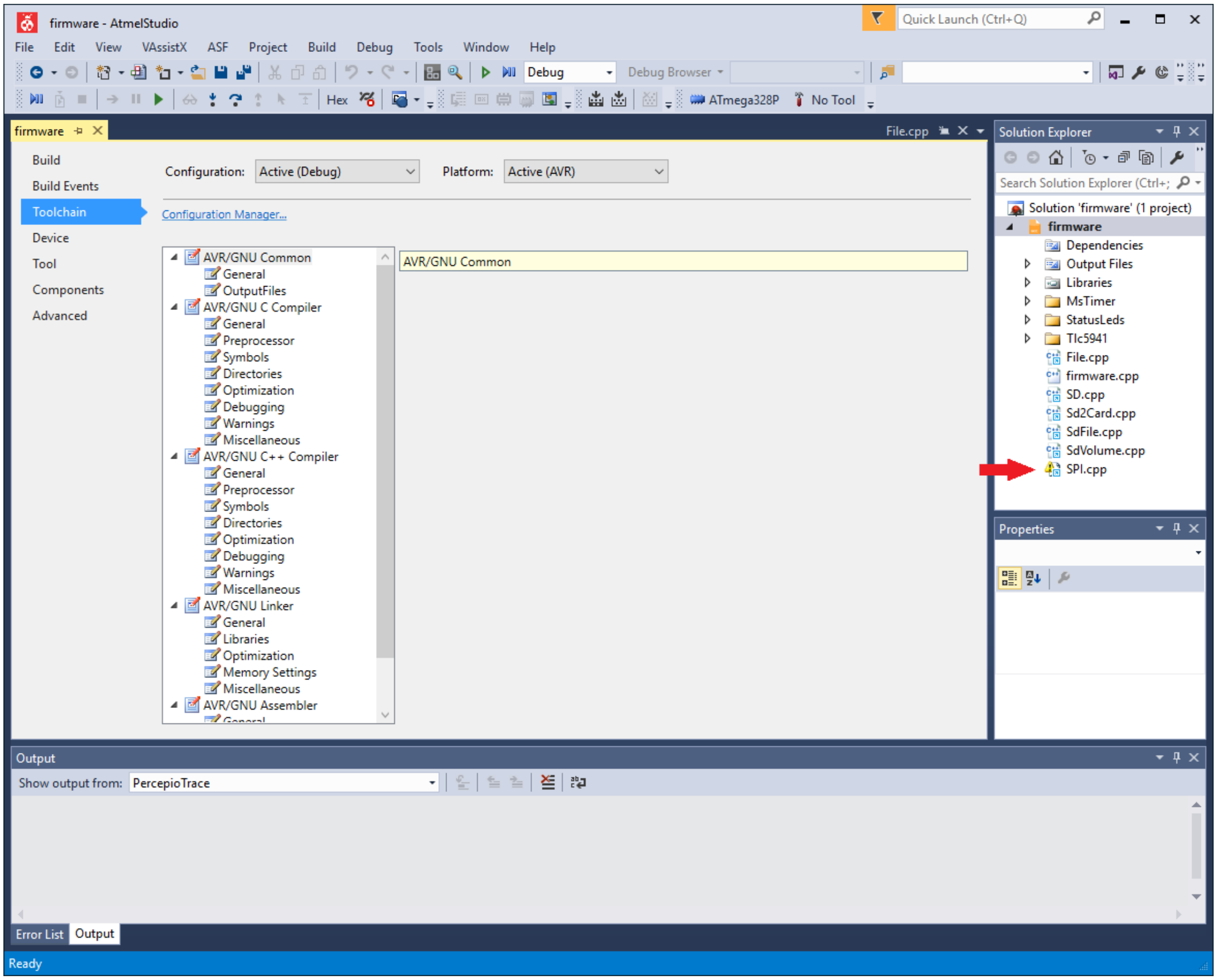


The Solution Explorer is found in the upper right part of the window. In this example, the file “SPI.cpp” has not been found, which is indicated by the yellow exclamation icon next to the file name.

**Supplementary Figure S12.** The firmware project with the config.h file open.

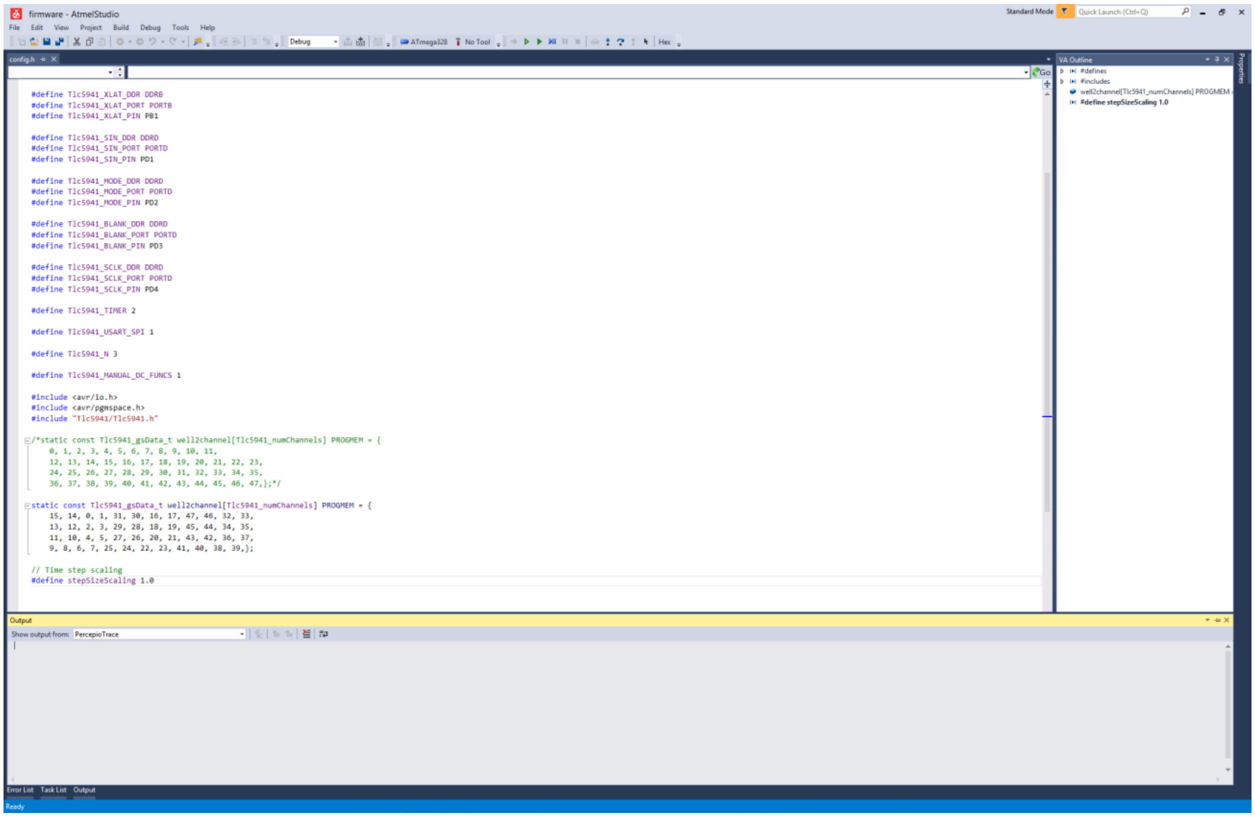

The firmware project with the config.h file open, showing the stepSizeScaling parameter used to perform LPA time calibration.

**Supplementary Figure S13.** The Device Programming dialog, after connecting to the programmer.

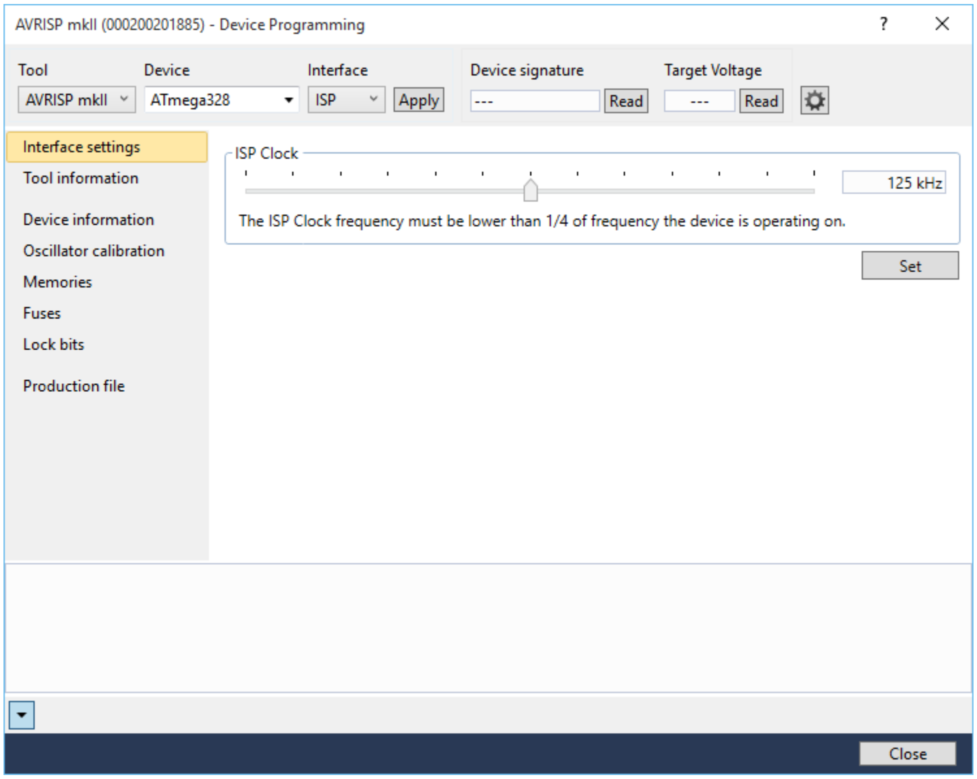


The picture shows the AVRISP mkII programmer selected under "Tool", the "ATmega328" device selected under "Device", and the "ISP" Interface selected under ISP. Everything below these options appears after clicking the "Apply" button.

**Supplementary Figure S14.** State transition diagram of the LPA firmware.

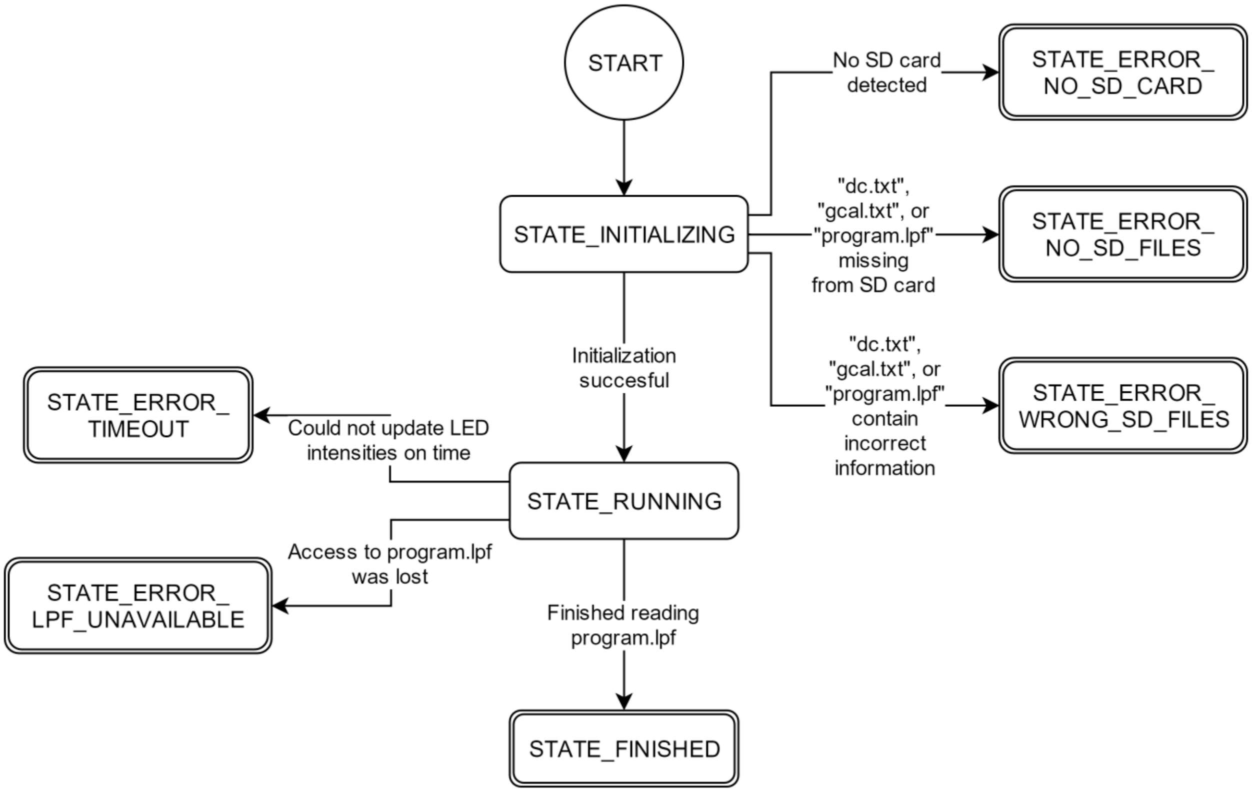


In this diagram, rounded squares denote states, rounded squares with double edges denote terminal states, and arrows denote transitions. The circle labeled as START denotes the state of the program upon booting.

**Supplementary Figure S15.** Close-up photograph of LED and LED socket friction fit.

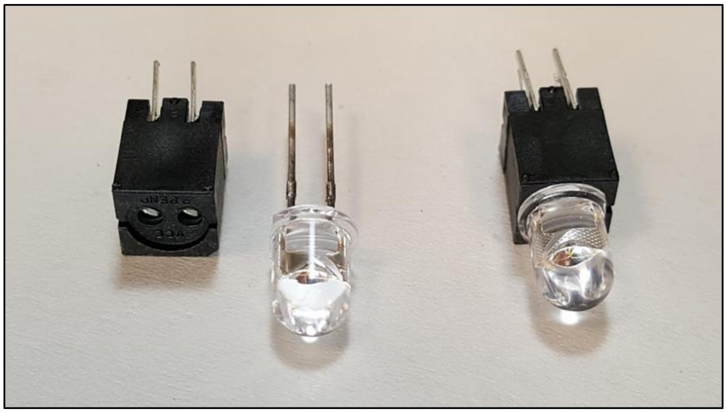

LEDs are positioned, aligned, and held in place by mating with an LED socket (**Supplementary Table S1**) via friction fit. This fixture system allows LEDs in the LPA to be easily be installed, removed, and reconfigured.

**Supplementary Figure S16.** Detailed photographs of 3D printed LPA parts.

**a**

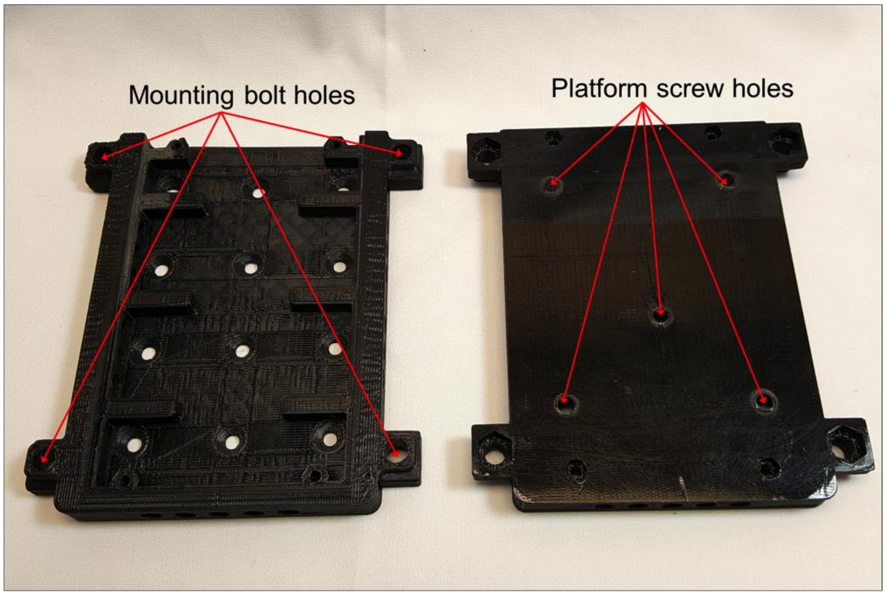


**b**

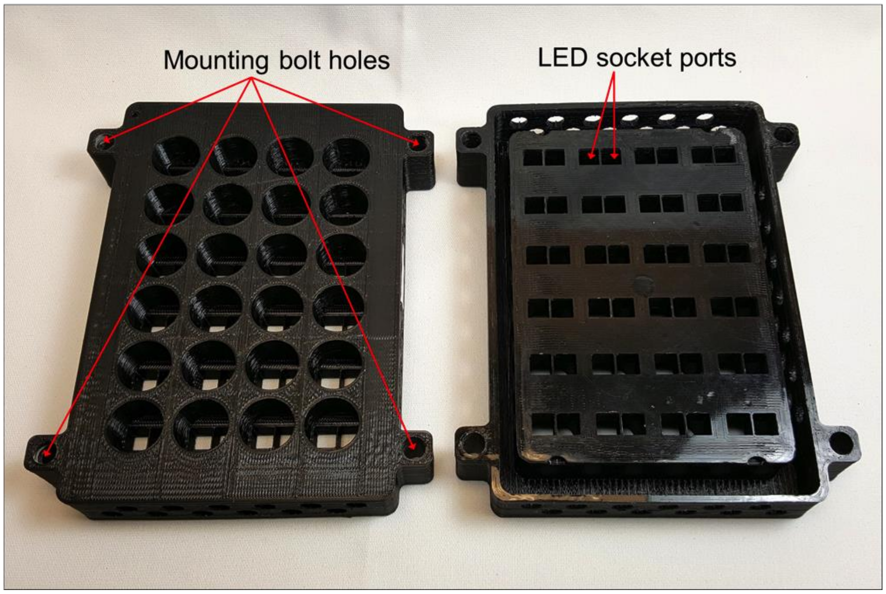

**c**

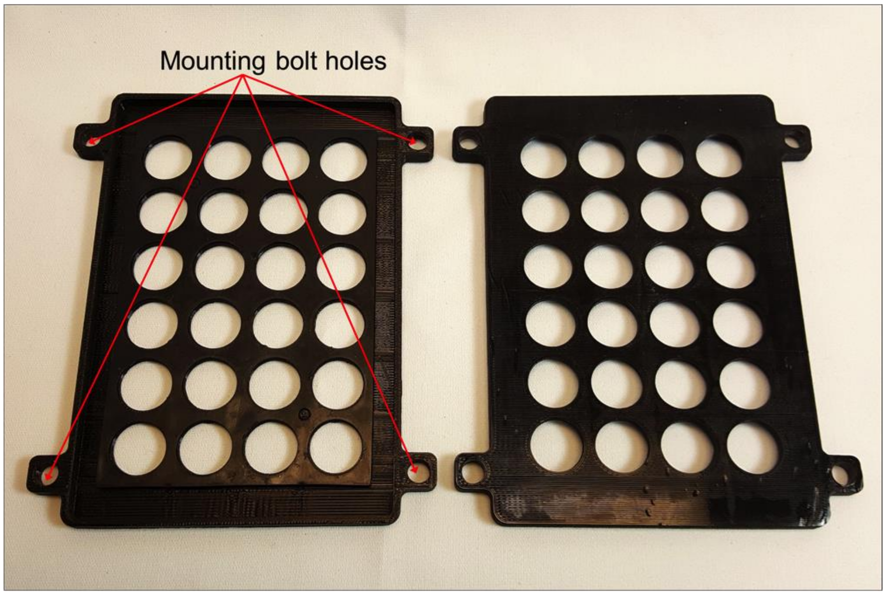


**d**

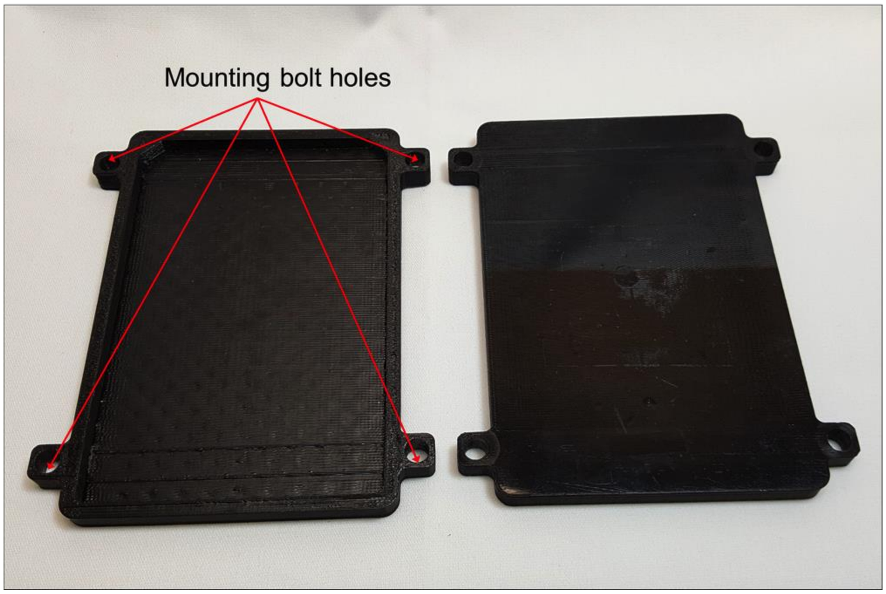


Bottom and top perspectives of 3D printed LPA parts are shown. These include (**a**) mounting plate, (**b**) LED spacer, (**c**) cell culture plate adapter, and (**d**) device lid. If shaking culture is required, the mounting plate platform holes must be designed for a particular platform. The two mounting plates shown were designed for two different shaking platforms.

**Supplementary Figure S17.** Photograph and schematic of culture plate used in the LPA.

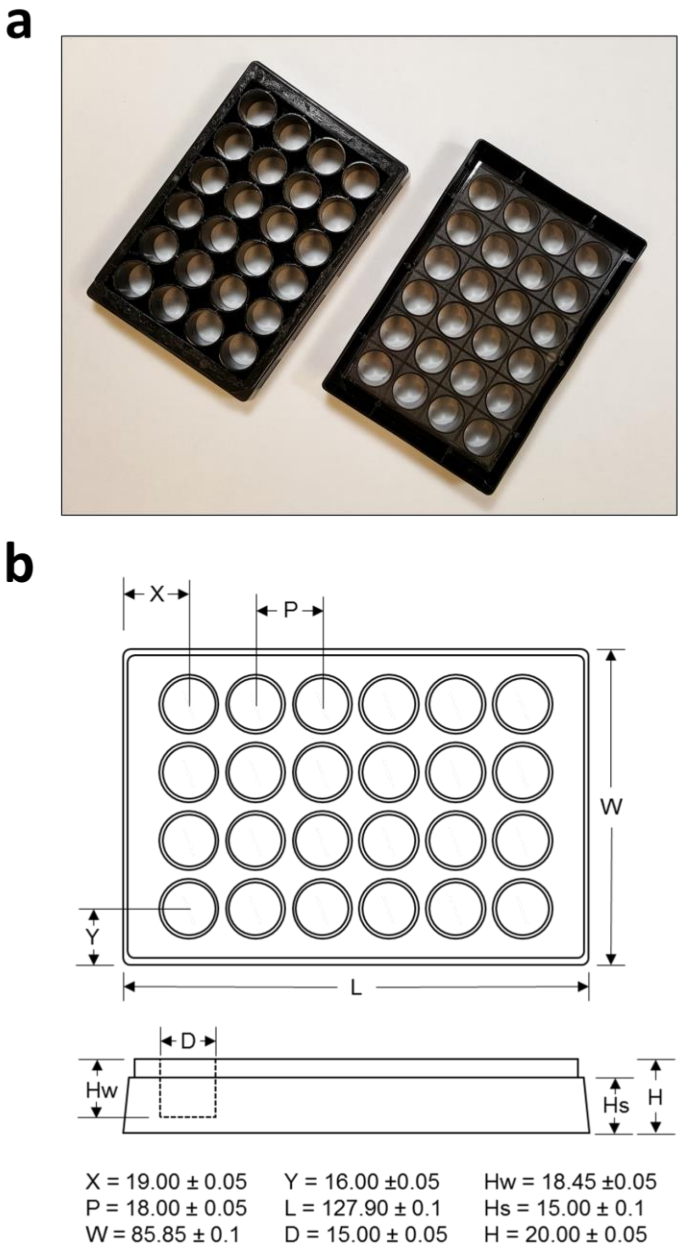


**(a)** Top and bottom views of the 24-well culture plate (AWLS-303008, ArcticWhite LLC) used in the LPA. **(b)** A schematic (adapted from supplier schematic) showing the important dimensions (mm) of the plate. The original schematic can be requested from ArcticWhite LLC.

**Supplementary Figure S18.** Photographs of LPA gaskets.

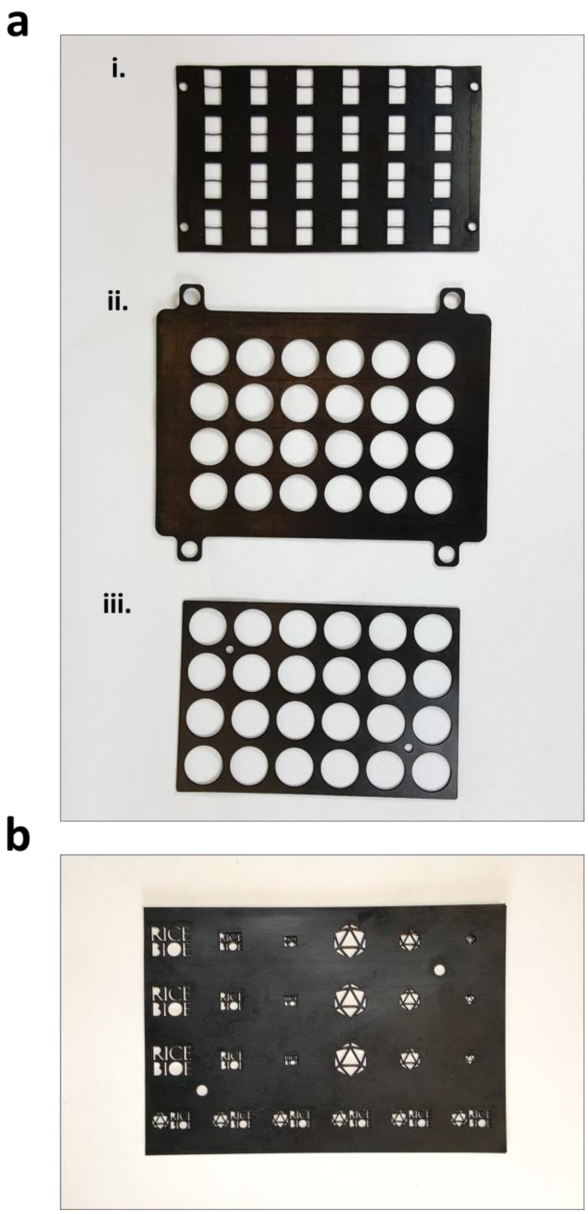


LPA gaskets are laser cut (see **Supplementary Method** on Laser cutting gaskets) from nitrile sheets (**Supplementary Table S6**) and prevent light leakage and crosstalk between wells. **(a)** The (i) circuit board, (ii) LED spacer, and (iii) cell culture plate adapter gaskets are shown. Gasket names are based on the layer they lay atop. Though not necessary, we found it convenient to superglue on the cell culture plate adapter gasket. **(b)** A plate adapter gasket which has been laser cut with patterns centered with wells of the culture plate.

**Supplementary Figure S19.** Visualization of staggered-start waveform segmentation for each dynamic waveform.

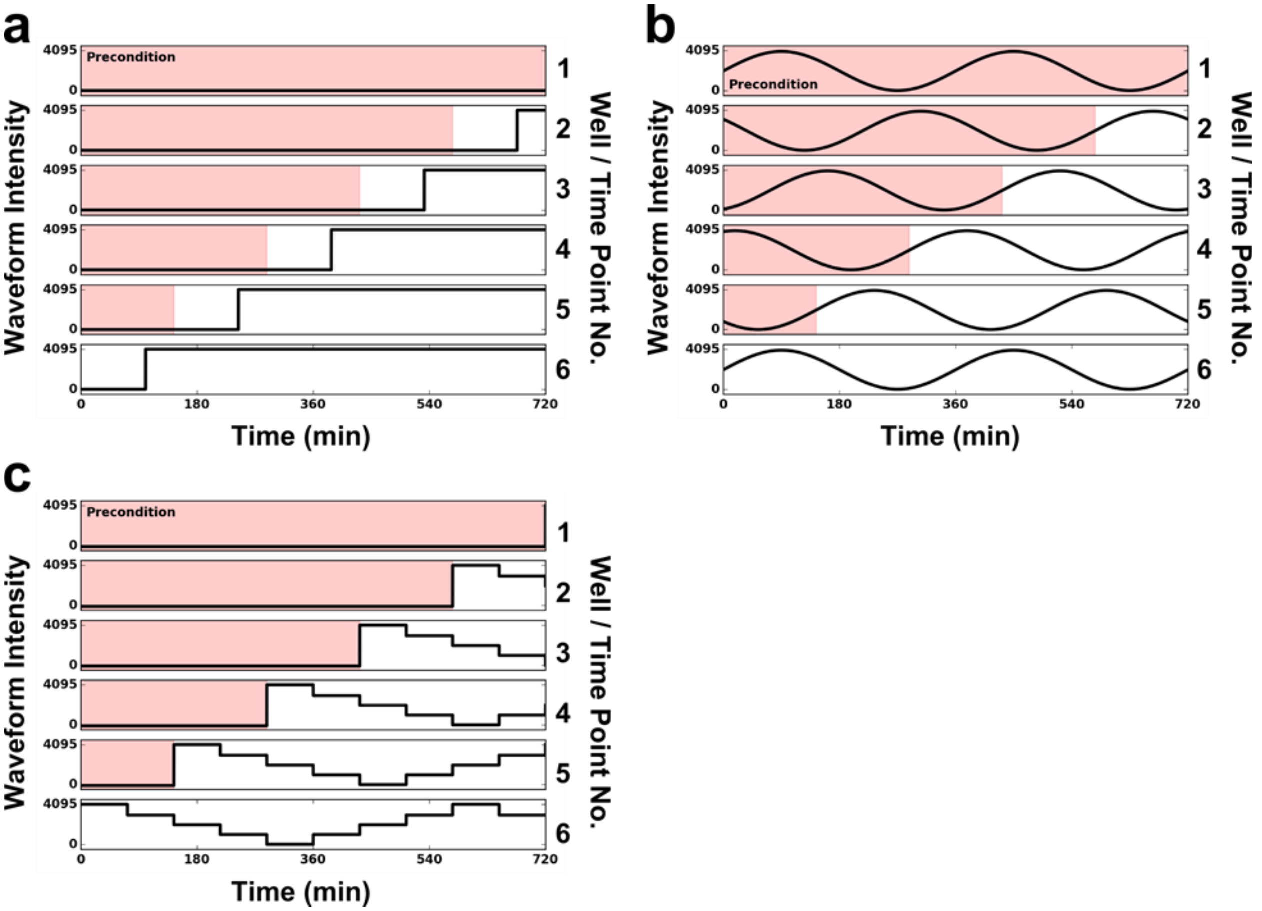


This schematic demonstrates how the staggered-start algorithm is used to produce light time courses corresponding to desired time points in an experiment for each dynamic waveform in Iris: **(a)** a step input, with the step occurring at t=100min; **(b)** a sine waveform with 360min period; and **(c)** an arbitrary waveform.

In order to perform dynamic light experiments, the input signal applied to each well in an experiment is staggered such that at the end of the program, that well will end at the desired time point in the waveform, as previously validated using our test-tube based Light Tube Array[6]. The time difference created by staggering the input is filled by exposing the well to the Preconditioning light condition (**Supplementary Table S7**) for all times before the time-shifted waveform begins (*red overlay*). For example, the t=560min time point (*Well 5, above*) in a 720min experiment will experience the Preconditioning light condition for 160min, and then begin the staggered program. It will experience the first 560min of the waveforms in each of its LED channels, at which point the experiment will end. This procedure is repeated for all wells (time points) in an experiment, and can be visualized in Iris under Well View.

The plots above demonstrate this process for 6 equally-spaced time points (subplots corresponding to the right axis) on each dynamic waveform. In this example, Well 1 corresponds to the t=0min time point, and therefore experiences the Precondition input (*red overlay*) for the entire experiment, while Well 6 corresponds to the t=720min time point, and experiences no preconditioning.

**Supplementary Figure S20.** Photograph of light intensity probe measurement setup.

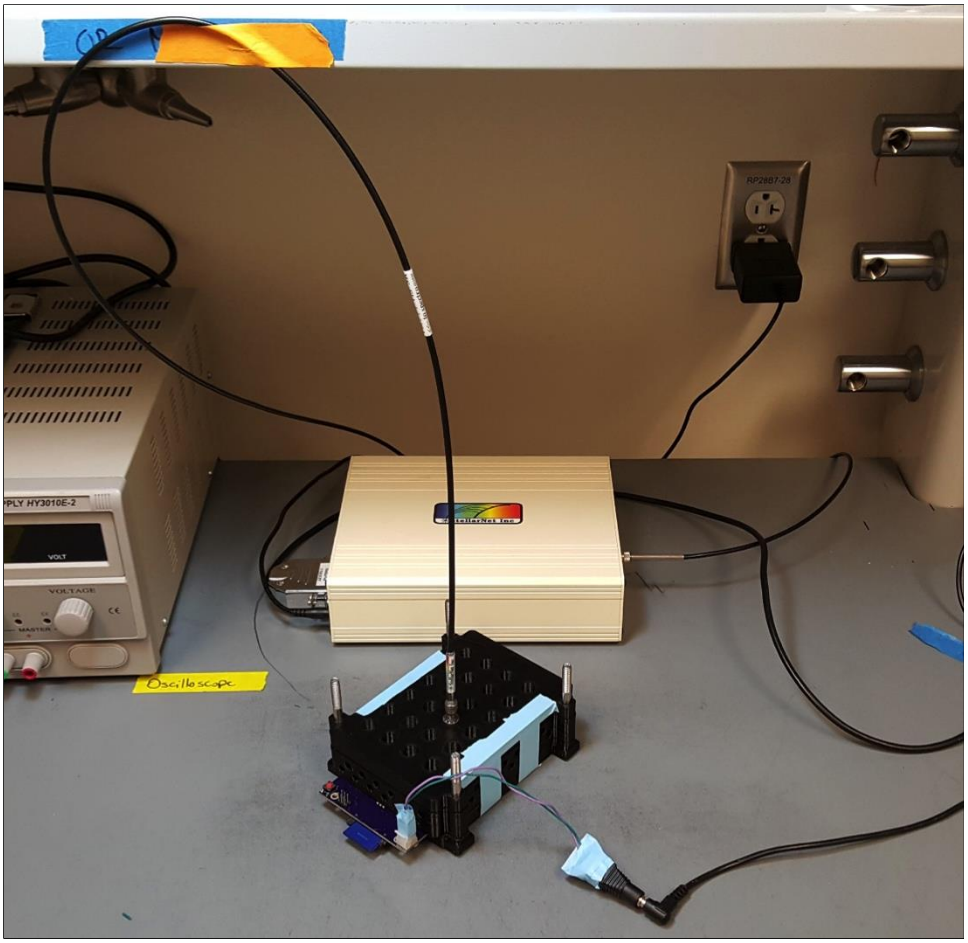


An example of the setup used to make LED intensity measurements with the probe spectrometer is shown. The LPA is assembled from the mounting plate to LED spacer & gasket and the probe adapter is installed as the top layer. Tape is used to compress layers together. The probe fits snuggly within the probe-LED alignment holes and is positioned directly above the LED being measured. Instructions for measurements and calibration with the probe spectrometer can be found in the **Supplementary Method** on LED calibration.

**Supplementary Figure S21.** LED outputs before and after image analysis calibration.

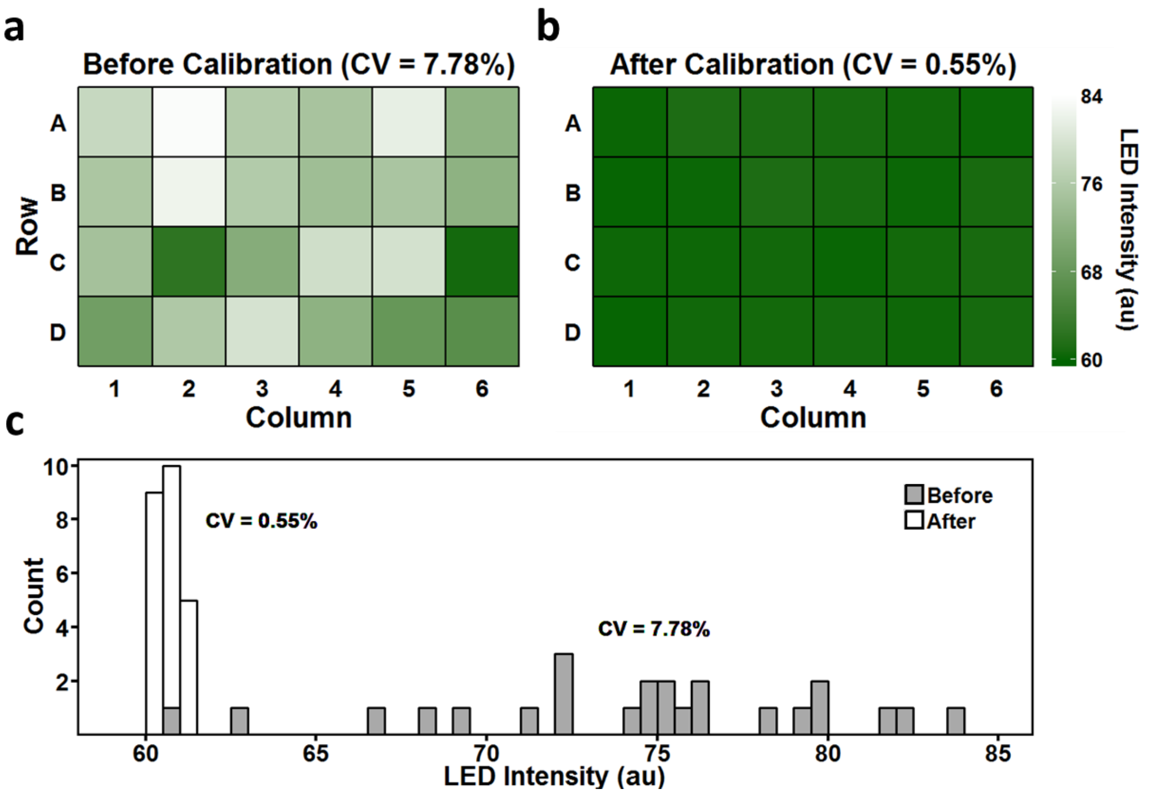


Representative comparison of LED intensity output before and after the image analysis calibration procedure (see **Supplementary Method** on LED calibration). (**a**) Heatmap showing distribution of 678 nm LED intensities prior to calibration (CV = 7.78%). (**b**) Heatmap showing intensity distribution of the same LEDs as in (**a**), after the image analysis calibration (CV = 0.55%), in which LEDs are scaled to the dimmest LED. (**c**) Histogram of LED intensity outputs from (**a**) and (**b**). LED intensities are measured as the sum of pixel intensity inside the LPA well containing the LED.

**Supplementary Figure S22.** Dependence of *S. cerevisiae* yMM1081 growth on 467nm light intensity.

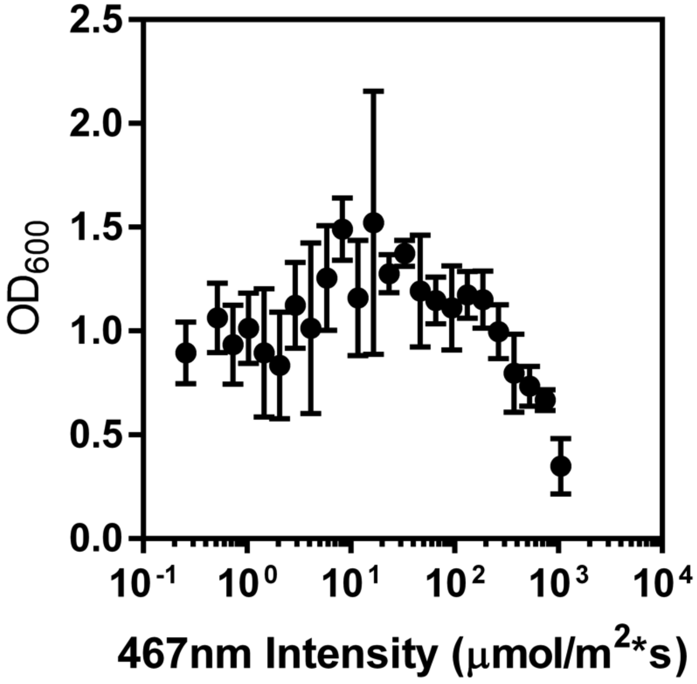


A blue light transfer function experiment was conducted on S. cerevisiae strain yMM1081 as described in the main text. After chilling on ice for 15 min 50 μL samples were transferred to wells of a 96-well microplate and measured for absorbance with a plate reader (M200 Pro, TECAN). Absorbance measurements were converted to $OD_{600}$ by an empirical mapping between plate reader and spectrophotometer (Cary50 UV/vis, Agilent, Inc.) for strain yMM1081:

$$OD_{600} = 8.555A_{plate\ reader} - 0.3345$$

Error bars represent standard deviation of three replicate transfer function experiments. The decline in $OD_{600}$ at high intensity is likely due to a combination of elevated temperature inside the device and phototoxicity, though the relative contribution of each is unclear.

**Supplementary Figure S23.** Scalability of LPAs.

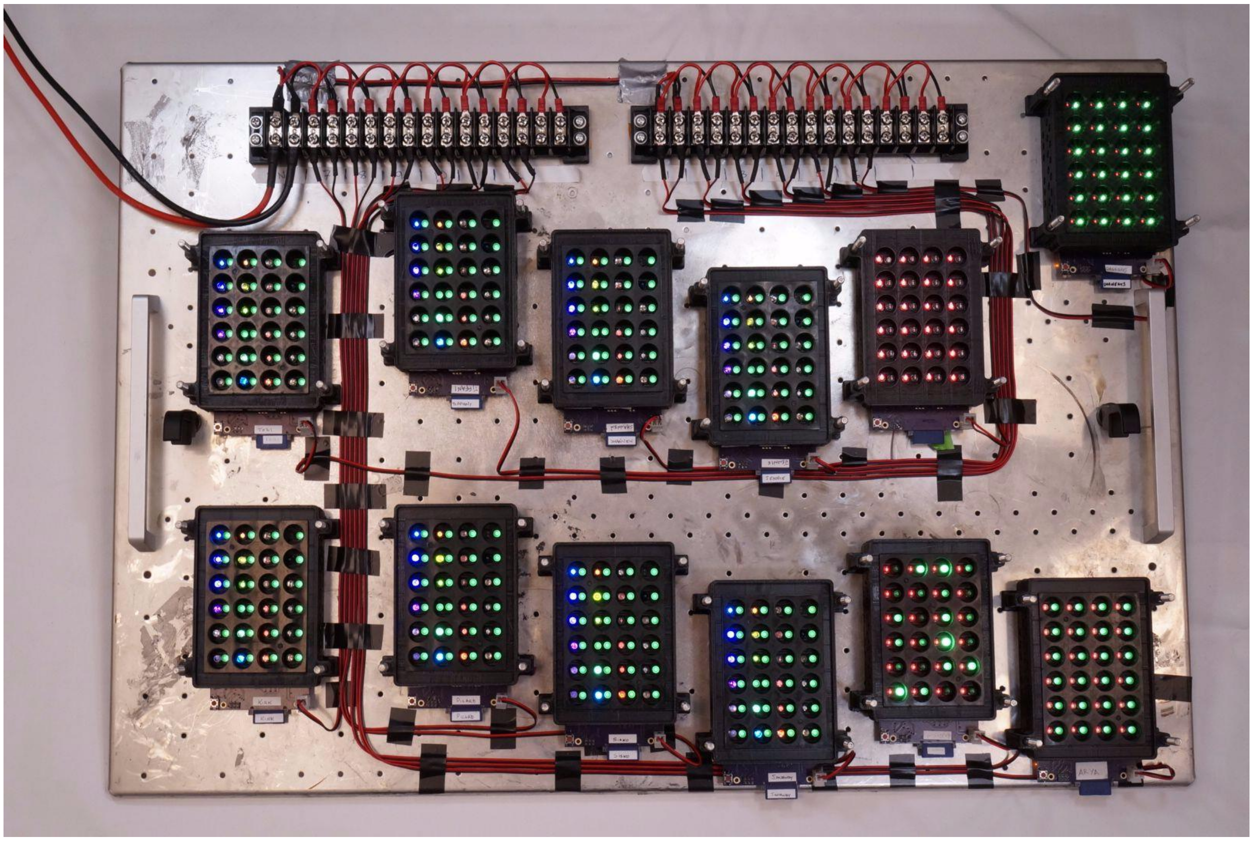

The compact design of the LPA makes it possible to install and run in parallel several LPAs within a single incubator. Here, twelve LPAs with various LED loadouts are shown mounted to a common sized shaking incubator (SI9/SI9R, Shel Labs) platform. Each LPA is connected to a power and ground terminal on one of the screw terminal strips (6ZEJ5, Grainger) which are also mounted to the platform and which receive power from a single 5 V supply (RS-150-5, Meanwell) (not shown).

## Supplementary Tables

**Supplementary Table S1.** Circuit board components parts list.

| Description | Prod. #[ab] | Quantity Per LPA | Notes |
|---|---|---|---|
| ATMega328A microcontroller (QFP) | ATMEGA328-AU-ND | 1 | |
| TLC5941 LED driver (TSSOP) | 296-18617-5-ND | 3 | |
| 16M resonator (SMD) | 535-10008-1-ND | 1 | Built-in load capacitors. |
| 50k resistor (0805) | P47KACT-ND | 5 | |
| 10k resistor (0805) | P10KACT-ND | 2 | |
| 2.2k resistor 0.5% (0805) | RR12P2.2KDCT-ND | 3 | |
| 330 resistor (0805) | 1276-5519-1-ND | 3 | |
| 0.1 uF capacitor (0805) | 1276-1007-1-ND | 5 | |
| 10uF capacitor (0805) | 311-1355-1-ND | 1 | |
| 47 uF electrolytic SMD | 493-2202-1-ND | 2 | |
| 10 uH inductor SMD | 399-9595-1-ND | 1 | |
| 3.3V 0.8A power regulator | 497-1242-1-ND | 1 | |
| Reset switch | CKN9084CT-ND | 1 | |
| SD card socket | 101-00313-68-02CT-ND | 1 | |
| SMD LED Green | 475-1410-1-ND | 1 | In-progress indicator LED |
| SMD LED Red | 475-1415-1-ND | 1 | Error indicator LED |
| SMD LED Yellow | 475-2560-1-ND | 1 | Experiment complete indicator LED |
| LED socket | PCV_220-ND | 48 | |
| Breakaway headers (40 pos, 0.100") | A34253-40-ND | 0.15 | |
| Molex 2x1 male connector high-power | WM18823-ND | 1 | |
| Molex 2x1 female connector | WM18813-ND | 1 | |
| Molex connector crimp terminal | WM18820CT-ND | 2 | |
| Male barrel jack 2.1mm | CP-5-ND | 1 | |
| 5V wall mount power supply | 418-TRG1505-V | 1 | 90-264V AC to DC wall adapter. |

[a] Abbreviations: Prod., product. [b] All components supplied by Digikey, except 5V wall mount power supply, which is supplied by Mouser.

**Supplementary Table S2.** Equipment required for circuit board soldering procedure.

| Equipment | Note |
|---|---|
| Toaster oven | |
| Fume hood | |
| Soldering kit | Iron, solder, desoldering braid. |
| Dissecting microscope | Not required but strongly recommended. |
| Electrostatic-safe tweezers | |
| Digital Multimeter (DMM) | |
| Printed circuit board stencils | |
| Solder paste | |
| Printed circuit board stencils | File: lpa-B.Mask.gbs |
| Wire strippers | |
| Molex terminal crimper | |
| Multicore wire | ~22G |

**Supplementary Table S3.** Error conditions, as indicated by status LEDs.

| LEDs | Description | Solution | Detected at |
|---|---|---|---|
| ON: off<br>FIN: off<br>ERR: on | No SD card has been detected. | Insert or replace the SD card. | Initialization |
| ON: off<br>FIN: on<br>ERR: on | Even though the SD card was detected, at least one of the following files was not: “dc.txt”, “gcal.txt”, and “program.lpf”. | Make sure the SD card contains these three files. | Initialization |
| ON: on<br>FIN: off<br>ERR: on | Either “dc.txt” or “gcal.txt” contain an incorrect number of values, or values are outside the acceptable range. Or headers in “program.lpf” indicate potentially incorrect information or incompatibility with this device. | Verify that the number of values specified in “dc.txt” and “gcal.txt” is appropriate. Only use LPF files from a verified source (e.g. Iris). | Initialization |
| ON: blinking<br>FIN: off<br>ERR: blinking | The device has not been able to maintain the specified resolution. | Try increasing the time step for light intensity update. | Runtime |
| ON: off<br>FIN: off<br>ERR: blinking | The file has become unavailable in the middle of the run. | Verify that your SD card is properly inserted in the SD card slot. | Runtime |

The status LEDs indicate the following:

ON (green): Indicates proper function of the plate device. Blinking with a one-second period indicates that the device is running and reading a LPF from the SD card.

FIN (yellow): Indicates that the execution of the light program specified in the LPF has finished.

ERR (red): Indicates some error in the execution of the LPF.

**Supplementary Table S4**. States of the LPA firmware.

| State | Description |
|---|---|
| STATE_INITIALIZING | The device will be in this state upon booting. In this state, the device is initializing resources and running checks on the LPF. |
| STATE_RUNNING | The device has passed its initialization routines, and it is currently executing the program contained in the LPF. |
| STATE_FINISHED | The device has finished execution of the LPF successfully. |
| STATE_ERROR_NO_SD_CARD | The device was not able to recognize an SD card. |
| STATE_ERROR_NO_SD_FILES | The device was not able to find all of the following files in the SD card: “dc.txt”, “gcal.txt”, and “program.lpf”. |
| STATE_ERROR_WRONG_SD_FILES | Either “dc.txt”, “gcal.txt”, or “program.lpf” are improperly formatted or corrupt. |
| STATE_ERROR_TIMEOUT | The device was not able to maintain the specified time resolution. |
| STATE_ERROR_LPF_UNAVAILABLE | The LPF has become unavailable in the middle of the run. |

The first three states correspond to normal operation of the device. STATE_RUNNING is indicated by blinking of the “ON” status LED. STATE_FINISHED is indicated by the “FIN” status LED. The last five states correspond to error situations, and have a one-to-one correspondence with error LED blinking patterns (**Supplementary Table S3**).

**Supplementary Table S5.** LEDs used in this study and other LED examples.

| ID | Mfg. (Supplier)[a] | Mfg. Prod. #[a] | Mfg. Peak (nm)[a] | PEAK (nm)[b] | CENTROID (nm)[b] | FWHM (nm)[ab] |
|---|---|---|---|---|---|---|
| 310-MB | MB | UF1VL-1H21 | 310 | | | |
| 355-LS | LS | L5-1-U5TH15-1 | 355 | | | |
| 361-LS | LS | L5-0-U5TH15-1 | 361 | 362.18 | 363.95 | 10.69 |
| 380-SB[c] | SB | RL5-UV0230-380 | 380 | 380.02 | 381.84 | 10.27 |
| 405-SB | SB | RL5-UV0430-400 | 405 | 405.08 | 406.47 | 17.94 |
| 430-CM | CM (MS) | 606-CMD383UBC/H2 | 430 | 428.89 | 437.85 | 60.79 |
| 470-SB | SB | RL5-B2545 | 470 | 465 | 467.29 | 20.82 |
| 490-LS | LS | L4-0-T5TH15 | 490 | 496.77 | 503 | 33.22 |
| 505-LS | SB | RL5-A9018 | 505 | 508.15 | 512.58 | 24.61 |
| 520-KB | KB | WP7083ZGD | 520 | 529.14 | 533.28 | 27.43 |
| 555-LS[c] | LS | RL5-G8045 | 555 | 544.7 | 556.05 | 27.5 |
| 570-KB[c] | KB | WP7113CGCK | 570 | 570.87 | 571.35 | 14.26 |
| 590-SB | SB | RL5-Y3545 | 590 | 592.52 | 591.17 | 14.3 |
| 605-SB | SB | RL5-O4030 | 605 | 609.15 | 609.33 | 16.65 |
| 630-SB | SB | RL5-R3545 | 630 | 638.36 | 636.19 | 13.94 |

| | | | | | | |
|---|---|---|---|---|---|---|
| 660-LS | LS | L2-0-R5TH50-1 | 660 | 646.73 | 646.79 | 22.33 |
| 680-MT[c] | MT (DK) | MTE6800N2-UR | 680 | 674.7 | 674.05 | 23.82 |
| 700-LU[c] | LU | 696-SSL-LX5093HT | 700 | 699.02 | 703.77 | 92.86 |
| 720-MB | MB | L720-03-AU | 720 | 722.03 | 720.99 | 24.92 |
| 735-MB[d] | MB | L735-05-AU | 735 | 735.71 | 733.43 | 26.85 |
| 740-MT | MT (DK) | MTE1074N1-R | 740 | 739.45 | 737.71 | 27.34 |
| 760-MB | MB | L760-04-AU | 760 | 755.5 | 753.36 | 27.4 |
| 780-MB | MB | L780-04-AU | 780 | 786.27 | 783.54 | 27.52 |
| 850-VI | VI (MS) | 782-TSHG6200 | 850 | 853.02 | 849.47 | 33.99 |
| 940-VI[c] | VI (MS) | 782-TSAL6400 | 940 | 947.51 | 947 | 50.94 |
| 1200-MB | MB | L1200-05 | 1200 | | | |
| 1300-MB | MB | L1300-05 | 1300 | | | |
| 1550-MB | MB | L1550-05 | 1550 | | | |

[a] Abbreviations: Mfg., manufacturer; Prod., product; FWHM, full width at half maximum; LS, LED supply; SB, Superbright LEDs; CM, Chicago Miniature; MS, Mouser; KB, Kingbright; MT, Marktech; DK, DigiKey; LU, Lumex; MB, Marubeni; VI, Vishay Infrared.

[b] Our own measurements with a probe spectrometer (StellarNet Inc, photodetector: EPP2000 UVN-SR-25LT-16, probe: F600-UV-VIS-SR, software: SpectraWiz) .

[c] LEDs not used in CRY2-CIB1 Y2H FAS due to low maximum intensity.

[d] 735-MB only used in PHYB/VNP-PIF6 experiments.

LEDs used in this study indicated in green. Examples of LPA compatible LEDs outside the range of wavelengths used in this study indicated in red.

**Supplementary Table S6.** Non-electrical LPA components.

| Description | Supplier | Prod. #[a] | Quantity Per Device | Notes |
|---|---|---|---|---|
| **24-well culture plate** | ArcticWhite LLC | AWLS-303008 | 1 | 24-well, opaque w/transparent bottom. Reusable. |
| **M6 bolts** | McMaster-Carr | 91287A252 | 4 | 70mm/6mm(10mm hex)/M6 (Length/Diameter/Thread) |
| **Wing nut** | McMaster-Carr | 92124A530 | 4 | Black nitrile. |
| **Adhesive foil** | VWR | 60941-126 | 1 | Disposable. |
| **Nitrile rubber sheet** | Grainger | 1DPZ3 | ~0.5[b] | 12"x12"x1/32" Length/Width/Thickness |

[a] Abbreviations: Prod., product. [b] Material laser cut to make device gaskets. One sheet is enough for about two devices.

**Supplementary Table S7.** Iris preconditioning light input for each function.

| Input Waveform | Precondition Value |
|---|---|
| Constant | N/A |
| Step | Input intensity before step (i.e. the step offset, c). |
| Sine | Because sines are periodic, no precondition intensity is necessary; the function is simply phase shifted by the appropriate amount. |
| Arbitrary | The precondition light intensity for Arbitrary waveforms is set by the user in the waveform input spreadsheet. |

**Supplementary Table S8.** LPF file specifications.

| Bytes | Variable Name | Precondition Value |
|---|---|---|
| 0-3 | FILE_VERSION | LPF version number (currently 1) |
| 4-7 | NUMBER_CHANNELS | Number of channels -- Note: This is the total number of LED channels (e.g. 2*24 = 48), not per well |
| 8-11 | STEP_SIZE | Time step size, in ms (default: 1000ms to limit total program file size) |
| 12-15 | NUMBER_STEPS | Number of time points (total program time / STEP_SIZE + 1) |
| 16-31 | --empty-- | Reserved space for future header fields; all set to 0 |
| >= 16 | N/A | Intensity values of each channel per time point. For each value, two bytes will be used as a long 16-bit int |

The LPF binary should not need to be examined directly during the standard workflow, however its format is detailed below should it be necessary:

The LPF file has a header segment encoded by 32-bit (4-byte) ints, which specify the number of channels (total number of LEDs, typically 24), the time step between updating LED intensities, and the number of time steps encoded in the file.

LED intensity values are listed in a depth-first manner (i.e. all LEDs for a particular well, then proceeding to the next well), moving top-to-bottom, and left-to-right across the plate device. The LED order is a hard-coded parameter for each device, and is dependent on the particular configuration of the PCB.

Because of the above structure, specifically that every time step is encoded explicitly, and that each is encoded using a 16-bit (2 byte) integer, file sizes can quickly become very large at small time steps or long experiment lengths. To keep the file size reasonable, we limit time steps to 1 sec, minimum.

**Supplementary Table S9.** Best fit parameters and standard errors for CcaS-CcaR steady-state transfer function.

| | Red Intensity[a] / Parameter | 0 | 2 | 5 | 12 |
|---|---|---|---|---|---|
| Best fit value | *a* (MEFL) | 85200 | 85200 | 85200 | 85200 |
| | *b* (MEFL) | 19880 | 19880 | 19880 | 19880 |
| | *n* | 2.651 | 2.651 | 2.651 | 2.651 |
| | *k* ($\mu mol\ m^{-2}\ s^{-1}$) | 0.2165 | 0.4275 | 0.823 | 1.502 |
| Fit standard error | *a* (MEFL) | 430 | 430 | 430 | 430 |
| | *b* (MEFL) | 260 | 260 | 260 | 260 |
| | *n* | 0.060 | 0.060 | 0.060 | 0.060 |
| | *k* ($\mu mol\ m^{-2}\ s^{-1}$) | 0.0038 | 0.0075 | 0.014 | 0.026 |

[a] Intensity units are $\mu mol\ m^{-2}\ s^{-1}$.

Hill parameters *a*, *b*, and *n* are fit as shared parameters between red intensity datasets.

**Supplementary Table S10.** Best fit parameters and standard errors for CcaS-CcaR kinetic model.

| | Parameter | Best fit value | Fit standard error | Units |
|---|---|---|---|---|
| **MEFL transformed dataset**[a] | $k_g$ | 0.0229 | 0.0020 | $min^{-1}$ |
| | $k_{p,m}$ | 0.089 | 0.038 | $min^{-1}$ |
| | $k_{p,o}$ | 0.058 | 0.011 | $min^{-1}$ |
| | $k_{p,l}$[b] | 0.0272 | 0.0057 | $min^{-1}$ |
| | $k_{p,k}$[b] | 2.163 | 0.44 | (µmol $m^{-2}$ $s^{-1}$) |
| | $\tau$ | 4.5 | 5.0 | min |
| **Exponential transformed dataset**[a] | $k_g$ | 0.0213 | 0.0015 | $min^{-1}$ |
| | $k_{p,m}$ | 0.118 | 0.059 | $min^{-1}$ |
| | $k_{p,o}$ | 0.061 | 0.011 | $min^{-1}$ |
| | $k_{p,l}$[b] | 0.0272 | 0.0057 | $min^{-1}$ |
| | $k_{p,k}$[b] | 2.163 | 0.44 | (µmol $m^{-2}$ $s^{-1}$) |
| | $\tau$ | 4.4 | 6.0 | min |

[a] Parameter values were fit with exponential (arbitrary units) transformed or MEFL transformed datasets. Parameters used for gene expression programming experiments were from fits with exponential transformed datasets.

[b] $k_{p,l}$ is a light intensity independent parameter and was taken directly from Olson et al., 2014. $k_{p,k}$ was scaled from the Olson et al., Nat. Methods. 2014 value by the ratio of LPA to LTA green light intensity, as determined by the steady-state transfer function of CcaS-CcaR in these instruments.

**Supplementary Table S11.** Best fit parameters and standard errors for CRY2-CIB1 Y2H steady-state intensity transfer function and kinetic model.

| | Parameter | Best fit value | Fit standard error | Units |
|---|---|---|---|---|
| **Hill Parameters** | *a* | 136 | 19 | MECY |
| | *b* | 29.2 | 7.5 | MECY |
| | *n* | 1.35 | 0.39 | |
| | *k* | 10.7 | 2.4 | μmol $m^{-2}$ $s^{-1}$ |
| **Kinetic Parameters** | *α* | 0.00449 | 0.00042 | $min^{-1}$ |
| | *τ* | 75.1 | 7.2 | min |

**Supplementary Table S12.** Comparison of select non-neural optogenetic hardware from the literature.

| Specification | Light Plate Apparatus | Light Tube Array[6] | Optical Microtiter Plate[7] | Automated Illumination Setup[8] | LED Illuminator[9] |
|---|---|---|---|---|---|
| **Format** | 24-well culture plate | 14-mL culture tube | 96-well culture plate | multi-well plates | 24-well culture plate |
| **Approximate Size** | 0.00076 $m^3$ | 0.019 $m^3$ | Unknown | 0.043 $m^3$ | 0.00062 $m^3$ |
| **Throughput (unique light environments)** | 24 | 64 | 96 | 1[a] | 6 |
| **LED channels per sample** | 2 | 4 | 3 (1xRGB[b]) | 8 | 2 |
| **Controller** | ATMega328a | Arduino Uno | Personal computer, USB-8451 | Personal computer/TECAN M200, BLS-SA04-US | Arduino (model unknown) |
| **Intensity Control** | 12-bit PWM[b], 6-bit current control | 12-bit PWM[b], 6-bit current control | 20 µs ON/OFF control, 7-bit current control | 0.1% increments | Analog/PWM[b,c] |
| **Programming Interface** | Iris (GUI[b]), open-source | Arduino IDE (SI[b]), open-source | Labview, proprietary | Tecan iControl (GUI[b]), proprietary | Arduino IDE (SI[b]), open-source |
| **Chassis** | 3D-printed ABS[b] | Machined Aluminum, Foam | No-bottom 96-well culture plate, limited details | TECAN M200 | Laser-cut acrylic |
| **On-board measurements** | No | No | No | Yes | No |
| **On-board controller** | Yes | No | No | No | No |
| **Swappable LEDs** | Yes | No | No | Yes | No |
| **Assembly Time** | 2.5 - 6 h | 24 - 72 h | Unknown | Unknown | Unknown |

| | | | | | |
|---|---|---|---|---|---|
| **Approximate Cost** | $150-$400 | $650 | >$500 | $40,000 | Unknown |
| **Open/Closed-Source** | Open | Ambiguous | Ambiguous | Open | Open |
| **Documentation and Instructions** | Comprehensive | Insufficient | Insufficient | Sufficient | Sufficient |

[a] To our knowledge, the fiber optic in this device can address a single well at a time, but can be programmed to move between wells.

[b] Abbreviations: PWM., pulse-width modulation; RGB., red, green blue; GUI., graphical user interface; SI., Scripting Interface; ABS., acrylonitrile butadiene styrene.

[c] Analog intensity controlled with potentiometers, while PWM controlled with a microcontroller.

**Supplementary Table S13.** Flow cytometry settings.

| Parameter | CcaS-CcaR | yMM1081 | Calibration Beads |
|---|---|---|---|
| FSC | E01 | E-1 | E01 |
| SSC | 600 | 300 | 426 |
| SSC-Threshold | 630 | N/A | 400 |
| FSC-Threshold | N/A | 300 | N/A |
| FL1 | 400 | N/A | 400 |
| FL3 | N/A | 790 | 790 |
| Density Gate (FlowCal) | 0.3 | 0.95 | 0.3 |

**Supplementary Table S14.** Strains used in this study and their contact laboratories.

| System | Strain | Lab (PI)[a] | Institute[a] | Ref[a,b] | Note |
|---|---|---|---|---|---|
| CcaS-CcaR (*E. coli*) | JT2 | Tabor | RU | 6 | |
| CcaS-CcaR (*E. coli*) | JT2/pJT119b/pPLPCB(S) | Tabor | RU | 44 | |
| CRY2-CIB1 Y2H (*S. cerevisiae*) | yMM1146 | McClean | UWM | 52 | |
| CRY2-CIB1 Y2H (*S. cerevisiae*) | yMM1146/pGal4AD-CIB1/pGal4BD-CRY2 | McClean | UWM | 52 | Alias: yMM1081 |
| PHYB/VNP-PIF6 (Mammalian) | HEK293T | N/A | ATCC | N/A | #CRL-3216 |
| PHYB/VNP-PIF6 (Mammalian) | HeLa | N/A | ATCC | N/A | #CCL-2 |
| Circadian Rhythm (*S. elongatus* PCC 7942) | AMC462 | Golden | UCSD | 68 | |

[a] Abbreviations: PI., Principal Investigator; Ref., reference; RU., Rice University; UWM., University of Wisconsin, Madison; ATCC., American Type Culture Collection; UCSD., University of California, San Diego.

[b] References are from the main text.

**Supplementary Table S15.** Plasmids used in this study and their accession information.

| System | Plasmid | Repo[a] | Accession # | Ref[a,b] | Note |
|---|---|---|---|---|---|
| CcaS-CcaR (*E. coli*) | pJT119b | Addgene | 50551 | 44 | |
| CcaS-CcaR (*E. coli*) | pPLPCB(S) | N/A | N/A | 44 | |
| CRY2-CIB1 Y2H (*S. cerevisiae*) | pGal4AD-CIB1 | Addgene | 28245 | 11 | |
| CRY2-CIB1 Y2H (*S. cerevisiae*) | pGal4BD-CRY2 | Addgene | 28243 | 11 | |
| PHYB/VNP-PIF6 (Mammalian) | pAAV-GFP | UNCVC[a] | None given | N/A | Serotype 2, CMV promoter |
| PHYB/VNP-PIF6 (Mammalian) | pXX6-80 | NGVB[a] | None given | N/A | |
| PHYB/VNP-PIF6 (Mammalian) | pVP2A-PIF6 | Addgene | 73369 | 54 | |
| PHYB/VNP-PIF6 (Mammalian) | pVP1/3 | Addgene | 73548 | 54 | |
| PHYB/VNP-PIF6 (Mammalian) | pKM017 | N/A | N/A | 18 | |

[a] Abbreviations: Repo., repository; Ref., reference; UNCVC., University of North Carolina Vector Core; NGVB., National Gene Vector Biorepository.

[b] References are from the main text.

## Supplementary Methods

### Circuit board fabrication and assembly

The purpose of this procedure is describe the steps to assemble a functioning LPA circuit board from parts listed in **Supplementary Table S1** using equipment listed in **Supplementary Table S2**. There are two major steps: 1) Fabrication of the unpopulated LPA Printed Circuit Board (PCB), and 2) Assembly, or soldering of the surface mounted and through-hole components. Step 1 is performed via a commercial supplier. For step 2, we have provided do-it-yourself and commercial protocols.

### 1. Fabrication.

The unpopulated LPA PCB can be ordered from a company by providing the six gerber (.gxx) files and one drill (.drl) file in the **Supplementary Files**. There are many companies that provide this service. Companies will manufacture PCBs at different costs per PCB depending on the order size. We have utilized OSH Park (Portland, OR), which has fabricated three LPA PCBs (minimum order) for $82.20. OSH Park does not perform PCB component assembly (see below).

### 2 (option A). Do-it-yourself assembly.

*Surface mounted component soldering:*

1. Tape the printed circuit board (PCB) to a benchtop, and align and tape PCB stencil on top. Smear solder paste over the stencil.
2. Peel the stencil up, leaving solder paste over electrical contacts.
3. Use tweezers to place surface mount components, following the component placement diagram (**Supplementary Files**).
4. Place the populated PCB into a toaster oven and adjust the oven temperature setting according to the following sequence: 150°C for 2 min; 180°C for 1 min; 200°C for 30 s; and 230°C until solder is melted (usually 1-1.5 min). Times indicate when to make the temperature setting adjustment, not when the oven temperature is reached.
5. Open the oven door, pull out the rack and cool for 1 min before moving the board.
6. Inspect the solder connections on the board for bridged or failed connections. Use the soldering kit to add or remove solder where necessary (an inspection microscope is helpful for this step).

*Through-hole component soldering:*

LED socket alignment and soldering is demonstrated in **Supplementary Video S1**. Briefly:

1. Use the 3D printed LED socket aligner (**Supplementary Fig. S7**) to make sure that the LED sockets are positioned and oriented evenly. Use tape to secure LED sockets in place.
2. Invert the circuit board and solder LED sockets to the circuit board using a soldering kit.

Once all components are soldered, use a digital multimeter (DMM) to check the following: (a) there should be no connection between power and ground, (b) the ground pin on the power connector should be conducting with the ground pins on the microcontroller and each of the LED drivers, (c) the 3.3V output of the voltage regulator should be conducting with the power pins on the microcontroller and each of the LED drivers, (d) LED driver pins and associated PCB pads should conduct (good alignment can be tricky).

### 2 (option B). Commercial assembly.

Many companies will assemble surface and through hole components. If you choose this option, you would typically have the same company fabricate and assemble the circuit board. Cost for these services reduces dramatically with order size. For example, Screaming Circuits (Canby, OR) has quoted us fabrication and assembly cost at approximately $950 for one or $1200 for ten boards.

In addition to providing the information necessary for board fabrication (see above), a bill of materials (BOM) and component placement file must be provided (**Supplementary Files**). Companies will charge significantly more for performing assembly on two sides of the board. Because there are only two electrical components on the front layer of the LPA board (excluding LED sockets), we recommend only ordering assembly on the back layer. The male molex connector and reset switch can easily be soldered by hand. In any case, due to the need for the custom LED socket aligner tool, we do not recommend having a company solder the LED sockets. This should be done by hand as described above.

### 3. Powering the circuit board.

Once the circuit board components have been soldered and inspected, the device can be powered on:

1. Construct the barrel-jack power adapter by stripping two ~8 in lengths of multicore wire.

2. Use the molex terminal crimper to attach terminals to the wire, and insert the terminals into the female molex housing.
3. Strip and insert the other ends of the wire into the barrel jack connector, and screw down the terminals to keep the wires fixed.
4. Plug in the AC wall adapter and connect it to the power adapter you have just constructed (but not yet connected to the circuit board).
5. (Important) Make sure that power and ground are in the correct positions with a DMM. The positive terminal has a square pad on the back side of the PCB.
6. Connect the power adapter to the circuit board. Use the back of your index finger to test whether any of the components are heating up. If the components remain cool, then the board has been built properly.

**Firmware programming**

## 1. Installing Atmel Studio

Note: Windows 7 or later is required to install Atmel Studio.

a. To obtain the link to download Atmel Studio, you need to have an account on the Atmel website. Go to https://secure.atmel.com/forms/secure/user-registration.aspx and follow the instructions to set up an account.

b. Go to http://www.atmel.com/tools/ATMELSTUDIO.aspx. Scroll down the page, and click on “Atmel Studio 7.x (build xxx) web installer” to download the installer. If you are not logged in, the website may ask you to do so. If everything went correctly, you should now have the Atmel Studio installer on your computer.

c. Run the installer. You might see a small window saying “Your license for xeam Visual Installer has expired”. This is an issue on Atmel’s end and should not concern the user. Click on Continue.

d. Read and accept the End User License Agreement, and accept the defaults in the next windows. At the end, a confirmation window will appear. Click on “Install”. The installer will now download and install Atmel Studio. This should take around 10 minutes, depending on your connection and computer.

e. When installation is complete, a confirmation window will appear. Leave the checkbox “Launch Atmel Studio” on and click on “Close”. This will test that Atmel Studio can be opened successfully.

f. If everything is correct, the Atmel Studio window will now appear.

## 2. Programming the Device

This procedure requires an Atmel microcontroller programmer, such as the the AVRISP mkII or the AVR Dragon. This procedure also requires the user to download the LPA firmware files from the **Supplementary Files** or the LPA website (http://rice-bioe.github.io/LPA-hardware).

To program the firmware into the LPA’s microcontroller, follow these steps.

a. Make sure that the programmer is correctly connected to the LPA board via the programming connector, and to the PC via USB. Also make sure that the LPA board is connected to a power supply. If you are using the AVRISP mkII programmer, the programming connector is a black 6-pin female connector. This should be plugged into the LPA's male programming connector such that the red edge of the programming wire points outwards, in the opposite direction of the SD card connector.

b. On Atmel Studio, go to Tools -> Device Programming. The "Device Programming" window will appear.

c. Make sure that the appropriate tool is selected (e.g. AVRISP mkII). Also, select "ATmega328" under "Device" and "ISP" under "Interface". Click on the "Apply" button. The window should look similar to **Supplementary Fig. S13**.

d. Click on the "Read" button, next to the "Device signature" box, to the right of the "Apply" button from the previous step. If everything went correctly, the "Device signature" box should be filled, the "Target voltage" should be 3.3V, and no error messages should appear. If there is an error, try selecting ATmega328P under "Device" and repeating from the previous step. If you are still getting an error, close the window and repeat everything from step a. If the error persists, it is likely that the programming connector, microcontroller, and/or power regulator have been soldered incorrectly. Refer to the **Supplementary Method** on circuit board fabrication and assembly.

e. This step is only necessary the first time a new device is programmed. On the "Device Programming" dialog, select "Fuses" from the list on the left. On the "fuse name/value" list that will open, make sure that the CKDIV8 box is disabled, the CKOUT box is enabled, and the SUT_CKSEL box is set to EXTXOSC_8MHZ_XX_1KCK_14CK_65MS. Click on the "Program" button below.

f. Select "Production file" from the list on the left in the "Device Programming" dialog. Click on the "…" button located to the right of the "Program device from ELF production file" textbox. Locate file "firmware.elf" inside the "firmware" folder in the **Supplementary Files**. Click on "Open".

g. Make sure that the "Flash", "Erase memory before programming", and "Verify programmed content" checkboxes are activated, and click on the "Program" button. The messages "Erasing device... OK", "Programming Flash...OK", and "Verifying Flash...OK" should appear below.

## Firmware compiling

This procedure lists the steps required to properly compile the firmware's source code. It is only recommended if the user intends to modify the firmware for a very particular application. For regular use, the user is advised to use the precompiled version included with the **Supplementary Files** (See **Supplementary Method** on firmware programming).

This section assumes that the user has installed AtmelStudio and the version control software git.

1. **Installing the Arduino Libraries**

   The firmware uses the Arduino libraries to read from the SD card. In order to have access to those libraries, the user must obtain a compiled copy of the Arduino libraries and include them in the firmware project inside Atmel Studio. The following procedure must be followed:

a. Go to https://www.arduino.cc/en/Main/Software and download the Arduino Integrated Development Environment (IDE) Windows installer.

b. Run the installer. Accept the License Agreement, and the defaults in the following windows. The installer will install the Arduino IDE. Click on "Close" when installation is finished.

c. Open the Arduino IDE. We need to enable verbose mode to locate the compiled libraries. Go to File -> Preferences, and check "Show verbose output during compilation". Click on the "OK" button.

d. We must select a board that uses the ATMega328, which is the microcontroller used in the LPA. Go to Tools -> Board, and make sure that "Arduino Uno" is selected.

e. Compile the default sketch (or any other sketch) by going to Sketch -> Verify/Compile. The black bottom panel in the window will be filled with messages. One of the last messages will start with something similar to "C:\Users\{Your User Name}\AppData\Local\Temp\build3717660540954573100.tmp/". This is the path of the folder in which the Arduino IDE has placed the compiled Arduino libraries. Open this folder on Windows Explorer.

f. We need to create a folder to store the compiled libraries. We will do it in the Atmel Studio working directory (by default it is “My Documents\Atmel Studio”). Open this folder and create a directory called “arduinoCore328”.

g. Go back to the folder that you opened in step e. The file “core.a” contains the compiled Arduino libraries. Copy this file into the arduinoCore328 folder and rename it to libcore.a. The arduino library is now ready.

## 2. Downloading and Building the Firmware

a. The user first needs to obtain the source files. From a command line terminal run the following:

```
git clone --recursive https://github.com/rice-bioe/LPA-hardware
```

This should create a folder called “LPA-hardware”.

b. Navigate to “LPA-hardware\firmware”. Open the file “firmware.atsln”. This will open the firmware project in Atmel Studio.

c. Open the project properties by clicking on Project -> firmware Properties (or pressing Alt + F7). The project properties panel will open. Click on “Toolchain” on the left (**Supplementary Fig. S8**).

d. On the list in the middle, navigate to AVR/GNU Linker -> Libraries (**Supplementary Fig. S8**).

e. There is one item on the Library search path panel, with the text %HOMEPATH%\Documents\Atmel Studio\arduinoCore328 (**Supplementary Fig. S8**). You need to change this to the folder that you created in steps 1f and 1g. Select this item and click on the “Edit item” button (**Supplementary Fig. S8**).

f. A small window should appear. Enter the path of the folder created in steps 1f and 1g, and uncheck the “Relative path” checkbox. Click on “OK”.

g. Go to Build -> Rebuild Solution (or press Ctrl + Alt + F7) to build the project. No errors (messages with red icons on the “Output” panel at the bottom) should occur. Warning messages (yellow exclamation icons) are acceptable. A file called

“firmware.elf” inside the “LPA-hardware/firmware/Debug” folder should have been created. You can program this file into the LPA as in the **Supplementary Method** on firmware programming.

3. **Troubleshooting Compilation:**

If step 2g fails, follow the steps below, one at a time, and try step 2g again.

a. Go to Project -> firmware Properties, or press Alt + F7. The project properties panel will open. Click on “Toolchain” on the left. Navigate to “AVR/GNU C Compiler -> Miscellaneous”. Check that “-include ../config.h” (without the quotation marks) is added to the “Other flags” textbox (**Supplementary Fig. S9**). Repeat the same for “AVR/GNU C++ Compiler -> Miscellaneous”.

b. Go to Project -> firmware Properties, or press Alt + F7. The project properties panel will open. Click on “Toolchain” on the left. Navigate to “AVR/GNU C Compiler -> Directories”. A list of directories called “Include Paths” will be shown (**Supplementary Fig. S10**). Check that these entries point to valid locations. As for Arduino 1.6.5, the following directories must be included:

   i. “{Arduino Installation folder}\hardware\arduino\avr\cores\arduino”: Contains several header files (*.h) and source files (*.cpp), including “Arduino.h”.

   ii. “{Arduino Installation folder}\hardware\arduino\avr\variants\standard”: Contains “pins_arduino.h”.

   iii. “{Arduino Installation folder}\libraries\SD\src”: Contains the source files of the SD card reader library, including “File.cpp”, “SD.cpp”, and “SD.h”, among others.

   iv. “{Arduino Installation folder}\libraries\SD\src\utility”: Contains several more source files and headers of the SD card library, such as “Sd2Card.h”, “SdFat.h”, and "SdInfo.h”.

   v. “{Arduino Installation folder}\hardware\arduino\avr\libraries\SPI”: Contains source files and headers of the SPI library, a module that is necessary to communicate with the SD card. Files included are “SPI.cpp” and “SPI.h”, among others.

The default installation folder is “C:\Program Files (x86)\Arduino\”. If you installed Arduino to a different folder, change the entries in this list to reflect that. It is possible that in a version of Arduino different than 1.6.5 the individual folders have been moved within the Arduino installation folder. If the locations listed above are invalid, you should locate the corresponding folders that match the descriptions above. To modify an entry, select it from the list and click on the “Edit item” button (**Supplementary Fig. S10**).

When you finish, go to “AVR/GNU C++ Compiler -> Directories” on the middle panel and repeat this step.

c. Check that the necessary source files from the Arduino SD card library are included for compilation. The following files from the Arduino library should be referenced:

    i. “File.cpp “. Default location: “C:\Program Files (x86)\Arduino\libraries\SD\src\File.cpp“

    ii. “SD.cpp “. Default location: “C:\Program Files (x86)\Arduino\libraries\SD\src\SD.cpp”

    iii. “Sd2Card.cpp “. Default location: “C:\Program Files (x86)\Arduino\libraries\SD\src\utility\Sd2Card.cpp”

    iv. “SdFile.cpp “. Default location: “C:\Program Files (x86)\Arduino\libraries\SD\src\utility\SdFile.cpp”

    v. “SdVolume.cpp “. Default location: “C:\Program Files (x86)\Arduino\libraries\SD\src\utility\SdVolume.cpp”

    vi. “SPI.cpp “. Default location: “C:\Program Files (x86)\Arduino\hardware\arduino\avr\libraries\SPI\SPI.cpp”

If you installed Arduino to a different folder than “C:\Program Files (x86)\Arduino\”, the references need to be changed to reflect this. It is possible than in a version of Arduino different than 1.6.5, the files are located in different folders within the Arduino installation folder. If a reference is not found, it will have an exclamation icon next to its name in the Solution Explorer panel in Atmel Studio (**Supplementary Fig. S11**). To correct this, do the following for each missing file:

    1. Obtain the true location of the file in the Arduino installation folder.

2. On the Solution Explorer in Atmel Studio, right click on the problematic file and select “Remove”.

3. Go to “Project -> firmware Properties”, and then to “Project -> Add existing item”.

4. A dialog will open to select a file. Navigate to the true location of the file and select it. Click on the arrow next to the “Add” button (lower right of the dialog) and select “Add as link”.

## LED Installation and reconfiguration

The purpose of this procedure is to describe how to install and reconfigure LEDs on the circuit board. The circuit board and LED sockets are designed for use with two-lead 5mm LEDs. Before loading LEDs into LED sockets, the LED leads are trimmed to approximately 0.25 inches with wire cutters. LEDs are loaded by hand into the LED sockets by guiding LED leads through socket holes and pushing firmly to ensure LEDs are fully seated and correctly oriented (**Supplementary Fig. S15**). LED polarity can be determined from visual inspection of the circuit elements inside the LED lens. For reconfiguration, LEDs can be removed by hand and their position preserved (for later reinstallation) by placing them in wells of any 24-well culture plate.

## Laser cutting gaskets

The purpose of this procedure is to describe the steps for fabricating or commercially obtaining laser cut gaskets for the LPA (**Supplementary Fig. S18**). Gasket patterns were designed in SolidWorks, converted to .DXF format, and are available in the **Supplementary Files**. Gaskets were fabricated from nitrile rubber sheets (**Supplementary Table S6**) using the Rice University Oshman Engineering Design Kitchen laser cutter set to vector cutting at 100% power, 5% speed, and 500 pulses per inch (PPI) (Universal Laser Systems, X-660, 60W). Laser cutting this material produces residues which need to be thoroughly washed off the gaskets. In most cases, soap, water, and scrubbing is sufficient. Sometimes the nitrile rubber sheets come coated in an oil which makes soap and water ineffective. In this case we coat and scrub the gaskets with castor oil, wash with soap and water, dry, and rinse with 100% ethanol; repeating as necessary.

### *Laser cutting gaskets yourself*

Each of the three LPA gaskets can be laser cut by converting the part's corresponding .DXF file (units are in mm) to a file format compatible with your particular laser cutter and cutting with the settings listed above (based on 60W laser).

### *Obtaining gaskets from a commercial supplier*

Gaskets can be obtained from a commercial source by providing a laser cutting company with the gasket's corresponding .DXF file (**Supplementary Files**) and gasket material information (**Supplementary Table S6**). You will usually have the option to mail them the gasket material or have the company obtain it themselves (40% markup on materials is common).

We have successfully trialed gasket fabrication with Pololu Robotics and Electronics, who cut one set of gaskets for $40 (not including material and shipping cost).

**3D printing LPA parts**

The purpose of this procedure is to describe steps for fabricating or commercially obtaining 3D printed parts for the LPA. 3D printed parts were created in SolidWorks and converted to g-code using ReplicatorG software. SolidWorks and .STL files for each part can be found in the **Supplementary Files**. Prints were made with black ABS plastic, 10% infill, 0.2 mm layer height, full support, and no raft.

*3D printing LPA parts yourself*

Each LPA part can be 3D printed by converting the part's corresponding .STL file to G-code using "slicing" software and using the print settings listed above.

During printing, part warping at the edges was problematic due to the large profile of the objects. To counteract object warping, we found it helpful to print on a fresh kapton tape surface and without the use of a raft (lattice of ABS printed underneath the object). For the LED spacer in particular, we found it necessary to print the object on top of a thin layer of ABS cement. ABS cement is made by dissolving ABS plastic (we use discarded ABS parts) in 100% acetone until the solution has roughly the consistency of coffee creamer. Before starting the print, the solution is applied to the build surface with a swab in the area of the object's perimeter and allow the acetone to evaporate. Using cement throughout the entire area of the object should be avoided, as it will be extremely difficult to remove the object when complete.

The device LED sockets fit very snuggly within the LED spacer's socket ports. The exact dimensions of the LED spacer's socket ports are sensitive to the 3D printer's build plate height which may need to be fine-tuned.

*Obtaining 3D Printed Parts from a commercial supplier*

3D printed parts can be obtained commercially by providing the part's corresponding .STL file and specifying print settings. We recommend two different options for commercial fabrication.

More expensive option: Utilize a professional 3D printing service to print high quality and consistent LPA parts. We successfully trialed Xometry, which printed the four LPA parts and LED socket aligner for about $300 with the following options: fused deposition modelling, black ABS-M30, light infill.

Less expensive option: Utilize a service, such as MakeXYZ, which will outsource the fabrication to independent 3D printer owners/hobbyists. Results will almost certainly depend on the 3D printer, but you can screen them for quality. We have not tested this option, but the cost is significantly less (about $120 for the four LPA parts and LED socket aligner).

## LPA final assembly

The purpose of this procedure is to describe steps for assembling the LPA from its constituent parts. Images of the LPA chassis parts used in this procedure can be found in **Supplementary Fig. S16**. This procedure is also demonstrated in **Supplementary Video S2**.

*Circuit board-spacer module assembly:*

First the circuit board gasket is installed on to the PCB. It is best to load all LEDs before mating the circuit board with the LED spacer. The circuit board and LED spacer snap together by guiding the circuit board's LED sockets through the spacer's socket ports. The correct orientation can be determined by examining the geometries of the sockets and socket ports. The LED sockets should fit very snuggly within the spacer's socket ports and they can initially be difficult to snap together. The socket ports on the LED spacer have a "breaking in" period and it can be helpful to snap together the parts several times at the bench before calibration and experimenting. Often there are one or several LED sockets that are slightly misaligned with their port. A small screw driver can be used to nudge the socket in place during mating with the LED spacer. When the circuit board and spacer have been snapped together, they form a module which does not have to be disassembled unless maintenance or reconfiguration of LEDs is necessary. After assembling the circuit board-spacer module, it is a good idea to push down the LEDs into their sockets again with a soft tool to ensure proper seating and orientation of LEDs.

*Mounting plate installation:*

The mounting plate couples the device to a platform and can be secured if shaking is required. First, mounting bolts (**Supplementary Table S6**) are installed on the mounting plate by guiding them from the bottom through the outer bolt holes and firmly pushing the hex head into the recessed opening. The mounting plate is then secured (if necessary) to the platform with the appropriate platform. The mounting plate can now accept the modules that make up the rest of the device.

*Mounting the device modules:*

Final device assembly is accomplished by mounting the device modules and layers onto the mounting plate. The order in which modules/layers are added is: circuit board-spacer module > spacer gasket > plate adapter > plate adapter gasket > cell culture plate (with adhesive foil) > device lid. The device is then compressed and secured by applying wing nuts (**Supplementary Table S6**) to the mounting bolts and hand tightening.

*Powering the device:*

The device accepts DC 5V which is supplied via a wall mounted power adapter, barrel jack adapter, and 2x1 molex connection (**Supplementary Table S1**). The barrel jack and molex connection have a tendency to be interrupted when shaking or moving the wires. Therefore, we use standard laboratory tape to secure the wires and connections to prevent mechanical stress and movement at these points (**Supplementary Fig. S6**).

## Calibrating LEDs

The purpose of this procedure is to describe our method for calibrating LEDs for experiments with LPAs. We calibrate devices to produce the same photon flux across LEDs. We use two methods to calibrate LEDs depending on the type of LED configuration: an image analysis method for calibrating LEDs of the same wavelength (e.g. twenty-four 647 nm LEDs), and a probe spectrometer method for calibrating LEDs of different wavelength (e.g. action spectrum LED sets). Before calibration, it is critical that LEDs be firmly pushed down into their sockets to ensure proper LED height and alignment. For both methods the device is assembled from the mounting plate up to the circuit board-spacer module. During calibration only one channel, i.e. only one LED per well, should be on at a time.

LED compensation is achieved by setting the grayscale and dot correction for each LED. These values are distinct from the intensity values set in Iris. Grayscale and dot correction values are stored on the device's SD card as files "gcal.txt" and "dc.txt", respectively, and must be space delimited integers from 0-255 and 0-63, respectively. Coarse adjustments can be made by setting the LED dot correction, while fine adjustments can be made setting the gray scale value.

Note that the grayscale adjustment reduces the resolution of intensities which can be achieved. With a maximum grayscale adjustment value of 255, there are 4096 intensity levels which can be programmed. However, the number of intensity levels available reduces proportionally to the grayscale adjustment value. For example, if the grayscale adjustment value is set to 100, there will only be 4096 * 100/255 = 1606 programmable intensity levels. This reduction in resolution means that when programming values in Iris, multiple programmed values will correspond to the same resulting intensity. Continuing the example, a programmed value of 4095 will be converted to 1606 internally in the LPA, while the programmed values of 4095, 4094, and 4093 will all correspond to 1605.

*Image analysis method:*

We developed an image analysis method for calibrating LEDs of the same wavelength to one another. The method is relatively fast and utilizes a MATLAB script (**Supplementary Files**) which analyzes pixel intensity of images taken top-down of the device while all LEDs in a channel (top row or bottom row) are set to a constant intensity. We had success taking top-down images of devices in an enclosed gel-imager (FluorChem FC2, Alpha Innotech) which blocks out ambient light. Briefly, we set all LEDs within a channel to a single Iris intensity below the saturation threshold (but ≥ 100, see above) of the gel imager camera. To reduce the spatial structure of the LED light we place three sheets of diffuser paper (#3008, Rosco) atop the LED spacer and add the plate adapter. The device is centered below the camera and powered on. Finally, images (60ms exposure) are taken at 0° (well 1 in top corner) and at 180° (well 1 in

bottom right, i.e. transposed). Images are then analyzed by the MATLAB script. A HowTo which contains details of running the script as well as example images and script outputs is available in the **Supplementary Files**. After calibration by image analysis, photon flux is determined by measuring a subset (≥ five LEDs) of the calibrated LEDs with a probe spectrometer (see below).

*Probe spectrometer method:*

The image analysis method does not work for calibrating LEDs of different wavelength to each other due to the camera's differential sensitivity to different wavelengths of light. For action spectra LED configurations, we directly measure the photon flux of each LED using a probe spectrometer (StellarNet Inc, photodetector: EPP2000 UVN-SR-25LT-16, probe: F600-UV-VIS-SR, software: SpectraWiz) (**Supplementary Fig. S20**), and 3D printed probe adapter (**Supplementary Fig. S7**). The probe adapter is designed to align the probe directly above an LED within a well at the same height as the bottom of the cell culture plate. During calibration, the device is programmed with a constant, sub-saturating intensity (usually between 10-20μmol $m^{-2}$ $s^{-1}$) across each LED being calibrated. A typical probe calibration workflow is as follows:

1. Coarse adjust LED output to the approximate desired intensity by measuring photon flux while adjusting dot correction. Do this one LED at a time.
2. Measure photon flux for all LEDs. The measurement can be performed using the peak-fitting function within SpectraWiz, or can be made by saving the spectrum and calculating the integral of the peak from the spectral flux data.
3. Calculate:

$$GS_{desired} = \frac{Intensity_{desired}}{Intensity_{current}} GS_{current}$$

   and enter the desired gray scale values for each LED.

4. Repeat steps 2-3 until desired photon flux and variance across LEDs is achieved.

*LPA time step calibration*

The resonator (535-10008-1-ND) used with the current device design has a tolerance which produces a small time error. For long experiments, this can become significant. To correct for this time scaling, one can adjust the `stepSizeScaling` (**Supplementary Fig. S12**) in the firmware config.h file. This parameter (set to 1.0 by default) will scale the time steps of the programs run on the device. The correction is best done by running a long (12 h <) program on the device and noting (by indicator LEDs or well LEDs) the actual length of the experiment. The `stepSizeScaling` then should be set to the ratio of desired time to actual time.

## Supplementary Notes

## LPA firmware description

### 1. Introduction:

The LPA firmware is an embedded program written in C for the ATmega328 microcontroller. It performs the following main functions:

a. Reads an LPF file from an SD card, connected to the microcontroller via a Serial Peripheral Interface (SPI).
b. Reads light intensity ("grayscale") values from the LPF file.
c. Updates the intensity of a set of LEDs under control of TLC5941 drivers, by transferring the appropriate grayscale values via SPI.

The firmware has been divided in different modules that perform separate functions. All the relevant variables inside a module are named as <module_name>_<variable_name>. Functions are named in a similar format. Modules are written such that, when possible, the assignment of hardware resources is defined by a "configuration" step, controlled by a separate config.h file that is included during compilation. This facilitates porting the individual modules to different projects, or transferring this project to a similar microcontroller.

In this section, we will describe the high-level behavior of the firmware main program, the contents of each module, and the config.h file.

### 2. States of the Firmware Program

The LPA will often need to respond differently to the same stimuli, especially under error conditions. This is best managed by modeling the system as a finite state machine, in which the device's behavior is a function of both the inputs and the current state. In the firmware, the current state is contained in a single variable. **Supplementary Table S4** enumerates the different states of the device, and **Supplementary Fig. S14** shows the transition diagram between different states. Each pattern displayed by the status LEDs corresponds directly to each state (**Supplementary Tables S3** and **S4**).

### 3. TLC5941 Driver module

The TLC5941 driver module is available as a separate project (https://github.com/castillohair/Tlc5941_library). This module is heavily based on the "Demystifying the TLC5940" book[10], with a few additions to make the library work with the TL5941 instead of the TL5940, the Atmega328 USART module instead of the SPI module, among others. In short, this module incorporates functions to:

- Initialize the TLC5941
- Set grayscale values, one at a time or all at once.
- Change dot correction values, one at a time or all at once.

In addition, several precompiler flags are available to define the pins used to interact with the TLC5941. These can be changed inside “config.h” file without modifying the library’s source code.

## 4. MsTimer Module

The MsTimer (millisecond timer) module incorporates functions to define a timer that calls a set of functions (“callbacks”) periodically, with a period specified in milliseconds. This module uses the Atmega’s Timer1 to generate interrupts via compare events. The module incorporates functions to

- Initialize the relevant registers of Timer1.
- Add callbacks and their respective periods in milliseconds.
- Start and stop the module.

## 5. StatusLeds Module

The StatusLeds module is a simple module that abstracts the toggling of status LEDs on the board. It incorporates functions to

- Initialize the pins that control the status LEDs.
- Set the status of an LED to on or off.
- Toggle an LED to the opposite state.

## 6. SD Card Reading

SD card reading is performed using the Arduino SD card library. For details on how to set the library before compiling, refer to the **Supplementary Method** on firmware programming.

## 7. The config.h File

The `config.h` file should be included during compilation of every file associated with the project. By doing this, preprocessor directives defined in `config.h` (i.e. `#define` instructions) replace the ones defined in the modules by default. This allows us to override the default behavior of the module without changing the code, a procedure called “configuration”.

Additionally, the firmware has been made such that an arbitrary number of LEDs in any arrangement is possible. The number of LEDs is specified through the number of LED drivers (`Tlc5941_N`), whereas the arrangement is specified as a constant array (`well2channel`). The arrangement corresponding to the 24-well LPA is included by default, but a file called “`config_96.h`”, which illustrates the configuration for a hypothetical 96-well LPA, is also included.

## 8. firmware.cpp

This file contains the main function of the firmware (`main()`). Other important parts are:

- Variables `dotCorrectionValues[]` and `grayscaleCalibration[]`, which store dot correction and grayscale calibration values after reading them from files dc.txt and gcal.txt in the SD card.
- Definitions of constants corresponding to the states (**Supplementary Table S4**, all the `#define System_<state_name>` instructions), a variable containing the current state (`System_state`), and functions to evaluate the state (`System_SetState()`, `System_IsState()` and `System_IsNotState()`).
- A function to update the LPA LEDs, `UpdateLeds()`.
- A function to update the status LEDs based on the current state, `UpdateStatusLeds()`.

In short, the main function does the following:

1. Initializes the TLC5941 module.
2. Initializes the MsTimer and the StatusLeds module.
3. Initializes the SD card module.
4. Reads and parses both dc.txt and gcal.txt files.
5. Opens the “program.lpf” file.
6. Reads the time step size specified in “program.lpf”, and initializes timers with MsTimer so `UpdateLeds()` is called with the appropriate frequency.
7. Waits for `UpdateLeds()` to be called.
8. Reads a new set of intensity values from the SD card and places them on a buffer, such that `UpdateLeds()` can send them to the TLC5941s when it is called again.
9. Repeat from 6 until “program.lpf” file has been read completely.

## Running Iris Offline

Iris should be accessable online, but it can be run offline as well. To do so, follow these steps:

1. Download the Iris code from the **Supplementary Files** or GitHub repository and decompress it.
2. Start a local HTTP server. This can be done using many tools, but a simple way uses Python:
    i. Open a command line / terminal window and navigate to the folder containing the Iris code.
    ii. Execute the command: python -m SimpleHTTPServer to begin the HTTP server.
    iii. The terminal window will then indicate which local port it is serving pages from, probably port 8000. Take note of this port number.
3. To initiate an Iris session, open a browser and navigate to http://localhost:XXXX, where XXXX is the port number the HTTP server is using.

## Iris waveform handling

*Adding vs. Combining Constant Waveforms:*

Several waveforms (Constant and Step) are able to take multiple inputs, which are then automatically expanded by Iris into a number of wells. The default behavior when more than one of these waveforms is entered in a particular Experiment is for every combination of the intensities specified to be created. For example, if Constant Waveform 1 indicates 2 intensities for the red LED (123, 234 GS) and Constant Waveform 2 indicates 2 intensities for the green LED (1234, 2345 GS), then 4 wells will be used: (123, 1234), (123, 2345), (234, 1234), and (234, 2345) for the R/G LED intensities, respectively. This makes it very easy to specify a series of arbitrary intensities for one LED, while keeping another LED constant in all wells: Waveform 1 would indicate the arbitrary intensities, and Waveform 2 would only need a single intensity, which would then be applied to the arbitrary wells. We refer to this result as a Combination of waveforms.

Alternatively, some experiments require arbitrarily chosen LED intensities in more than one channel. Instead of creating a separate Experiment for each set of intensities in a particular well, Iris can be programmed to integrate multiple Constant waveforms differently: Addition. Rather than creating every combination of input intensities, Iris will associate lists of intensities in an element-wise fashion. For example, in the same scenario as above, the result will be only 2 wells: (123, 1234) and (234, 2345). Note that for Addition, the lengths of the lists of intensities must be equal. Additionally, a Constant Waveform cannot be Added to any other type of (dynamic) waveform -- when a dynamic waveform is added to the Experiment, Iris automatically defaults to the above Combination behavior.

*Iris Waveforms:*

The four icons in an Experiment represent the four fundamental waveform inputs programmed into Iris: constant, step change, sinusoid, and arbitrary, which can be added by clicking these icons. Each Waveform represents a light input applied to the desired wells in a particular LED channel. Importantly, waveforms cannot be composed - that is, multiple waveforms cannot be applied to the same LED in the same well. More complex inputs (e.g. a series of step inputs) should be entered using the (more efficient) Arbitrary Waveform. Note that all light intensities (amplitudes) are given in hardware greyscale units (GS), which must be in the range [0,4095] Also note that if multiple intensities are given to the Constant or Step Waveforms, each intensity will be separately applied to every other waveform in the experiment, since multiple intensities of a single LED cannot be applied to the same well. In other words, every possible combination of amplitudes is used. For example, if 2 intensities are entered in a Step Waveform (e.g. 1000GS & 2000GS), and the Experiment specifies 10 samples ("time points") and 1 replicate, the Experiment will use 20 wells in the plate.

*Constant Waveform:*

$$f(t) = c$$

Constant inputs are used to apply competing amounts of deactivating light and to measure the steady-state dose response function. Obviously, they only have a single input parameter.

*Step Waveform:*

$$f(t) = a\,H(t-\tau) + c$$

Step inputs (i.e. Heaviside step; H(t)) are used for dynamic characterization and have 3 parameters:

- Amplitude (a): the size of the step change, in GS units. *Note that step amplitudes can be negative! This indicates a step-down.*
- Step offset (c): the vertical offset of the step function in GS units (constant addition across all time points)
- Time shift (τ): the amount of time (min) after the beginning of the experiment that the step should happen. Note: this is different from the Experiment parameter regarding the delay until the first time point! This is specifying a change in the light input for this waveform; the delay until the first time point is specifying a change in the staggered-start for all wells in the Experiment.

*Sine Waveform:*

$$f(t) = a\ \sin\left(\frac{2\pi(t-\phi)}{T}\right) + c$$

Sinusoidal inputs are an alternative input signal for dynamic characterization and have 4 parameters:

- Amplitude (a): The amplitude of the sine (half the peak-to-peak amplitude) in GS units
- Period (T): The period of the wave, in minutes; the inverse of the wave frequency
- Phase (ϕ): The phase shift of the wave, in minutes
- Offset (c): The vertical offset of the wave, in GS units

*Arbitrary Waveform:*

$$f(t) = \sum_{0}^{n} a_i H(t - \tau_i)$$

Arbitrary Waveforms allow input of any more complex function as a series of light intensities (a_i) and corresponding times at which the LED will switch to that intensity (τ_i). These are entered as a list of values in the Excel-like table under their respective headings. The switch times are the time since the beginning of the experiment (not related to time points), in minutes. The light intensities are in greyscale (GS) units. Note that because the smallest time resolution for the resulting LPF file is 1 sec, this is also the smallest valid time step for arbitrary inputs; time steps smaller than one second will be rounded up to the nearest whole second during LPF creation.

### Iris randomization and de-randomization procedure

The values in the Randomization Matrix (RM) are the true positions in the plate of the data for a particular well. For example, if the first value (index 0) in the RM is 16, then the true data for the first well is in the well with index 16 (well number 17).

Example Python code to perform this de-randomization:

```
# Example Randomization Matrix
rand_mat = [4, 0, 1, 3, 2]
# Toy measured data for wells: 0, 1, 2, 3, 4
measured_data = [234, 345, 567, 456, 123]
# Empty; will hold the descrambled data values
descrambled_data = [0, 0, 0, 0, 0]
total_well_num = 5
for i in range(total_well_num):
    descrambled_data[rand_mat[i]] = measured_data[i]
print descrambled_data
## Prints:
## [123, 234, 345, 456, 567]
```

## Writing an LPF using Python

Occasionally, users comfortable with coding may want to quickly create algorithmic LPF files based on custom code outside of Iris. To facilitate this, a simple python script (requires Numpy) has been added that can do just this. It will be maintained in parallel with any changes to the header information & LPF format in the main Iris code. To create an LPF in this way, users will have to ensure that their data is in a Numpy matrix with the correct dimensionality (indices refer to: [Time][wellNumber][channelNum]). The user is entirely responsible for ensuring that their matrix matches the device they have chosen to use. The second input parameter is a dictionary of device parameters for the header of the LPF: 'channelNum' is the total number of channels (channels per well * number of wells); 'timeStep' is the time step in ms; 'numSteps' is the total number of time steps in the LPF. Finally, the given file name is the complete (relative) path to the desired file location AND the desired file name, including suffix (.lpf).

## Supplementary Video Legends

**Supplementary Video Legend S1.** LED socket alignment and soldering.

Video tutorial showing use of the LED socket aligner (**Supplementary Fig. S7**) to align and then solder LED sockets to the LPA circuit board.

**Supplementary Video Legend S2.** Assembling and powering the LPA.

Video tutorial showing the final assembly and powering on of an LPA.

**Supplementary Video Legend S3.** Iris steady-state mode.

Video tutorial showing light program creation in Iris using steady-state mode. Google and the Google logo are registered trademarks of Google Inc., used with permission.

**Supplementary Video Legend S4.** Iris dynamics mode.

Video tutorial showing light program creation in Iris using dynamics mode. Google and the Google logo are registered trademarks of Google Inc., used with permission.

**Supplementary Video Legend S5.** Iris advanced mode.

Video tutorial showing light program creation in Iris using advanced mode. Google and the Google logo are registered trademarks of Google Inc., used with permission.

## Supplementary References


1. Atmel, “Atmel 8-bit microcontroller with 4/8/16/32kbytes in-system programmable flash” Atmel-8271I-AVR- ATmega-Datasheet, Oct. 2014.
2. F. Foust, “Secure Digital Card Interface for the MSP430” Appl. Note, 2004
3. Atmel, “AVR910: In-System Programming” Rev. 0943E Appl. Note 935, Aug. 2008
4. Texas Instruments, “16-Channel LED driver with DOT correction and grayscale PWM control” SLVS589D datasheet, Jul. 2005 [Revised Sept. 2008].
5. STMicroelectronics, “Adjustable and fixed low drop positive voltage regulator” DocID2572 Rev 33, Nov. 2013.
6. Olson, E. J., Hartsough, L. A., Landry, B. P., Shroff, R. & Tabor, J. J. Characterizing bacterial gene circuit dynamics with optically programmed gene expression signals. *Nat. Methods* **11,** 449–55 (2014).
7. Davidson, E. A., Basu, A. S. & Bayer, T. S. Programming microbes using pulse width modulation of optical signals. *J. Mol. Biol.* **425,** 4161–6 (2013).
8. Richter, F. *et al.* Upgrading a microplate reader for photobiology and all-optical experiments. *Photochem. Photobiol. Sci.* **14,** 270–9 (2015).
9. Hannanta-anan, P. *et al.* Optogenetic Control of Calcium Oscillation Waveform Defines NFAT as an Integrator of Calcium Load. *Cell Syst.* **2,** 283–288 (2016).
10. M. Pandina, *Demystifying the TLC5940*, 2004. [E-book]
