# Supplementary material for: An open-hardware platform for optogenetics and photobiology
Source: Sci Rep. 2016 Nov 2;6:35363. doi: 10.1038/srep35363 (PMC5096413; doi:10.1038/srep35363)
Supplement: Supplementary Files [file srep35363-s2.zip › Supplementary Files/Iris/documentation/changelog.html]

# Iris Change Log

A log of recent improvements, changes, and known issues for Iris. Note that new issues may always be submitted to the devs via email (iris-devs at rice dot edu) or on the GitHub Repo. All version numbers loosely follow Semantic Versioning.

## [0.5.0] - Unreleased

### Added

- Preset input styles: steady-state, dynamic, and advanced, for more efficient data input.
- This changelog. Initial version number (0.5.0) selected so that previous versions can be back-dated if desired.

### Changed

- Step waveform input parameters changed from (amplitude, offset, step time) to (low intensity, high intensity, step time) for clarity.

### Fixed

- CSS rendering issues

### Known Issues

- Input validation (#273) and well deselection (#275) are broken on custom input devices. In fact, many things about custom devices may be broken.
