# Supplementary material for: An open-hardware platform for optogenetics and photobiology
Source: Sci Rep. 2016 Nov 2;6:35363. doi: 10.1038/srep35363 (PMC5096413; doi:10.1038/srep35363)
Supplement: Supplementary Files [file srep35363-s2.zip › Supplementary Files/Iris/documentation/documentation.html]

# Iris: The Light Program Interface

A web application designed to enable easy programming and creation of Light Program Files (LPFs) for use in Tabor Lab optogenetic hardware. It uses HTML5 and JavaScript to acquire desired light function parameters, perform intensity & staggered-start calculations, and finally deliver output files to the user, all in the browser.

## Introduction

Iris is designed to be a flexible interface for the Tabor Lab standardized optogenetic hardware platform. It is designed to:

1. Program optogenetic time courses in the hardware by compiling the desired Light Program File for a particular device,
2. Help with optogenetic program design and debugging with a full hardware (LED) simulation display,
3. Serve as an experimental support utility by creating files documenting a particular experiment's well randomization positions, desired time points, etc., in a CSV file,
4. Create a convenient record of previous optogenetic experiments by generating an .irs file that can be later used to reload a particular light program in Iris.

Note that for advanced users, there is also a standalone Python script that can convert more complex and arbitrary light programs into device-readable LPF files; however Iris should be sufficient for all normal use cases.

## Contents

- Getting Started
  - Select an optogenetic device from the dropdown menu
  - Select Input Style
  - Enter global experimental parameters
  - Deactivate Undesired Wells
  - Add a New Experiment
    - Timepoints
    - Replicates
    - Adding vs. Combining Constant Waveforms
  - Add waveforms to the Experiment
    - Constant Waveform
    - Step Waveform
    - Sine Waveform
    - Arbitrary Waveform
  - Load and assess the hardware simulation
    - Plate View
    - Well View
  - Download the output files
- Detailed Nuts and Bolts
  - De-randomization Procedure
  - The Staggered Start Algorithm
  - LPF File Specifications
- Packages
- Supported Browsers
- Running Iris Offline
- Writing an LPF Using Python
- Issues, Bugs, and Pull Requests
- License

## Getting Started

### 1. **Select an optogenetic device from the dropdown menu**

A variety of devices are supported in addition to those detailed in our publication, though the most common selection will be the 24-well plate device (LPA). This will automatically configure Iris to have the correct number of wells and correct LED wavelengths for simulation later. If you have a custom device running appropriate firmware, then you can use the **Custom Configuration**, which will prompt you to enter the number of rows and columns in your custom device, as well as the number of LEDs in each well and their wavelengths in the section that appears.

### 2. **Select Input Style**

There are three input styles that can be used to create light programs: **Steady State**, **Simple Dynamic**, and **Advanced Dynamic**. Each will significantly change the programming layout for creating the corresponding programs, as described below:

- **Steady State** programs are the simplest way to create a static light program in which LED intensities are constant. The intensity of each LED in each well is set using a spreadsheet.
- **Simple Dynamic** programs allow specification of dynamic light waveforms (step, sine, and arbitrary) in addition to constant waveforms, each indicated by their pictograms. This input style is intended for use in experiments where dynamic time points will be taken manually throughout the experiment by directly sampling the cultures in the wells; therefore, *the staggered start algorithm is not used*. Additionally, the specified waveform(s) will be repeated identically on all wells in the device.
- **Advanced Dynamic** programs should be used for all other cases. This input style allows arbitrarily-spaced (or evenly-spaced) time points to be specified for groups of wells referred to as Experiments. Using the staggered-start algorithm, each well is assigned to a time point and its waveform is shifted accordingly, such that the entire plate can be removed and iced simultaneously to acquire dynamic response data. Additional options are also available, including well randomization, and the ability to add any number of Experiments.

### 3. **Enter global experimental parameters**

Some parameters apply to the entire experiment: the total program time duration (in minutes), whether wells should be programmed column-wise or row-wise, whether the well positions should be randomized, and whether the LEDs should all be turned off at the end of the experiment (check boxes). The **Program Duration** should include all phases of the experiment, including any dark or preconditioning phases, which will be specified later. If you choose to randomize the well positions (highly recommended), the generated randomization matrix will be provided when you download the LPF so that you can descramble the data during analysis. We recommend turning off the LEDs at the end of the experiment, since this serves as a convenient indicator that the program has run its complete course.

**Note:** These parameters do not apply to the **Steady-State** input style. Only the Program Duration applies to the **Simple Dyanamic** input style.

### 4. **Deactivate Undesired Wells**

If there are wells in the device that should not be programmed, these can be eliminated from Iris' calculations by right clicking them in the simulation panel. They will be marked by a large X and will be skipped as Iris fills wells. These wells will be programmed to keep their LEDs off for the entire length of the experiment. The selection of eliminated wells may be updated at any point during the Iris session.

### 5. **Add a New Experiment**

In the **Advanced Dynamic** input style, Experiments define groups of wells in the plate device that are related, typically because they are all time points or measurements of the same dynamic experiment (i.e. all wells are receiving versions of the same input signal with staggered start times). An Experiment can utilize any number of wells in the plate, and the number used by a particular Experiment will automatically be updated as inputs are entered. All Experiments (and Waveforms) can be minimized at any time during input to make room for additional input elements by clicking the chevron to the left of the Experiment header.

**Note:** All the parameters below are set automatically in the **Steady State** and **Simple Dynamic** input styles.

#### *Time points*

In an **Advanced Dynamic** program, Iris can automatically generate a set of evenly-spaced time points or use a custom set (to focus data on early-time responses, for example). For generated time points, enter the number of desired time points and the delay (in minutes) until the first time point, if any. For custom time points, simply paste a list of comma-separated time points into the array (all units in minutes). **Steady-state experiments should use the default value of 1 for the number of time points.**

#### *Replicates*

Enter the number of experimental replicates of this experiment. The number of wells specified by the Experiment's Waveform inputs will be replicated across the plate `Replicates` number of times (i.e. `Replicates = 1` indicates that *no additional* wells will be used). Note that this will very quickly consume available wells.

#### *Adding vs. Combining Constant Waveforms*

Several waveforms (Constant and Step) are able to take multiple inputs, which are then automatically expanded by Iris into a number of wells. The default behavior when more than one of these waveforms is entered in a particular Experiment is for every combination of the intensities specified to be created. For example, if Constant Waveform 1 indicates 2 intensities for the red LED (123, 234 GS) and Constant Waveform 2 indicates 2 intensities for the green LED (1234, 2345 GS), then 4 wells will be used: (123, 1234), (123, 2345), (234, 1234), and (234, 2345) for the R/G LED intensities, respectively. This makes it very easy to specify a series of arbitrary intensities for one LED, while keeping another LED constant in all wells: Waveform 1 would indicate the arbitrary intensities, and Waveform 2 would only need a single intensity, which would then be applied to the arbitrary wells. We refer to this result as a **Combination** of waveforms.

Alternatively, some experiments require arbitrarily chosen LED intensities in more than one channel. Instead of creating a separate Experiment for each set of intensities in a particular well, Iris can be programmed to integrate multiple Constant waveforms differently: **Addition**. Rather than creating every combination of input intensities, Iris will associate lists of intensities in an element-wise fashion. For example, in the same scenario as above, the result will be only 2 wells: (123, 1234) and (234, 2345). Note that for Addition, the lengths of the lists of intensities must be equal. Additionally, a Constant Waveform cannot be Added to any other type of (dynamic) waveform -- when a dynamic waveform is added to the Experiment, Iris automatically defaults to the above Combination behavior.

### 6. **Add Waveforms to the Experiment**

For **Dynamic** input styles, the four icons represent the four fundamental waveform inputs programmed into Iris: constant, step change, sinusoid, and arbitrary, which can be added to the Experiment by clicking the corresponding icons. Each Waveform represents a light input applied to the desired wells in a particular LED channel. Importantly, **waveforms cannot be composed** - that is, multiple waveforms cannot be applied to the same LED in the same well. More complex inputs (e.g. a series of step inputs) should be entered using the (more efficient) Arbitrary Waveform. Note that all light intensities (amplitudes) are given in hardware greyscale units (GS), which must be in the range \([0,4095]\). Also note that if multiple intensities are given to the Constant or Step Waveforms, **each intensity will be separately applied to every other waveform in the experiment**, since multiple intensities of a single LED cannot be applied to the same well. In other words, every possible combination of Waveforms is programmed. For example, if 2 intensities are entered in a Step Waveform, and the Experiment specifies 10 samples ("time points") and 1 replicate, the Experiment will use 20 wells in the plate.

#### *Constant Waveform*

\[f(t) = c\] Constant inputs are used to apply competing amounts of deactivating light and to measure the steady-state dose response function. Obviously, they only have a single input parameter.

#### *Step Waveform*

\[f(t) =\begin{cases}I\_o & t < t\_s\\I\_f & t \geq t\_s\end{cases}\] Step inputs are used for dynamic characterization and have 3 parameters:

- **Initial Intensity (\(I\_o\))**: The intensity before the step, in GS units.
- **Final Intensity (\(I\_f\))**: The intensity after the step, in GS units.
- **Step Time (\(t\_s\))**: The time (in min) when the step occurs.

#### *Sine Waveform*

\[f(t) = a \* \sin \left(\frac{2\pi \left(t - \phi\right)}{T}\right) + c\] Sinusoidal inputs are an alternative input signal for dynamic characterization and have 4 parameters:

- **Amplitude (\(a\))**: The amplitude of the sine (half the peak-to-peak amplitude) in GS units
- **Period (\(T\))**: The period of the wave, in minutes; the inverse of the wave frequency
- **Phase (\(\phi\))**: The phase shift of the wave, in minutes
- **Offset (\(c\))**: The vertical offset of the wave, in GS units

#### *Arbitrary Waveform*

\[f(t) = \sum\_{i=0}^n a\_i H(t-\tau\_i)\] Arbitrary Waveforms allow input of any more complex function as a series of light intensities (\(a\_i\)) and corresponding times at which the LED will switch to that intensity (\(\tau\_i\)). These are entered as a list of values in the Excel-like table under their respective headings. The switch times are the time since the beginning of the experiment (not related to time points), in minutes. The light intensities are in greyscale (GS) units. Because the smallest time resolution for the resulting LPF file is 1 sec, this is also the smallest valid time step for arbitrary inputs.

### 7. Load and assess the hardware simulation

To load a simulation of the specified light program, first ensure that no input fields have been marked as invalid. A tooltip will appear on mouse-over to indicate the relevant error for a particular field, if it is invalid. (Errors in Arbitrary or Steady State input tables are highlighted by red cells.) If the inputs for each Experiment are valid, Iris will automatically load a hardware simulation in the right hand panel. This simulation has two aspects: **Plate View**, which is an overview of LED intensity over time for the entire plate; and **Well View**, which displays a light time-course plot for all LEDs in a particular well.

#### Plate View

The default view is **Plate View**, which shows an overview of the entire plate device. Using the drop-down menu in the navigation bar at the top, the display can be limited from showing all (illuminated) LEDs to only particular LEDs. Clicking on a well in the plate visualization will select that well, updating the position and well number in the nav bar. The up, down, left, and right arrow keys can also be used to change the selected well. Clicking the play button will begin the hardware simulation, and will show the response of the plate device to the generated light program over time. The Speed slider bar will decrease or increase the simulation playback speed.

#### Well View

To get more detail about a particular well, simply click the well in Plate View and then click `Well View` in the nav bar. **Well View** plots the LED intensity for all LEDs in a well as a function of time. Click and drag horizontally in the plot area to zoom in on that region of the plot. Notice that, again, the smallest time resolution for the program is 1 sec, which will cause some apparent aliasing at small timescales. To remove the plot for an LED, simply click its entry in the legend. A floating tooltip indicates the LED intensities corresponding to the time currently under the mouse cursor. The arrow keys will still change the selected well and can be used to rapidly move through adjacent wells' Well Views.

### 8. Download the output files

If everything looks good, then initiate the download of the generated files by clicking the **Download** button at the bottom of the inputs. The zipped folder includes the following files:

- **program.lpf** This is the hardware-readable Light Program File that will be loaded onto an SD card, which will then be processed by the plate hardware into LED intensities. Its file structure is detailed below, but is basically a binary composed of a short header and a series of intensities at each time point in the experiment. **The file name of this file must remain unchanged in order to be read correctly by the firmware.**
- **randomizationMatrix.csv** This CSV contains all the information necessary for analysis after the experiment is completed:
  - **Program [Well] Index**: List of well numbers, top to bottom, left to right
  - **Plate Coordinates**: List of plate coordinates (A1, A2, etc) that clarify the locations of the Well Indices
  - **True Well Location**: Also known as the randomization matrix; randomized positions of the corresponding wells in the Well Index column. See below for the correct de-randomization algorithm.
  - **Time Points**: Since each well corresponds to a staggered-start time point in a dynamic run, these are listed here. Steady state programs (i.e. wells with only constant waveforms) will use the total Program Duration as the Time Point. Wells with no light program will have an 'undefined' time point.
- **savefile.irs** This file represents a complete image of the exact Iris session (including all inputs) used to generate the LPF file. This file can be used to reload an Iris session at a later date. Its primary function is to enable modification of a previous light program for future experiments and as a record for exactly what the corresponding LPF encodes. Note that the randomization matrix is also stored in this file.

#### IMPORTANT

It is crucial that these files not get separated from each other. **Without an IRS file, it is impossible (using Iris) to determine what program a particular LPF file encodes, and the hardware does not allow this file to be renamed!** That said, it is possible to parse LPF files using scripts (see to the standalone Python code.) Instead, however, we recommend simply keeping these files together and loading up a IRS file to reload exactly the Iris session that produced the corresponding LPF.

Furthermore, loss of the randomization matrix will make the data impossible to analyze. **There is no way to extract the randomization matrix from the LPF program, though it can be recovered (re-downloaded) from the Iris session savefile.**

## Detailed Nuts and Bolts

### De-randomization Procedure

If the corresponding box is checked, Iris will randomize the positions of all wells in the plate. The Randomization Matrix (RM) can be accessed the CSV file of the same name in the downloaded zip folder. In plain language, the values in the RM represent the true (descrabled) positions in the plate of the data for a particular well. For example, if the first value (index 0) in the RM is `11`, then the data from the first well in the plate (index 0) should be moved to the well with index 11 (well number 12). *Be careful not to do this backwards!*

To perform this de-randomization in Excel, simply add a new column to the randomization matrix CSV containing data values from each well (match to Well Number / Plate Coordinates). Next, sort the CSV data by the Randomization Matrix column. The data values will now be de-randomized and can be plotted against the Time Points column.

Some example Python code to perform this de-randomization:

```
    rand_mat = [4, 0, 1, 3, 2] # Example Randomization Matrix
    measured_data = [567, 123, 234, 456, 345] # Toy measured data for wells: 0, 1, 2, 3, 4
    descrambled_data = [0, 0, 0, 0, 0] # Empty; will hold the descrambled data values
    total_well_num = 5
    for i in range(total_well_num):
        descrambled_data[rand_mat[i]] = measured_data[i]
    print descrambled_data
    ## Prints:
    ## [123, 234, 345, 456, 567]
```

### The Staggered Start Algorithm

In order to perform dynamic light experiments, the input signal applied to each well in an experiment is staggered such that at the end of the program, that well will end at the desired time point in the waveform, as previously validated using our test-tube based Light Tube Array (Olson *et al.*, Nature Methods, 2014). The time difference created by staggering the input is filled by exposing the well to the Preconditioning light condition (table below) for all times before the time-shifted waveform begins (*red overlay*). For example, the t=560min time point (Well 5, below) in a 720min experiment will experience the Preconditioning light condition for 160min, and then begin the staggered program. It will experience the first 560min of the waveforms in each of its LED channels, at which point the experiment will end. This procedure is repeated for all wells (time points) in an experiment, and can be visualized in Iris under Well View.

**Schematic of the staggered-start algorithm.** The schematic above demonstrates how the staggered-start algorithm is used to produce light time courses corresponding to desired time points in an experiment for each dynamic waveform in Iris: **(a)** a step input, with the step occurring at t=100min; **(b)** a sine waveform with 360min period; and **(c)** an arbitrary waveform. The plots demonstrate this process for 6 equally-spaced time points (subplots corresponding to the right axis) on each dynamic waveform. In this example, Well 1 corresponds to the t=0min time point, and therefore experiences the Precondition input (*red overlay*) for the entire experiment, while Well 6 corresponds to the t=720min time point, and experiences no preconditioning.

More specifically, the Preconditioning light input for each function is as follows:

| Input Waveform | Precondition Value |
| --- | --- |
| Constant | N/A |
| Step | Input intensity before step (i.e. the step offset, \(c\)) |
| Sine | Because sines are periodic, no precondition state is necessary; the function is simply phase shifted by the appropriate amount. |
| Arbitrary | The precondition light intensity for Arbitrary waveforms is set by the user in the waveform input spreadsheet. |

### LPF File Specifications

The LPF binary should not need to be examined directly during the standard workflow; however, its format is detailed below should it be necessary:

The LPF file has a header segment encoded by 32-bit (4-byte) ints:

| Bytes | Variable Name | Precondition Value |
| --- | --- | --- |
| 0-3 | FILE\_VERSION | LPF version number, currently `1` |
| 4-7 | NUMBER\_CHANNELS | number of channels -- **Note:** This is the total number of LED channels (e.g. `2*24 = 48`), not per well |
| 8-11 | STEP\_SIZE | time step size, in ms (default: 1000ms to limit total program file size) |
| 12-15 | NUMBER\_STEPS | number of time points (total program time / STEP\_SIZE + 1) |
| 16-31 | --empty-- | Reserved space for future header fields; all set to 0 |
| >= 16 | n/a | intensity values of each channel per time point. For each value, two bytes will be used as a long 16-bit int |

LED intensity values are listed for a single time point in a depth-first manner (i.e. all LEDs for a particular well, then proceeding to the next well), moving top-to-bottom, and left-to-right across the plate device. The LED order is a hard-coded parameter for each device, and is dependent on the particular configuration of the PCB. After the final LED intensity of the final well, the LPF continues with the next time step.

Because of the above structure, specifically that every time step is encoded explicitly, and that each is encoded using a 16-bit (2 byte) integer, file sizes can quickly become very large at small time steps or long program lengths. **To keep things reasonable, we limit time steps to 1 sec, minimum. The time step is automatically increased to 10s for LPF programs longer than 12hr.**

## Packages

The following packages and utilities were used in the creation of Iris:

- jQuery & jQuery UI
- Handsontable
- jsZip
- Numeric Javascript
- CanvasJS [Noncommercial License; no changes made]
- FileSaver [License]
- Angular JS
- Pandoc (for creating this documentation)
- MathJax (via Pandoc)
- Bourbon CSS
- SASS CSS
- 720kb's Angular Tooltips

The standalone Python LPF Encoder script uses Numpy and was made using iPython Notebook.

## Supported Browsers

Iris should be fully functional on all up-to-date versions of: Chrome & Safari. The LPF file size limit, set by the FileSaver.js package, is limited by Chrome, which only allows files smaller than 500MB.

## Running Iris Offline

Iris should be accessable online, but it can be run offline as well. To do so, follow these steps:

1. Download the Iris code from the GitHub repository and decompress it.
2. Start a local HTTP server. This can be done using many tools, but a simple way uses Python:
   1. Open a command line / terminal window and navigate to the folder containing the Iris code.
   2. Execute the command: `python -m SimpleHTTPServer` to begin the HTTP server.
   3. The terminal window will then indicate which local port it is serving pages from, probably port 8000. Take note of this port number.
3. To initiate an Iris session, open a browser and navigate to `http://localhost:XXXX`, where `XXXX` is the port number the HTTP server is using.

## Writing an LPF using Python

*Requires Numpy.*

Occasionally, users comfortable with coding may want to quickly create algorithmic LPF files based on custom code outside of Iris. To facilitate this, a simple python script has been added that can do just this. It will (hopefully) be maintained in parallel with any changes to the header information & LPF format in the main Iris code.

To create an LPF in this way, users will have to ensure that their data is in a Numpy matrix with the correct dimensionality (indices refer to: [Time][wellNumber][channelNum]). The user is entirely responsible for ensuring that their matrix matches the device they have chosen to use. The second input parameter is a dictionary of device parameters for the header of the LPF: 'channelNum' is the TOTAL number of channels (channels per well \* number of wells); 'timeStep' is the time step in ms; 'numSteps' is the total number of time steps in the LPF. Finally, the given file name is the complete (relative) path to the desired file location AND the desired file name, including suffix (.lpf).

## Issues, Bugs, and Pull Requests

The easiest way to submit a bug report or pull request is to email iris-devs at rice dot edu. Alternatively, Iris is an open-source project; therefore, the GitHub repository housing all Iris code is available for contributions. Any bugs identified can be logged in the project's Issues section, and proposed improvements can be submitted as Pull Requests.

---

## License (MIT License)

Copyright (c) 2015, Felix Ekness, Lucas Hartsough, Brian Landry. All rights reserved.

Redistribution and use in source and binary forms, with or without modification, are permitted provided that the following conditions are met:

1. Redistributions of source code must retain the above copyright notice, this list of conditions and the following disclaimer.
2. Redistributions in binary form must reproduce the above copyright notice, this list of conditions and the following disclaimer in the documentation and/or other materials provided with the distribution.
3. Neither the name of Rice University, nor Iris, nor the names of its contributors may be used to endorse or promote products derived from this software without specific prior written permission.

THIS SOFTWARE IS PROVIDED BY THE COPYRIGHT HOLDERS AND CONTRIBUTORS "AS IS" AND ANY EXPRESS OR IMPLIED WARRANTIES, INCLUDING, BUT NOT LIMITED TO, THE IMPLIED WARRANTIES OF MERCHANTABILITY AND FITNESS FOR A PARTICULAR PURPOSE ARE DISCLAIMED. IN NO EVENT SHALL THE COPYRIGHT HOLDER OR CONTRIBUTORS BE LIABLE FOR ANY DIRECT, INDIRECT, INCIDENTAL, SPECIAL, EXEMPLARY, OR CONSEQUENTIAL DAMAGES (INCLUDING, BUT NOT LIMITED TO, PROCUREMENT OF SUBSTITUTE GOODS OR SERVICES; LOSS OF USE, DATA, OR PROFITS; OR BUSINESS INTERRUPTION) HOWEVER CAUSED AND ON ANY THEORY OF LIABILITY, WHETHER IN CONTRACT, STRICT LIABILITY, OR TORT (INCLUDING NEGLIGENCE OR OTHERWISE) ARISING IN ANY WAY OUT OF THE USE OF THIS SOFTWARE, EVEN IF ADVISED OF THE POSSIBILITY OF SUCH DAMAGE.
