# Supplementary material for: An open-hardware platform for optogenetics and photobiology
Source: Sci Rep. 2016 Nov 2;6:35363. doi: 10.1038/srep35363 (PMC5096413; doi:10.1038/srep35363)
Supplement: Supplementary Files [file srep35363-s2.zip › Supplementary Files/Iris/html/const.html]

Constant Waveform

For LED


Intensities (comma separated list)
