# Supplementary material for: An open-hardware platform for optogenetics and photobiology
Source: Sci Rep. 2016 Nov 2;6:35363. doi: 10.1038/srep35363 (PMC5096413; doi:10.1038/srep35363)
Supplement: Supplementary Files [file srep35363-s2.zip › Supplementary Files/Iris/html/experiment.html]

Experiment
Wells in Use:
{{experiment.getWellCount()}}

Number of Timepoints


Delay Until 1st Timepoint

or
Custom Timepoints

Number of Replicates 

Combine Constant Waveforms
Add Constant Waveforms
