# Supplementary material for: An open-hardware platform for optogenetics and photobiology
Source: Sci Rep. 2016 Nov 2;6:35363. doi: 10.1038/srep35363 (PMC5096413; doi:10.1038/srep35363)
Supplement: Supplementary Files [file srep35363-s2.zip › Supplementary Files/Iris/index.html]

Iris


Iris
Tabor Lab
Docs
Change Log
Bug?
0.6.2

Select Device

Steady State

Dynamic

Advanced

Program Duration (min) 

Fill By Rows
Fill By Columns

Randomize the positions 
Turn off LEDs upon finish

New Experiment

Download

{{plateView ? 'Well View' : 'Plate View'}}

All LEDs

Row:  {{selectedRow+1}}

Col:  {{selectedCol+1}}

Well #: {{selectedWell()+1}}

Speed


{{prettyTime}}
