# Supplementary figures and images for: An open-hardware platform for optogenetics and photobiology
Source: Sci Rep. 2016 Nov 2;6:35363. doi: 10.1038/srep35363 (PMC5096413; doi:10.1038/srep35363)

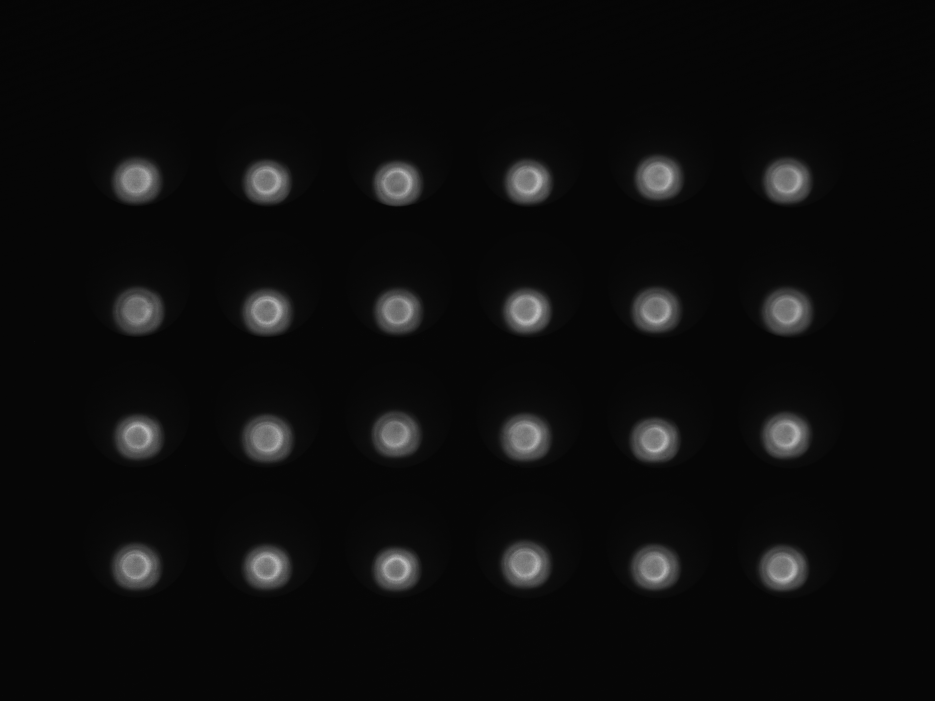

Supplement: Supplementary Files [file srep35363-s2.zip › Supplementary Files/Image analysis script/example/arya_678nm_bot_150802/round1.tif]

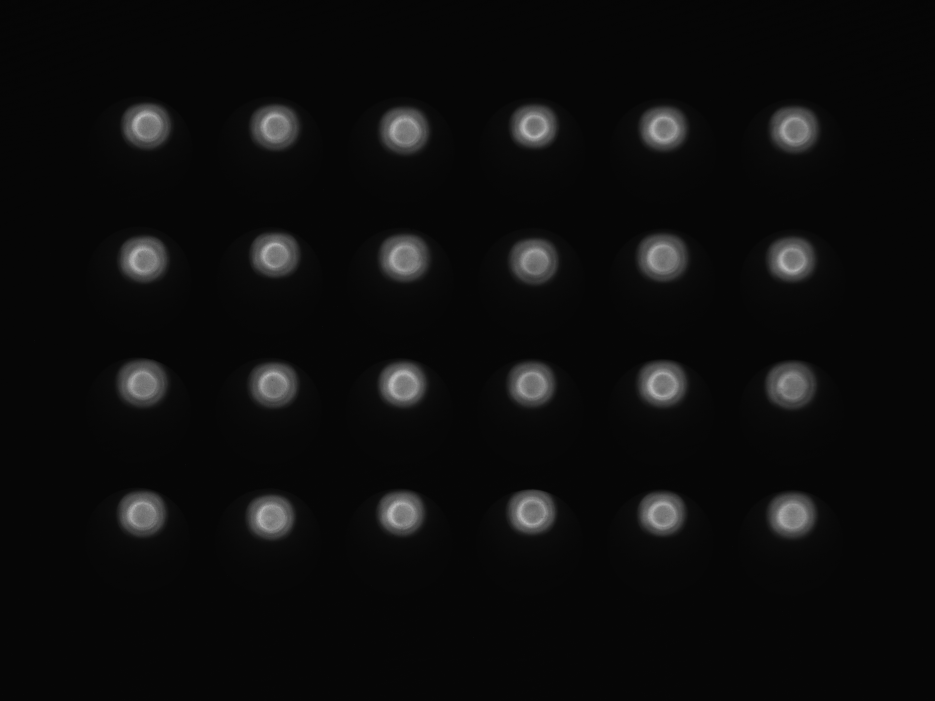

Supplement: Supplementary Files [file srep35363-s2.zip › Supplementary Files/Image analysis script/example/arya_678nm_bot_150802/round1_T.tif]

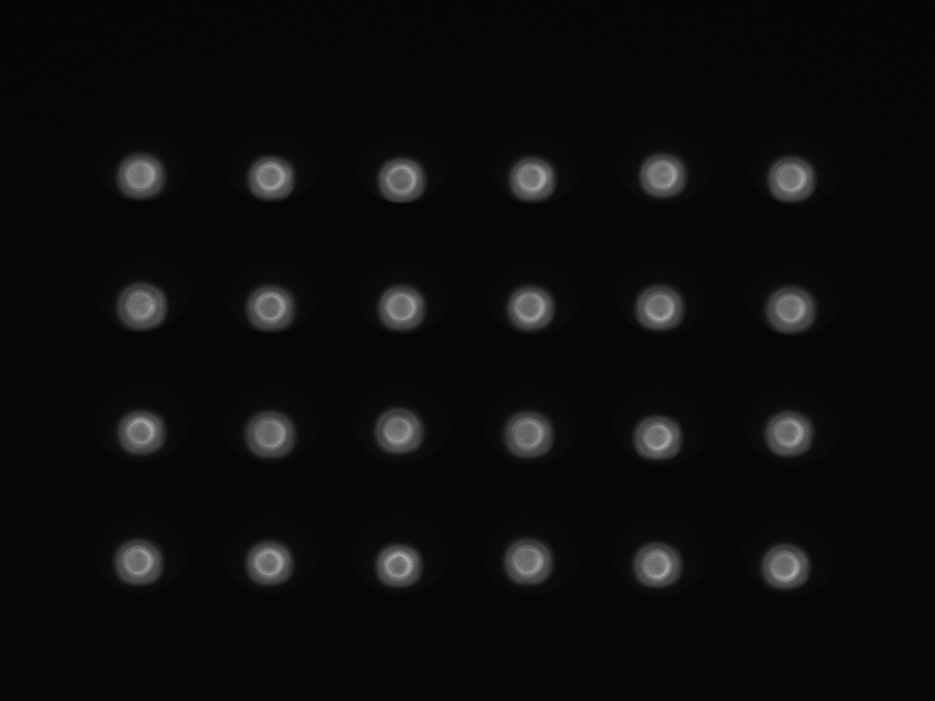

Supplement: Supplementary Files [file srep35363-s2.zip › Supplementary Files/Image analysis script/example/arya_678nm_bot_150802/round2.tif]

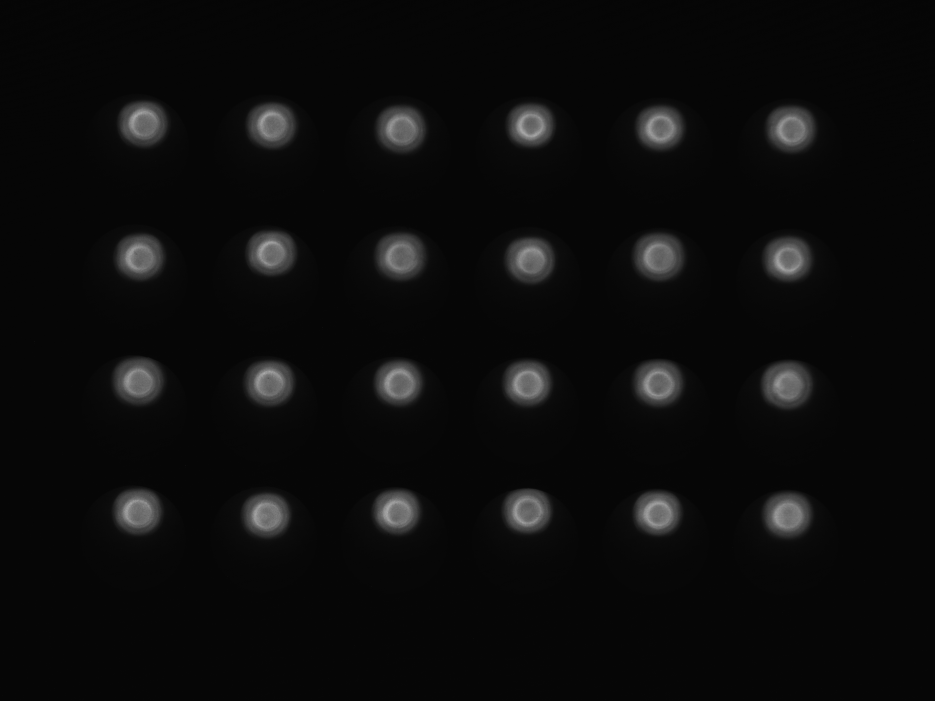

Supplement: Supplementary Files [file srep35363-s2.zip › Supplementary Files/Image analysis script/example/arya_678nm_bot_150802/round2_T.tif]

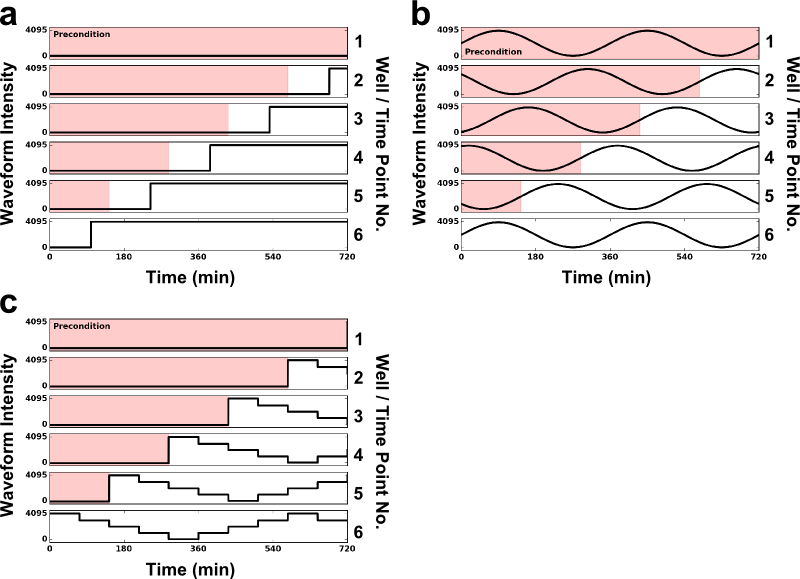

Supplement: Supplementary Files [file srep35363-s2.zip › Supplementary Files/Iris/documentation/Staggered_Start_FigureS12_small.png]

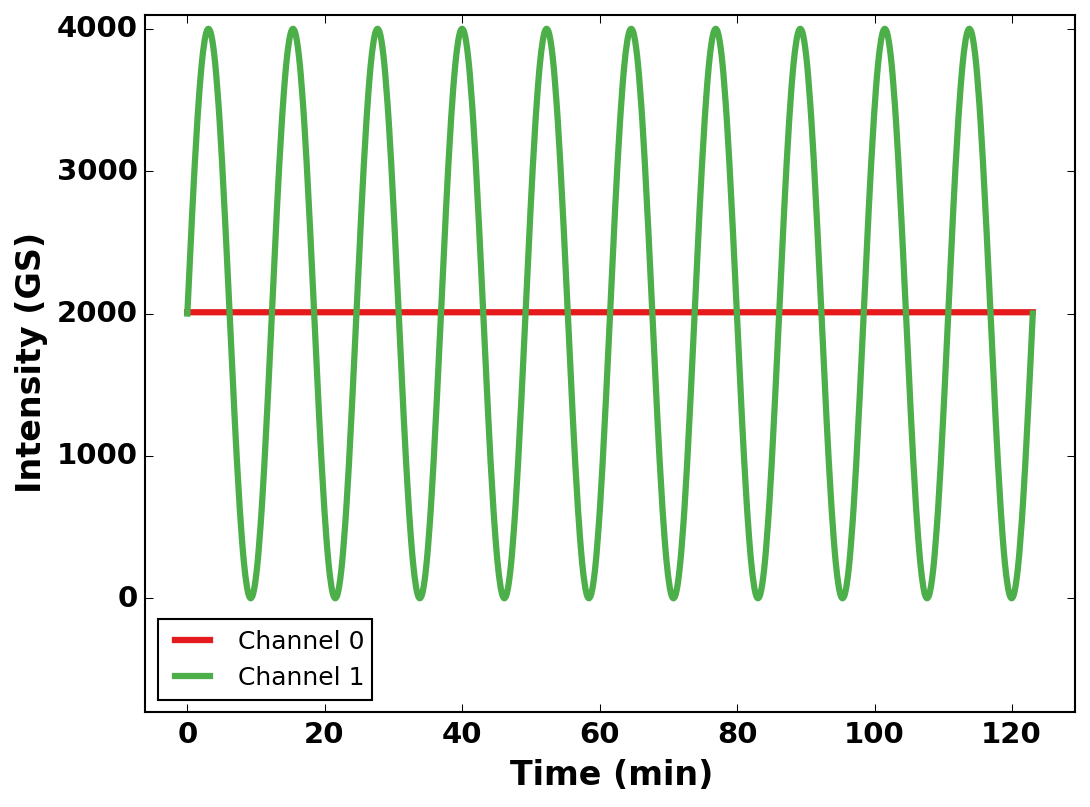

Supplement: Supplementary Files [file srep35363-s2.zip › Supplementary Files/Iris/Python/LPF_plot.png]
